# Synthesis and Oxidative Transformations of New Chiral Pinane-Type γ-Ketothiols: Stereochemical Features of Reactions

**DOI:** 10.3390/molecules26175245

**Published:** 2021-08-29

**Authors:** Olga M. Lezina, Svetlana N. Subbotina, Larisa L. Frolova, Svetlana A. Rubtsova, Denis V. Sudarikov

**Affiliations:** Institute of Chemistry, FRC “Komi Scientific Centre”, Ural Branch of the Russian Academy of Sciences, Pervomayskaya St. 48, 167000 Syktyvkar, Komi Republic, Russia; lezina-om@yandex.ru (O.M.L.); dernova1970@rambler.ru (S.N.S.); frolova-ll@chemi.komisc.ru (L.L.F.); rubtsova-sa@chemi.komisc.ru (S.A.R.)

**Keywords:** monoterpenoids, ketones, sulfurorganic compounds, chlorine dioxide, stereochemistry

## Abstract

Chiral γ-ketothiols, thioacetates, thiobenzoate, disulfides, sulfones, thiosulfonates, and sulfonic acids were obtained from β-pinene for the first time. New compounds open up prospects for the synthesis of other polyfunctional compounds combining a biologically active pinane fragment with various pharmacophore groups. It was shown that the syntheses of sulfanyl and sulfonyl derivatives based on 2-norpinanone are characterized by high stereoselectivity in comparison with similar reactions of pinocarvone. The conditions for the preparation of diastereomerically pure thioacetyl and thiobenzoyl derivatives based on pinocarvone, as well as for the chemoselective oxidation of γ-ketothiols with chlorine dioxide to the corresponding thiolsulfonates and sulfonic acids, were selected. The effect of the VO(acac)_2_ catalyst on the increase in the yields of thiosulfonates was shown. A new direction of the transformation of thiosulfonates with the formation of sulfones was revealed. In the case of pinocarvone-based sulfones, the configuration is inversed at the C2 atom. An epimerization scheme is proposed.

## 1. Introduction

Terpenes possess natural chirality and biological activity. They are a promising raw material for obtaining modified compounds that combine a biologically active terpene fragment with various pharmacophore groups [1]. The introduction of a sulfur atom of various oxidation states into a monoterpene molecule often increases the antifungal, anti-inflammatory, anti-helicobacter, antitumor, and other types of activities of native terpenes and also expands the spectrum of biological activity [2,3].

Biological activity and reactivity directly depend on the molecular geometry of monoterpenoids. Due to the lability of the structure of these substrates, reactions with their participation can proceed in unexpected directions. Therefore, the investigation of the reactivity of monoterpenoids is relevant both for fundamental chemistry and for fine organic synthesis. The use of catalysts that change the activation energy of transformations or reagents that create steric hindrances can significantly increase the chemo-, regio-, and stereoselectivity of the processes [4].

Previously, we synthesized various chiral sulfanyl-, sulfinyl-, and sulfonyl derivatives based on β-pinene containing functional groups such as double bonds, hydroxy groups, etc. [5,6,7,8]. The influence of the molecular geometry of the substrate on the direction, chemo- and stereoselectivity of reactions was shown. It was revealed that pinane thiosulfonates containing hydroxyl group exhibit antimicrobial activity against *Candida albicans*, *Staphylococcus aureus*, and *Cryptococcus neoformans* [5].

Chiral pinane sulfur-containing derivatives with a carbonyl group are not described in the literature; therefore, their preparation and study of the chemical features of the synthesis are relevant and promising.

## 2. Results and Discussions

### 2.1. Synthesis of Pinane γ-Ketothiols and Disulfides

In this work, pinane γ-ketothiols were synthesized for the first time by the addition of thioacids at the double bond of α-, β-unsaturated carbonyl compounds (thia-Michael addition) with subsequent deacylation of the resulting thiocarboxylates.

**Scheme 1:**
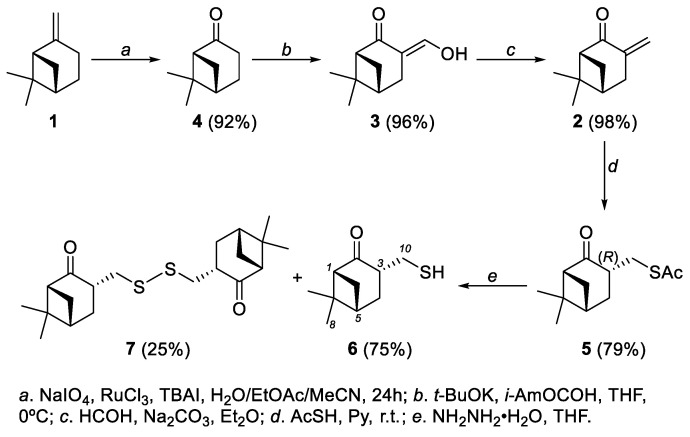
Synthesis of *cis*-3-sulfanylmethylnopinone derivatives.

2-Norpinanone **2** was obtained by multistep synthesis from (−)-β-pinene **1** according to the procedure [9], in which the stage of obtaining ketoenol **3** was modified (Scheme 1). The synthesis of ketoenol **3** from nopinone **4** was carried out in the presence of the *t*-BuOK base instead of NaNH_2_ [9] in THF at 0 °C, and then the system was treated with isoamyl formate, while the yield was increased from 71 to 96%, and the reaction time was reduced from 15 to 6 h without boiling.

The addition of thioacetic acid to 2-norpinanone **2** was carried out according to the procedure [10] in the presence of pyridine as a catalyst [11]. We used THF as a solvent during the optimization of the synthesis conditions for thioacetate **5**. The reaction temperature was increased from −5 °C to room temperature, which led to a significant increase in the rate of acylation (from 3 h to 10 min) while maintaining diastereoselectivity. The main product of the reaction was the isomer (3*R*)-**5** (below **5**) (*de* 98%) (Scheme 1).

Deacylation of thioacetate **5** with hydrazine hydrate (NH_2_NH_2_·H_2_O) leads to 2-ketothiol (3*R*)**-6** (below **6**) and disulfide (3*R*)**-7** (below **7**) in the ratio of 3:1, respectively. Due to the use of NH_2_NH_2_·H_2_O as a deacylating reagent, the reaction proceeds chemoselectively, and the carbonyl group is not reduced to the hydroxyl group [12] as it happens during the deacylation with LiAlH_4_ [8].

**Scheme 2:**
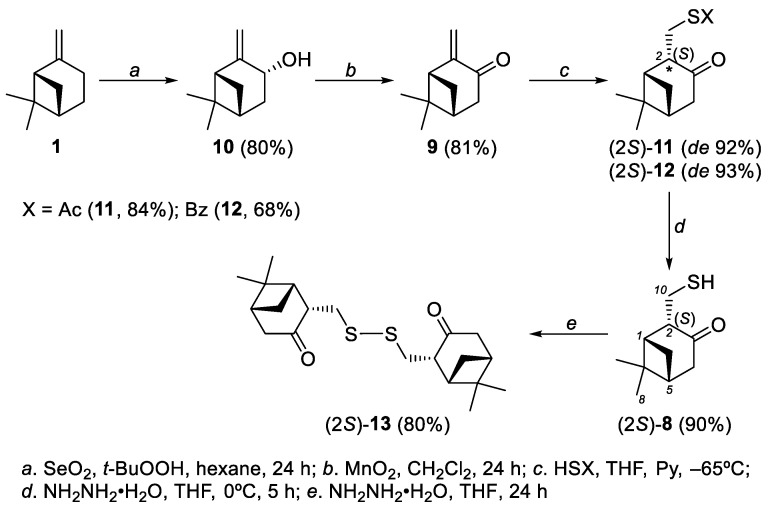
Synthesis of 10-sulfanyl-isopinocamphone derivatives.

Ketothiol (2*S*)-**8** was obtained from pinocarvone **9** according to Scheme 2. The synthesis of pinocarvone **9** was carried out according to the method [13] by sequential oxidation of (−)-β-pinene **1** with *t*-BuOOH to *trans*-pinocarveol **10** in the presence of catalytic amounts of SeO_2_ at room temperature. Then, pinocarveol **10** was oxidized with active MnO_2_ in CH_2_Cl_2_.

Procedure [10] was used to obtain thioacetate (2S)-**11**; however, according to [8], the diastereselectivity (*de*) of the reaction under the described conditions does not exceed 33%. We varied conditions such as the solvent (dichloromethane, THF) and the reagent (thioacetic, thiobenzoic acid) to increase the stereoselectivity of the thia-Michael addition reaction (Table 1).

It was found that if the reaction is carried out at temperature −60–−65 °C in THF in the presence of pyridine, the stereoselectivity of the formation of compounds (2*S*)-**11** and (2*S*)-**12** increases from 33 to 93% *de* (Scheme 2, Table). The increase in the stereoselectivity of the reaction when using THF or CH_2_Cl_2_ as compared to carrying out the reaction without a solvent is apparently explained by a decrease in the concentration of the reactants, as well as by the influence of the nature of the solvent.

Deacylation of thioacetate (2*S*)-**11** to thiol (2*S*)-**8** by NH_2_NH_2_·H_2_O proceeds within 4–5 h with yields up to 90%, while deacylation of thiobenzoate (2*S*)-**12** gives 38–50% yields due to incomplete conversion. An increase in the deacylation time of (2*S*)-**12** to 24 h leads to the formation of the corresponding disulfide (2*S*)-**13** (80%) (Scheme 2).

Thus, at comparable maximum values of *de* of thiocarboxylates (2*S*)-**11**, (2*S*)-**12**, the preparation of thiol (2*S*)-**8** from thioacetate (2*S*)-**11** is more optimal, taking into account the higher total yield of thiol and the time of the deacylation reaction.

The structure and elemental composition of the compounds were confirmed by NMR and IR spectroscopy and the data of elemental analysis. Thus, the signals of C10 carbon atoms in the ^13^C NMR spectra of compounds **5**, (2*S*)-**11,** and (2*S*)-**12** were shifted to a strong field (28.5, 30.1, and 29.9 ppm, respectively) relative to the analogous signals of 2-norpinanone **2** and pinocarvone **9** (122.5 and 117.4 ppm, respectively). The IR spectra of sulfur-containing compounds 5, (2*S*)-**11**, and (2*S*)-**12** contain additional absorption bands in the region of 1702–1712 cm^−1^, corresponding to the S-C=O group.

**Figure 1:**
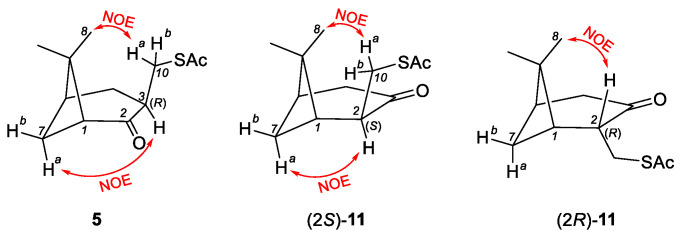
*NOE*-interactions in compounds (3*R*)-**5**, (2*S*)-**11,** and (2*R*)-**11**.

The configuration of the chiral centers of sulfanyl derivatives was proved by the method of two-dimensional NMR spectroscopy. The NOESY spectra contain cross-peaks H7a−H3 for compounds **5**–**7**, cross-peaks H7a−H2 for compounds (2*S*)-**8**, (2*S*)-**11**, (2*S*)-**12**, and (2*S*)-**13,** and cross-peaks between the protons of the methylene H10a and methyl H8 groups for all of the above compounds. Interactions between protons H8–H2 are present in the NOESY spectra of derivatives (2*R*)-**11** and (2*R*)-**12** (Figure 1).

The ratio of derivatives (2*S*)-, (2*R*)-**11** and (2*S*)-, (2*R*)-**12** was determined by ^1^H NMR from the integral intensities of the signals of the corresponding protons H10b.

Thus, thiols **6** and (2*S*)-**8** were synthesized with diastereomeric purities of 98 and 92% *de*, respectively, which were used further to study their oxidation reactions.

### 2.2. Oxidative Transformations of Pinane γ-Ketothiols and Disulfides

Thiols **6** and (2*S*)-**8** were subjected to oxidative transformations using chlorine dioxide (ClO_2_) as a reagent. Chlorine dioxide is produced on an industrial scale and used for pulp bleaching and water disinfection. The presence of an unpaired electron and two reaction centers (chlorine and oxygen) provide this reagent with properties that are different from other oxidizing agents. Its good solubility in water and organic solvents makes it possible to carry out reactions in various media.

Previously, the reactions of ClO_2_ with alkane-, aryl-, hetaryl-, mono-, and diterpene thiols were studied [5,14,15]. It was shown that the main oxidation products are the corresponding disulfides, thiosulfonates, sulfonyl chlorides, and sulfonic acids, and in some cases, trisulfides, ketones, and sulfonic acid esters. The composition of the products is mainly influenced by the structure of the substrate, and the product yields are influenced by the reaction conditions, such as the molar ratio of the reagents, the nature of the solvent, the presence of the catalyst, and the method of mixing the reagents. The reactions of monoterpene ketothiols with ClO_2_ have not been studied previously.

**Scheme 3:**
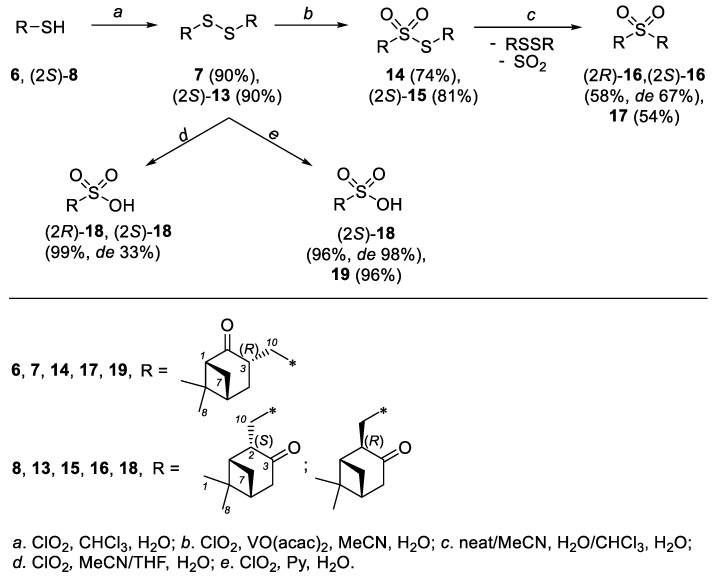
Oxidation of thiols **6** and (2*S*)-**8** with chlorine dioxide.

Depending on the reaction conditions, the main oxidation products of thiols **6** and (2*S*)-**8** are the corresponding disulfides **7** and **13**, thiosulfonates **14** and **15**, sulfones **16** and **17**, and sulfonic acids **18** and **19** (Scheme 3). 

The reactions of thiols **6** and (2*S*)-**8** with ClO_2_ were carried out in hexane, acetonitrile, THF, pyridine, or chloroform in the presence of water or in anhydrous conditions, with or without the VO(acac)_2_ catalyst. The molar ratio of thiol: oxidant was varied in the range 1:1–1:4. The influence of the polarity of the medium on the reaction rate was revealed, which indicates the formation of polar intermediates. Thus, during the oxidation of 1 mol of thiol **6** in hexane with an equimolar amount of ClO_2_ for 0.5 h, the thiol conversion was 22%, while in chloroform, it was complete.

Disulfides **7** and (2*S*)-**13** are formed at the first stage of oxidation of thiols **6** and (2*S*)-**8**. The maximum yields of disulfides are about 90% upon oxidation with an equimolar amount of ClO_2_ in chloroform.

An inseparable mixture of products is formed during the oxidation of disulfides **7** and (2*S*)-**13** by ClO_2_ in more polar solvents (acetonitrile, THF). Therefore, we used vanadyl acetylacetonate catalyst (VO(acac)_2_) to increase the chemoselectivity of the process. We had previously shown its effect on the selectivity of the formation of thiosulfonates and sulfochlorides [14]. The presence of VO(acac)_2_ in the reaction of disulfides **7** and (2*S*)-**13** with an aqueous solution of ClO_2_ in acetonitrile leads to an increase in the yields of thiosulfonates **14** and (2*S*)-**15** from 20% to 74 and 81%, respectively.

**Scheme 4:**
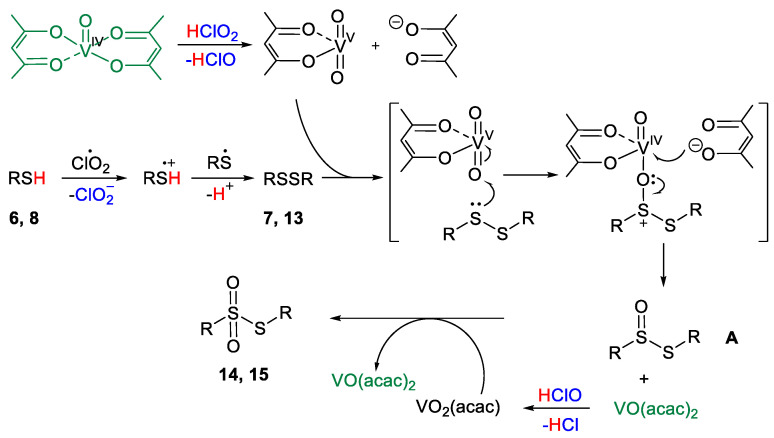
Proposed mechanism of catalytic oxidation of thiols **6** and **8**.

The involvement of the catalyst in the oxidation of thiols with ClO_2_ is shown in Scheme 4. According to the literature [16], a radical cation and a chlorite anion are formed at the first stage of the oxidation of thiols **6** and **8** with ClO_2_. Further, the chlorite anion deprotonates the radical cation, while the RS· radicals recombine to form disulfides **7** and (2*S*)-**13**. 

VO(acac)_2_ is probably oxidized with HClO_2_ to the compound VO_2_(acac) by oxo-transfer mechanism [17]. Then, the disulfide molecules are coordinated on the oxygen atom of VO_2_(acac), oxidized to thiolsulfinates **A**, and the VO_2_(acac) compound is reduced to VO(acac)_2_. The next stage is the catalytic oxidation of thiosulfinates **A** to thiosulfonates **14** and **15**.

Thus, the direct oxidizing agent of disulfides in the catalytic reactions with the participation of VO(acac)_2_ is a vanadium compound in the highest oxidation state +5, presumably VO_2_(acac).

The formation of thiosulfonates **14** and (2*S*)-**15** is evidenced by the downfield shift of the signals of C10 atoms (62.4 and 62.5 ppm, respectively) relative to the analogous signals of disulfides **7** and (2*S*)-**13** (38.4 ppm and 38.9 ppm, respectively). The IR spectra of thiosulfonates **14** and **15** contain absorption bands of the SO_2_ group bonds (1321 and 1128–1130 cm^−1^, respectively) and the C=O group (1708 and 1712 cm^−1^, respectively).

**Scheme 5:**
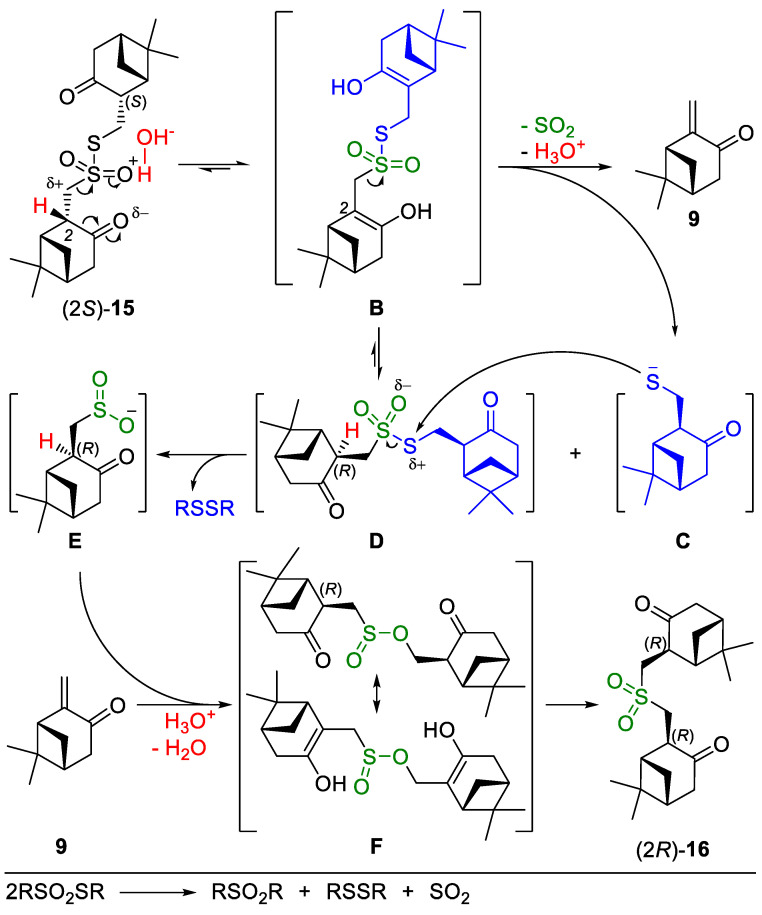
Proposed scheme for the transformation of thiosulfonate (2*S*)-**15** to sulfone (2*R*)-**16**.

The study revealed the transformation of thiosulfonates **14** and (2*S*)-**15** into stable sulfones **16** and **17** and disulfides (Scheme 3). The formation of sulfones in yields up to 58% occurs under the conditions of non-catalytic reactions or during storage at −18–−25 °C for 1–3 weeks. This is probably due to the strong polarization of the SO_2_–S and CH_2_–SO_2_ bonds in thiosulfonates containing the carbonyl group, which is enhanced under the influence of water in the reaction medium or atmospheric moisture, as well as instability of the enol form of thiosulfonate **B** (Scheme 5).

It was found that thiosulfonate (2*S*)-**15** was converted to sulfone (2*R*)-**16**. According to ^1^H NMR data, the ratio of diastereomers (2*S*)-**16**: (2*R*)-**16** is 1:5 (*de* 67%). In accordance with ^13^C NMR spectra, an inseparable mixture of disulfides RSSR with (2*R*)-, (2*S*)-, and (2*R*,2′*S*)-configurations is formed upon the transformation of thiosulfonate (2*S*)-**15** in addition to sulfones (2*S*)-**16** and (2*R*)-**16** (Scheme 5). This was also confirmed by an additional experiment, in which a mixture of supposed disulfides was oxidized with ClO_2_ to sulfonic acids (2*S*)-**18** and (2*R*)-**18**.

The study of the transformation dynamics of thiosulfonate **15** by the NMR method showed that accumulation of pinocarvone **9** occurs at the first stage (Scheme 5), followed by the formation of sulfones (2*S*)-**16** and (2*R*)-**16**. Probably, C–SO_2_ bond cleavage occurs at the stage of enolization of thiosulfonate **B** with the formation of pinocarvone **9**, SO_2_ molecule and sulfenic anion **C**, which nucleophilically attacks the sulfenyl sulfur atom of another thiosulfonate molecule (2*S*)-**15** or **D** with the formation of the disulfide molecule RSSR and sulfinate anion **E**. The addition of the bulky anion **E** and the proton at the double bond of pinocarvone **9** occurs from the sterically accessible side with the probable formation of an unstable ester **F**, which rearranges into sulfone (2*R*)-**16**. According to the literature, a similar rearrangement occurs easily in allylic sulfinic esters [18].

It was found that keto-derivatives based on thiol **6** are sterically stable. Thus, the only diastereomer of sulfone (3*R*)-**17** is formed during the transformation of thiosulfonate (3*R*)-**14**. 

The difference in the stereochemistry of the formation of sulfones **16** and **17** is probably due to both steric and electronic factors. The mobility of the H2 proton in sulfones (2*S*)-, (2*R*)-**16** can be associated with a significant de-shielding of the nucleus of the C2 atom in comparison with the C3 atom in sulfone **17**, as evidenced by the ^13^C NMR spectra. Thus, the values of the chemical shifts of C2 carbon atoms in sulfones (2*S*)-, (2*R*)-**16** are significantly shifted downfield (50.1 and 46.6 ppm, respectively) relative to the analogous signal of the C3 carbon atom in sulfone **17** (37.1 ppm).

The structures of sulfones **16–17** were proved by NMR, IR spectroscopy, and mass spectrometry and confirmed by the data of elemental analysis. In the ^13^C NMR spectra, the signals of C10 atoms of sulfones (2*S*)-, (2*R*)-**16**, and **17** (54.8, 55.8, and 53.9 ppm, respectively) are shifted upfield relative to the analogous signals of carbon atoms of thiosulfonates **14** and **15** (62.4 and 62.5 ppm, respectively). The IR spectra of sulfones **16**–**17** contain absorption bands of SO_2_ groups corresponding to stretching asymmetric and symmetric vibrations (1138 and 1292, 1323 cm^−1^) and C=O groups (1708–1714 cm^−1^). The mass spectra of compounds **16**–**17** exhibit the corresponding molecular peaks 389.47 (100) [M+Na]^+^ and 367.29 (83) [M+H]^+^.

The two-dimensional NOESY spectroscopy proved the configuration of the C2-chiral centers of sulfones (2*S*)-, (2*R*)-**16** by the presence of cross-peaks H10a–H8 and H7a–H2 in the spectrum of sulfone (2*S*)-**16** and a cross-peak H8–H2 in the spectrum of sulfone (2*R*)-**16** (see Supplementary Materials, Figures S1 and S2).

**Scheme 6:**
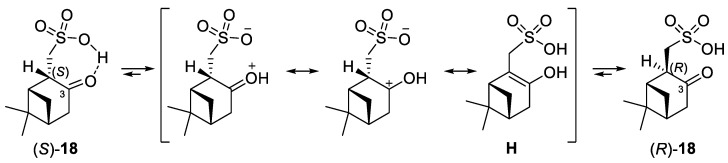
Proposed scheme for the isomerization of sulfonic acid (2*S*)-**18** to acid (2*R*)-**18**.

Oxidation of thiol (2*S*)-**8** or disulfide (2*S*)-**13** with a twofold excess of ClO_2_ in the acetonitrile–water medium in the presence of VO(acac)_2_ catalyst or without it leads to a mixture of (2*S*)-**18** and (2*R*)-**18** sulfonic acids in the ratio 1:2, respectively (*de* 33%) (see Supplementary Materials, Figure S3). Probably, acid autocatalysis of the enolization process with the formation of intermediate **H** is the cause of epimerization in this case (Scheme 6).

The acidic nature of the epimerization of acid (2*S*)-**18** is confirmed by a similar reaction in an aqueous solution of pyridine. Under these conditions, acid (2*S*)-**18** is quantitatively formed (*de* 98%). Pyridine, being basic in nature, binds the mobile proton of the SO_3_H-group and prevents the enolization process. 

No epimerization of acid **19** was detected in the reactions of thiol **6** with ClO_2_ under various conditions. The quantitative formation of acid **19** was observed upon oxidation with an aqueous solution of ClO_2_ in pyridine.

The signals of the C10 atoms of the sulfonic acids (2*S*)-**18**, (2*R*)-**18**, and **19** (51.0, 52.9, and 50.7 ppm, respectively) are shifted downfield relative to the analogous signals of the carbon atoms of disulfides **7** and (2*S*)-**13** (38.4 ppm and 38.9 ppm, respectively). The IR spectra of sulfonic acids (2*S*)-**18**, (2*R*)-**18**, and **19** contain absorption bands of SO_2_ groups corresponding to stretching asymmetric and symmetric vibrations (1035–1039 and 1161–1242 cm^−1^), C=O groups (1705–1714 cm^−1^), and OH groups (3327–3439 cm^−1^). In the mass spectra of compounds (2*S*)-**18**, (2*R*)-**18**, and **19,** the corresponding molecular peaks with an intensity of 100% are observed.

The two-dimensional NOESY spectroscopy proved the configuration of the chiral centers at the C2 atom of acids (2*S*)-**18** and (2*R*)-**18** by the presence of the cross-peaks H10a–H8 and H7a–H2 in the spectra of acid (2*S*)-**18** and the cross-peak H8–H2 in the spectra of acid (2*R*)-**18** (see Supplementary Materials, Figures S4 and S5). The ratios of the acids (2*S*)-**18** and (2*R*)-**18** were determined by ^1^H NMR spectroscopy from the values of the integral intensities of the signals of the corresponding H10b protons.

## 3. Conclusions

The new chiral 10-sulfanyl- and 10-sulfonyl derivatives were obtained from β-pinene through α-, β-unsaturated ketones: γ-ketothioacetates, -thiobenzoate, -thiols, -disulfides, -sulfones, -thiosulfonates, and -sulfonic acids. It was shown that the syntheses of 10-sulfanyl- and 10-sulfonyl derivatives based on 2-norpinanone are characterized by high stereoselectivity (*de* 98%) with respect to (*R*)-derivatives at the C3 atom as compared to the reactions of pinocarvone. This is probably due to the influence of steric factors, which determine the coordination of reagents predominantly on one side of the molecule. The conditions for the synthesis of diastereomerically enriched thioacetyl and thiobenzoyl derivatives (*de* 93%) based on pinocarvone with yields up to 84% were selected.

The chemo- and stereoselectivity of the reactions of terpene ketothiols with chlorine dioxide were investigated depending on the reaction conditions and the structure of the substrate. It was shown that the catalytic oxidation of disulfides in the presence of VO(acac)_2_ increases the yields of thiosulfonate from 20 to 74–81%. The effect of basic pyridine on the stereoselectivity with respect to (2*S*)-3-ketosulfonic acid, as well as on the chemoselectivity of the formation of 2- and 3-ketosulfonic acids, was shown. A new direction of the transformation of thiosulfonates with the formation of sulfones was revealed, while the 3-ketosulfone undergoes a configuration inversion at the C2 atom. An epimerization scheme was proposed.

## 4. Materials and Methods

### 4.1. General Information

FT-IR spectra were recorded on Shimadzu IR Prestige 21 on thin films. ^1^H- and ^13^C-NMR spectra were registered on Bruker Avance 300 spectrometer (300.17 MHz for ^1^H, 75.48 MHz for ^13^C) in CDCl_3_ or DMSO-*d^6^* solutions. Complete assignment of ^1^H and ^13^C signals was performed using two-dimensional homo- (^1^H–^1^H COSY, ^1^H–^1^H NOESY) and heteronuclear experiments (^1^H–^13^C HSQC, HMBC). The ratio of (*S*)- and (*R*)-derivatives was determined by ^1^H NMR spectroscopy from the values of integral intensities of the signals of the corresponding H10b protons. Thin-layer chromatography (TLC) was performed on Sorbfil plates; spots were visualized by treatment with 5% vanillin in EtOH. Mass spectra were recorded on a high-performance liquid chromatograph with mass selective detector Thermo Finnigan LCQ Fleet (USA) (solvents—H_2_O, CH_3_OH, CH_3_CN, direct input). Detection was carried out using negative and positive ions. Automatic analyzer EA 1110 CHNS-O was employed for elemental analysis. All reactions were carried out using freshly distilled solvents. Silica gel 60 (70–230 mesh, Alfa Aesar) was used for column chromatography (CC). The melting points were measured on Sanyo Gallenkamp MPD350.BM3.5 and were not corrected. Optical rotations were measured with automatized digital polarimeter Optical Activity PolAAr 3001.

(−)-β-Pinene (**1**) is a commercial product from Sigma Aldrich, 99% purity, ${\left[\alpha \right]}_{D}^{25}=$ −22 (neat). Hydrazine hydrate is commercially available from Alfa Aesar, 98% purity.

An aqueous solution of ClO_2_ is a product manufactured by Mondi Syktyvkar JSC. The concentration of the solution was determined by titration according to a certain method [19]. An organic ClO_2_ solution was obtained by extraction of an aqueous solution, dried with Na_2_SO_4_, and titrated to establish the concentration.

2-Norpinanone **2** (${\left[\alpha \right]}_{D}^{25}=$ +25.4 (*c* 0.5, CHCl_3_)), nopinone **4** (${\left[\alpha \right]}_{D}^{25}=$ +32.2 (*c* 0.9, EtOH)), pinocarvone **9** (${\left[\alpha \right]}_{D}^{25}=$ +64.5 (*c* 1.0, EtOH)), and *trans-*pinocarveol **10** (${\left[\alpha \right]}_{D}^{25}=$ +72.4 (*c* 0.9, EtOH)) were synthesized according to certain methods [9,13], and the physicochemical characteristics correspond to the data in literature.

### 4.2. General Procedure

*(1R,5R,E)-3-(Hydroxymethylene)-6,6-dimethylbicyclo [3.1.1]heptan-2-one (**3**). t*-BuOK (15.38 mmol, 1.726 g) was added to a solution of (−)-nopinone **4** (11.4 mmol, 1.575 g) in 20 mL THF cooled to 0 °C. After the dissolution of *t*-BuOK, a solution of isoamyl formate (15.38 mmol, 1.726 g) in 10 mL of THF was added dropwise to the mixture. The resulting mixture was stirred for 6 h at room temperature. THF was distilled off, 30 mL of water was added to the dry residue and acidified to pH = 6–7 with 10% HCl solution, and the mixture was extracted with Et_2_O (3 × 20 mL). The combined organic layers were washed with brine, dried over Na_2_SO_4_, and the solvent was distilled off to obtain 1.818 g of ketoenol **3** (yield 96%). Subsequently, compound **3** was used without additional purification. Physicochemical characteristics correspond to the data in the literature [9].

*S-(((1R,3R,5R)-6,6-Dimethyl-2-oxobicyclo[3.1.1]heptan-3-yl)methyl) ethanethioate (**5**).* Two drops of pyridine (~1 mol%) and 3.0 g (0.04 mol) of thioacetic acid were added to 3.0 g (0.02 mol) of 2-norpinanone **2** with stirring. The synthesis time was 5–10 min. The reaction progress was monitored by TLC (eluent–CH_2_Cl_2_, developer–vanillin solution in EtOH). After completion of the reaction, a saturated solution of NaHCO_3_ was added, and the mixture was extracted with CH_2_Cl_2_ (3 × 20 mL). The combined organic layers were dried over Na_2_SO_4_, and the solvent was removed under reduced pressure. The product was purified by column chromatography (eluent–CH_2_Cl_2_). Light-yellow liquid, 79% yield (3.6 g), 98% *de*,$\;{\left[\alpha \right]}_{D}^{25}=$+22.4 (*c* 0.35, CHCl_3_). ^1^H NMR spectrum (CDCl_3_, δ, ppm, *J*/Hz): 0.77 (s, 3H, H*^8^*), 1.35 (s, 3H, H*^9^*), 1.61 (dd, 1H, H*^4a^*, *J* = 13.1, 8.1), 1.72 (d, 1H, H*^7a^*, *J* = 10.5), 2.23–2.31 (m, 1H, H*^5^*), 2.36 (s, 3H, CH_3_*^Ac^*), 2.38–2.53 (m, 2H, H*^4β^*, H*^7b^*), 2.63 (t, 1H, H*^1^*, *J* = 5.1), 2.78–2.92 (m, 1H, H*^3^*), 3.00 (dd, 1H, H*^10a^*, *J* = 13.3, 7.84), 3.41 (dd, 1H, H*^10b^*, *J* = 13.5, 5.5). ^13^C NMR spectrum (CDCl_3_, δ, ppm): 21.9 (C*^8^*), 25.2 (C*^7^*), 26.3 (C*^9^*), 28.2 (C*^4^*), 28.5 (C*^10^*), 30.5 (C*^Ac^*), 40.7 (C*^5^*), 42.8 (C*^3^*), 43.4 (C*^6^*), 57.5 (C*^1^*), 195.8 (C*^2^*), 213.8 (C*^Ac^* = O). IR spectrum (KBr, ν, cm^−1^): 628, 954, 1024, 1109, 1136 st (C–O), 1199, 1253, 1352, 1419, 1460, 1693 st (C=O), 1708 st (C=O), 2872 st, 2927 st; elemental analysis calcd (%) for C_12_H_18_O_2_S: C 63.68, H 8.02, S 14.16; found: C 63.65, H 7.96, S 14.20.

*(1R,3R,5R)-3-(Thiomethyl)-6,6-dimethylbicyclo[3.1.1]heptan-2-one (**6**)***.** Hydrazine hydrate 0.1 g (2 mmol) was added dropwise to a solution of 0.23 g (1 mmol) of thioacetate **5** in 2.5 mL of THF cooled to 0 °C. The mixture was stirred for 4 h. 1N HCl solution (2 mmol) was added to the reaction mixture to wash from hydrazine hydrate. The mixture was extracted with Et_2_O (3 × 20 mL) and washed with brine. The extract was dried over Na_2_SO_4_, and the solvent was removed under reduced pressure. The product was purified by column chromatography (eluent–CHCl_3_). Transparent liquid, 75% yield (0.14 g), ${\left[\alpha \right]}_{D}^{25}=$ +2.2 (*c* 0.27, CHCl_3_). ^1^H NMR spectrum (CDCl_3_, δ, ppm, *J*/Hz): 0.75 (s, 3H, H*^8^*), 1.33 (s, 3H, H*^9^*), 1.56–1.62 (m, 1H, H*^4a^*), 1.65 (t, 1H, SH, *J* = 8.6), 1.71 (d, 1H, H*^7a^*, *J* = 9.9), 2.25–2.34 (m, 1H, H*^5^*), 2.40–2.54 (m, 3H, H*^4b^*, H*^7b^*, H*^10a^*), 2.54–2.62 (m, 1H, H*^1^*), 2.72–2.86 (m, 1H, H*^3^*), 3.15 (ddd, 1H, H*^10b^*, *J* = 13.5, 8.9, 4.6). ^13^C NMR spectrum (CDCl_3_, δ, ppm): 21.9 (C*^8^*), 24.4 (C*^10^*), 25.4 (C*^7^*), 26.3 (C*^9^*), 28.0 (C*^4^*), 40.7 (C*^5^*), 43.2 (C*^6^*), 45.5 (C*^3^*), 57.5 (C*^1^*), 214.1 (C*^2^*). IR spectrum (KBr, ν, cm^−1^): 472, 534, 628, 954, 1026, 1087, 1172, 1199, 1253, 1315, 1346, 1369, 1386, 1458, 1707 st (C=O), 2567 (SH), 2870 st, 2931 st; elemental analysis calcd (%) for C_10_H_16_OS: C 65.17, H 8.75, S 17.40; found: C 65.35, H 8.76, S 17.49.

*(1R,1′R,3R,3′R,5R,5′R)-3,3′-(Disulfanediylbis(methylene))bis(6,6-dimethylbicyclo[3.1.1]heptan-2-one) (**7**).* An aqueous solution containing 0.068 g (1 mmol) of ClO_2_ was added to a solution of 0.184 g (1 mmol) of thiol **6** in 20 mL of chloroform (the volume of the aqueous solution varied depending on the ClO_2_ concentration; it did not affect the composition and ratio of products). The solvent was distilled off after 30 min. The residue was purified by column chromatography (eluent –CHCl_3_). White powder, m.p. 97–99 °C, 90% yield (0.150 g), ${\left[\alpha \right]}_{D}^{25}=$ −174.4 (*c* 0.51, CHCl_3_). ^1^H NMR spectrum (CDCl_3_, δ, ppm, *J*/Hz): 0.72 (s, 3H, H*^8^*), 1.32 (s, 3H, H*^9^*), 1.58 (dd, 1H, H*^4a^*), 1.74 (d, 1H, H*^7a^*, *J* = 10.6), 2.23–2.32 (m, 1H, H*^5^*), 2.40–2.53 (m, 3H, H*^4b^*, H*^7b^*, H*^10a^*), 2.60 (t, 1H, H*^1^*, *J* = 5.28), 2.94–3.07 (m, 1H, H*^3^*), 3.52 (dd, 1H, H*^10b^*, *J* = 13.2, 3.9). ^13^C NMR spectrum (CDCl_3_, δ, ppm): 22.0 (C*^8^*), 25.3 (C*^7^*), 26.2 (C*^9^*), 28.6 (C*^4^*), 38.4 (C*^10^*), 40.7 (C*^5^*), 41.7 (C*^3^*), 42.9 (C*^6^*), 57.6 (C*^1^*), 214.3 (C*^2^*). IR spectrum (KBr, ν, cm^−1^): 472, 536, 1024, 1197, 1253, 1311, 1705 st (C=O), 2868 st, 2941 st; elemental analysis calcd (%) for C_20_H_30_O_2_S_2_: C 65.53, H 8.25, S 17.49; found: C 66.05, H 8.36, S 17.30.

*(1S,2S,5R)-2-(Thiomethyl)-6,6-dimethylbicyclo[3.1.1]heptan-3-one ((2S)-**8**)* was synthesized similarly to thiol **6** from thioacetate (2*S*)-**11**. The product was purified by column chromatography (eluent–CH_2_Cl_2_: Et_2_O = 30:1). Transparent liquid, 90% yield, ${\left[\alpha \right]}_{D}^{25}=$ +41.8 (*c* 0.22, CHCl_3_). ^1^H NMR spectrum (CDCl_3_, δ, ppm, *J*/Hz): 0.83 (s, 3H, H*^8^*), 1.19 (d, 1H, H*^7a^*, *J* = 10.8), 1.33 (s, 3H, H*^9^*), 1.56 (t, 1H, SH, *J* = 8.21), 2.09–2.18 (m, 1H, H*^5^*), 2.31–2.41 (m, 1H, H*^10a^*), 2.44–2.52 (m, 2H, H*^1^*, H*^2^*), 2.54–2.29 (m, 1H, H*^4a^*), 2.60–2.74 (m, 1H, H*^4b^*, H*^7b^*), 3.27–3.38 (m, 1H, H*^10b^*). ^13^C NMR spectrum (CDCl_3_, δ, ppm): 21.7 (C*^8^*), 25.6 (C*^10^*), 26.7 (C*^9^*), 33.8 (C*^7^*), 38.5 (C*^5^*), 39.0 (C*^6^*), 40.6 (C*^1^*), 44.5 (C*^4^*), 60.2 (C*^2^*), 211.6 (C*^3^*). IR spectrum (KBr, ν, cm^−1^): 486, 734, 952, 1047, 1080, 1159, 1201, 1271, 1323, 1409, 1467, 1710 st (C=O), 2567 (SH), 2877 st, 2923 st, 2970 st; elemental analysis calcd (%) for C_10_H_16_OS: C 65.17, H 8.75, S 17.40; found: C 65.65, H 8.76, S 17.49.

*S-(((1S,2S,5R)-6,6-Dimethyl-3-oxobicyclo[3.1.1]heptan-2-yl)methyl) ethanethioate ((2S)-**11**).* A solution of 3.0 g (0.02 mol) of pinocarvone **9** in 18 mL of THF was cooled with stirring to −65 °C, two drops of pyridine (~1 mol%) were added, and then a solution of 3.0 g (0.04 mol) of thioacetic acid was added in 18 mL THF. The progress of the reaction was monitored by TLC (eluent–petroleum ether: EtOAc = 5:1, developer–vanillin solution in EtOH). The mixture was stirred for 6h, then a saturated NaHCO_3_ solution was added, and the mixture was extracted with Et_2_O (3 × 20 mL). The combined organic layers were dried over Na_2_SO_4_, and the solvent was removed under reduced pressure. The product was purified by column chromatography (eluent–CH_2_Cl_2_: Et_2_O = 30:1). Light-yellow liquid, 84% yield (3.8g), 92% *de*, ${\left[\alpha \right]}_{D}^{26}=$ −9.6 (*c* 0.25, CHCl_3_). ^1^H NMR spectrum (CDCl_3_, δ, ppm, *J*/Hz): 0.95 (s, 3H, H*^8^*), 1.17 (д, 1H, H*^7a^*, *J* = 10.3), 1.33 (c, 3H, H*^9^*), 2.11–2.18 (m, 1H, H*^5^*), 2.22–2.30 (m, 1H, H*^1^*), 2.34 (s, 3H, H*^12^*), 2.46–2.60 (m, 2H, H*^2^*, H*^4a^*), 2.64–2.73 (m, 2H, H*^4b^*, H*^7b^*), 2.96 (dd, 1H, H*^10a^*, *J* = 13.9, 9.8), 3.47 (dd, 1H, H*^10b^*, *J* = 13.2, 4.6). ^13^C NMR spectrum (CDCl_3_, δ, ppm): 21.7 (C*^8^*), 26.7 (C*^9^*), 30.1 (C*^10^*), 30.4 (C*^12^*), 33.9 (C*^7^*), 38.6 (C*^5^*), 39.1 (C*^6^*), 41.6 (C*^1^*), 44.6 (C*^4^*), 56.6 (C*^2^*), 195.3 (C*^11^*), 211.5 (C*^3^*). IR spectrum (KBr, ν, cm^−1^): 628, 756, 822, 957, 1047, 1134 st, 1199, 1325, 1411, 1469, 1691 st (C=O), 1712 st (C=O), 2877 st, 2929 st, 2968 st; elemental analysis calcd (%) for C_12_H_18_O_2_S: C 63.68, H 8.02, S 14.16; found: C 63.65, H 8.16, S 14.20.

*S-(((1S,2R,5R)-6,6-Dimethyl-3-oxobicyclo[3.1.1]heptan-2-yl)methyl) ethanethioate ((2R)-**11**)* was synthesized according to the method[6] from pinocarvone **9** upon cooling to –50 °C. The product was purified by column chromatography (eluent–CH_2_Cl_2_: Et_2_O = 30:1). The ratio was (2*S*)-**11**: (2*R*)-**11** ≈ 5:1 (*de* 69%). The yield of the mixture (2*S*)-**11**, (2*R*)-**11** was 83%. NMR spectra were obtained by subtracting signals from the spectrum of the mixture of thioacetates (2*S*)*-***11**, (2*R*)*-***11**. ^1^H NMR spectrum (CDCl_3_, δ, ppm, *J*/Hz): 0.88 (s, 3H, H*^8^*), 1.26–1.35 (m, 4H, H*^7a^*, H*^9^*), 2.11–2.18 (m, 1H, H*^5^*), 2.22–2.72 (m, 8H, H*^1^*, H*^2^*, H*^4^*, H*^7b^*, H*^12^*), 2.90–3.00 (m, 1H, H*^10a^*), 3.30–3.38 (m, 1H, H*^10b^*). ^13^C NMR spectrum (CDCl_3_, δ, ppm): 19.7 (C*^8^*), 26.3 (C*^9^*), 28.7 (C*^10^*), 29.1 (C*^7^*), 30.4 (C*^12^*), 38.0 (C*^5^*), 38.3 (C*^6^*), 41.2 (C*^1^*), 44.2 (C*^4^*), 51.7 (C*^2^*), 195.3 (C*^11^*), 211.5 (C*^3^*). Elemental analysis calcd (%) for C_12_H_18_O_2_S: C 63.68, H 8.02, S 14.16; found: C 63.65, H 8.16, S 14.20.

*S-(((1S,2S,5R)-6,6-Dimethyl-3-oxobicyclo[3.1.1]heptan-2-yl)methyl) benzothioate ((2S)-**12**)* was obtained by the reaction of pinocarvone **9** with thiobenzoic acid similarly to thioacetate (2*S*)-**11**. Transparent liquid, 68% yield, *de* 93%, ${\left[\alpha \right]}_{D}^{25}=$+3.5 (*c* 0.23, CHCl_3_). ^1^H NMR spectrum (CDCl_3_, δ, ppm, *J*/Hz): 1.01 (s, 3H, H*^8^*), 1.19 (d, 1H, H*^7a^*, *J* = 10.3), 1.34 (s, 3H, H*^9^*), 2.10–2.19 (m, 1H, H*^5^*), 2.34 (t, 1H, H*^1^*, *J* = 6.2), 2.52–2.78 (m, 4H, H*^2^*, H*^4a^*, H*^4b^*, H*^7b^*), 3.16 (dd, 1H, H*^10a^*, *J* = 13.6, 10.0), 3.69 (dd, 1H, H*^10b^*, *J* = 13.9, 4.7), 7.45 (t, 2H, H*^Ar^*, *J* = 7.7), 7.53–7.62 (m, 1H, H*^Ar^*), 7.98 (d, 2H, H*^Ar^*, *J* = 8.7). ^13^C NMR spectrum (CDCl_3_, δ, ppm): 21.7 (C*^8^*), 26.7 (C*^9^*), 29.9 (C*^10^*), 33.9 (C*^7^*), 38.6 (C*^5^*), 39.1 (C*^6^*), 41.7 (C*^1^*), 44.7 (C*^4^*), 56.7 (C*^2^*), 127.1 (C*^Ar^*), 128.5 (C*^Ar^*), 133.3 (C*^Ar^*), 136.8 (C*^Ar^*), 191.4 (C*^Ar^*), 211.5 (C*^3^*). Elemental analysis calcd (%) for C_17_H_20_O_2_S: C 70.80, H 6.99, S 11.12; found: C 70.65, H 7.06, S 11.20.

*(1S,1′S,2S,2′S,5R,5′R)-2,2′-(Disulfanediylbis(methylene))bis(6,6-dimethylbicyclo[3.1.1]heptan-3-one) ((2S)-**13**)* was obtained similarly to disulfide **7** from thiol (2*S*)-**8**. White powder, m.p. 93–95 °C, 90% yield, ${\left[\alpha \right]}_{D}^{25}=$+202.6 (*c* 0.23, CHCl_3_). ^1^H NMR spectrum (CDCl_3_, δ, ppm, *J*/Hz): 0.88 (s, 3H, H*^8^*), 1.26 (d, 1H, H*^7a^*, *J* = 10.6), 1.37 (s, 3H, H*^9^*), 2.18 (dd, 1H, H*^5^*, *J* = 5.9, 2.8), 2.42–2.58 (m, 2H, H*^1^*, H*^10a^*), 2.58–2.82 (m, 4H, H*^2^*, H*^4^*, H*^7b^*), 3.53 (dd, 1H, H*^10b^*, *J* = 13.3, 3.6). ^13^C NMR spectrum (CDCl_3_, δ, ppm): 21.9 (C*^8^*), 26.7 (C*^9^*), 33.9 (C*^7^*), 38.7 (C*^5^*), 38.9 (C*^10^*), 39.1 (C*^6^*), 40.9 (C*^1^*), 44.6 (C*^4^*), 55.7 (C*^2^*), 212.1 (C*^3^*). IR spectrum (KBr, ν, cm^−1^): 470, 489, 1047, 1078, 1157, 1999, 1321, 1409, 1467, 1710 st (C=O), 2883 st, 2924 st, 2972 st; elemental analysis calcd (%) for C_20_H_30_O_2_S_2_: C 65.53, H 8.25, S 17.49; found: C 65.85, H 8.30, S 17.40.

*S-(((1R,3R,5R)-6,6-Dimethyl-2-oxobicyclo[3.1.1]heptan-3-yl)methyl) ((1R,3R,5R)-6,6-dimethyl-2-oxobicyclo[3.1.1]heptan-3-yl)methanesulfonothioate (**14**)*. 0.027 g (0.1 mmol) of VO(acac)_2_ was added to a solution of 0.366 g (1 mmol) of disulfide **7** in 40 mL of acetonitrile with stirring, then an aqueous solution of 0.068 g (1 mmol) of ClO_2_ was added dropwise. After 1 h, the reaction mixture was partially evaporated until a precipitate formed (target product). The precipitate was filtered off. Light-yellow waxy powder, 74% yield (0.295 g), ${\left[\alpha \right]}_{D}^{24}=$ +0.1 (c 0.26, CHCl_3_). ^1^H NMR spectrum (CDCl_3_, δ, ppm, J/Hz): 0.74, 0.76 (2s, 6H, H^8^, H^8′^), 1.37 (s, 6H, H^9′^, H^9^), 1.73–1.88 (m, 4H, H^4a^, H^4′a^, H^7a^, H^7′a^), 2.27–2.36 (m, 2H, H^5^, H^5′^), 2.53–2.77 (m, 6H, H^1^, H^1′^, H^4b^, H^4′b^, H^7b^, H^7′b^), 3.05–3.29 (m, 4H, H^3^, H^3′^, H^10a^, H^10′a^), 3.58–3.70 (m, 1H, H^10′b^), 4.32 (d, 1H, H^10b^, J = 11.9). ^13^C NMR spectrum (CDCl_3_, δ, ppm): 22.0, 22.1 (C^8^, C^8′^), 25.1, 25.2 (C^7^, C^7′^), 26.1, 26.3 (C^9′^, C^9^), 28.4 (C^4′^), 29.9 (C^4^), 35.6 (C^10′^), 38.6 (C^3^), 40.6, 40.7 (C^5^, C^5′^), 43.4 (C^3′^), 43.8 (C^6^, C^6′^), 57.2, 57.4 (C^1^, C^1′^), 62.4 (C^10^), 211.1, 213.4 (C^2^, C^2′^). IR spectrum (KBr, ν, cm^−1^): 472, 489, 534, 611, 1024, 1130 st (SO_2_), 1321 st (SO_2_), 1460, 1708 st (C=O), 2872 st, 2945 st; elemental analysis calcd (%) for C_20_H_30_O_4_S_2_: C 60.27, H 7.59, S 16.09; found: C 60.25, H 7.66, S 16.20.

*S-(((1S,2S,5R)-6,6-Dimethyl-3-oxobicyclo[3.1.1]heptan-2-yl)methyl) ((1S,2S,5R)-6,6-dimethyl-3-oxobicyclo[3.1.1]heptan-2-yl)methanesulfonothioate ((2S)-**15**)* was obtained similarly to thiosulfonate **14** from disulfide (2S)-**13**. The ratio (2S)-**13**: ClO_2_ = 1:2. The synthesis time was 1h. Light-yellow powder, m.p. 134–137 °C, 81% yield, ${\left[\alpha \right]}_{D}^{25}=$+9.2 (c 0.24, CHCl_3_). ^1^H NMR spectrum (CDCl_3_, δ, ppm, J/Hz): 0.88, 0.90 (2s, 6H, H^8^, H^8′^), 1.26–1.32 (m, 1H, H^7a^, H^7′a^), 1.35 (s, 6H, H^9′^, H^9^), 2.13–2.21 (m, 2H, H^5^, H^5′^), 2.41 (t, 1H, H^1′^, J = 6.3), 2.51–2.79 (m, 7H, H^1^, H^4^, H^4′^, H^7b^, H^7′b^), 2.82–2.91 (m, 1H, H^2′^), 2.98 (m, 2H, H^2^, H^10′a^), 3.13–3.23 (m, 1H, H^10a^), 3.80 (dd, 1H, H^10′b^, J = 13.6, 5.6), 4.10 (d, 1H, H^10b^, J = 13.9). ^13^C NMR spectrum (CDCl_3_, δ, ppm): 21.8, 21.9 (C^8^, C^8′^), 26.4, 26.6 (C^9′^, C^9^), 33.7, 34.1 (C^7^, C^7′^), 37.2 (C^10′^), 38.4, 38.7 (C^5^, C^5′^), 38.8, 39.2 (C^6^, C^6′^), 42.3, 42.7 (C^1^, C^1′^), 44.5, 44.6 (C^4^, C^4′^), 51.4 (C^2^), 56.4 (C^2′^), 62.5 (C^10^), 209.5, 212.3 (C^3^, C^3′^). IR spectrum (KBr, ν, cm^−1^): 474, 545, 1045, 1128 st (SO_2_), 1321 st (SO_2_), 1409, 1467, 1712 st (C=O), 2872 st, 2924 st, 2933 st; elemental analysis calcd (%) for C_20_H_30_O_4_S_2_: C 60.27, H 7.59, S 16.09; found: C 60.55, H 7.66, S 16.10.

*(1S,1′S,2S,2′S,5R,5′R)-2,2′-(Sulfonylbis(methylene))bis(6,6-dimethylbicyclo[3.1.1]heptan-3-one) ((2S)-**16**)*. An aqueous solution containing 0.135 g (2 mmol) of ClO_2_ was added to a solution of 0.184 g (1 mmol) of thiol (2*S*)-**8** in 20 mL of acetonitrile with stirring. After 30 min, it was extracted with chloroform (3 × 20 mL), and the organic fraction was evaporated. The product was purified by column chromatography (eluent—petroleum ether: Et_2_O = 3:1). The products were obtained in a mixture (2*S*)-**16**: (2*R*)-**16** = 1:5 (*de* 67%). NMR spectra were obtained by subtracting signals from the spectrum of a mixture of sulfones (2*S*)*-***16**, (2*R*)*-***16**. ^1^H NMR spectrum (CDCl_3_, δ, ppm, *J*/Hz): 0.84 (s, 3H, H*^8^*), 1.32–1.36 (m, 4H, H*^7a^*, H*^9^*), 2.12–2.19 (m, 1H, H*^5^*), 2.42–2.59 (m, 4H, H*^1^*, H*^4a^*, H*^4b^*, H*^7b^*), 2.85–2.92 (m, 1H, H*^10a^*), 3.01–3.13 (m, 1H, H*^2^*), 3.77–3.86 (m, 1H, H*^10b^*). ^13^C NMR spectrum (CDCl_3_, δ, ppm): 21.9 (C*^8^*), 26.5 (C*^9^*), 33.8 (C*^7^*), 38.5 (C*^5^*), 38.8 (C*^6^*), 43.4 (C*^1^*), 44.5 (C*^4^*), 50.1 (C*^2^*), 55.8 (C*^10^*), 210.1 (C*^3^*). IR spectrum (KBr, ν, cm^−1^): 459, 481, 763, 1049, 1138 st (SO_2_), 1201, 1292 st (SO_2_), 1321, 1411, 1467, 1714 st (C=O), 2877 st, 2926 st, 2981 st; MS (ESI, 5 kV): *m/z* (%): 389.47 (100) [M+23]^+^, 367.29 (83) [M+1]^+^; elemental analysis calcd (%) for C_20_H_30_O_4_S: C 65.54, H 8.25, S 8.75; found: C 65.65, H 8.30, S 8.69.

*(1S,1′S,2R,2′R,5R,5′R)-2,2′-(Sulfonylbis(methylene))bis(6,6-dimethylbicyclo[3.1.1]heptan-3-one) ((2R)-**16**)* was obtained similarly to sulfone (2*S*)-**16** or during storage of (*S*)-thiosulfonate (2*S*)-**15** for 1–3 weeks. White powder (recrystallized from EtOH), m.p. 168–171 °C, 58% yield, ${\left[\alpha \right]}_{D}^{25}=$ −31.0 (*c* 0.2, CHCl_3_). ^1^H NMR spectrum (CDCl_3_, δ, ppm, *J*/Hz): 1.01 (s, 3H, H*^8^*), 1.14 (d, 1H, H*^7a^*, *J* = 11.2), 1.40 (s, 3H, H*^9^*), 2.15–2.24 (m, 1H, H*^5^*), 2.47–2.66 (m, 3H, H*^1^*, H*^4a^*, H*^7b^*), 2.71–2.84 (m, 1H, H*^4b^*), 3.01 (dd, 1H, H*^10a^*, *J* = 14.1, 9.9), 3.29 (d, 1H, H*^2^*, *J* = 9.9), 3.63 (dd, 1H, H*^10b^*, *J* = 14.1, 2.4). ^13^C NMR spectrum (CDCl_3_, δ, ppm): 19.8 (C*^8^*), 26.2 (C*^9^*), 29.0 (C*^7^*), 37.8 (C*^5^*), 39.3 (C*^6^*), 41.1 (C*^1^*), 44.2 (C*^4^*), 46.7 (C*^2^*), 53.9 (C*^10^*), 210.3 (C*^3^*). IR spectrum (KBr, ν, cm^−1^): 459, 481, 763, 1049, 1138 st (SO_2_), 1201, 1292 st (SO_2_), 1321, 1411, 1467, 1714 st (C=O), 2877 st, 2926 st, 2981 st; MS (ESI, 5 kV): *m/z* (%): 389.47 (100) [M+23]^+^, 367.29 (83) [M+1]^+^; elemental analysis calcd (%) for C_20_H_30_O_4_S: C 65.54, H 8.25, S 8.75; found: C 65.65, H 8.30, S 8.79.

*(1R,1′R,3R,3′R,5R,5′R)-3,3′-(Sulfonylbis(methylene))bis(6,6-dimethylbicyclo[3.1.1]heptan-2-one) (**17**)* was synthesized similarly to sulfone (2*S*)-**16** from thiol **6** or by storage of (*S*)-thiosulfonate **14** for 1–3 weeks. Product was isolated by column chromatography (petroleum ether: Et_2_O = 7:1). White powder (recrystallized from EtOH), m.p. 148–150 °C, 54% yield (0.099 g), ${\left[\alpha \right]}_{D}^{25}=$ +10.0 (*c* 0.1, CHCl_3_). ^1^H NMR spectrum (CDCl_3_, δ, ppm, *J*/Hz): 0.73 (s, 3H, H*^8^*), 1.36 (s, 3H, H*^9^*), 1.75–1.85 (m, 2H, H*^4a^*, H*^7a^*), 2.23–2.34 (m, 1H, H*^5^*), 2.55 (ddd, 1H, H*^7b^*, *J* = 10.9, 5.9, 5.6), 2.68–2.77 (m, 2H, H*^1^*, H*^4b^*), 2.96 (dd, 1H, H*^10a^*, *J* = 13.8, 9.7), 3.28–3.40 (m, 1H, H*^3^*), 4.01 (dd, 1H, H*^10b^*, *J* = 13.5, 2.9). ^13^C NMR spectrum (CDCl_3_, δ, ppm): 22.1 (C*^8^*), 25.1 (C*^7^*), 26.1 (C*^9^*), 30.0 (C*^4^*), 37.1 (C*^3^*), 40.7 (C*^5^*), 43.3 (C*^6^*), 54.8 (C*^10^*), 57.3 (C*^1^*), 211.6 (C*^2^*). IR spectrum (KBr, ν, cm^−1^): 468, 503, 1024, 1136 m (SO_2_), 1201, 1282 m (SO_2_), 1303, 1319, 1462, 1708 st (C=O), 2873 m, 2926 st, 2953 st; elemental analysis calcd (%) for C_20_H_30_O_4_S: C 65.54, H 8.25, S 8.75; found: C 65.65, H 8.30, S 8.80.

*((1S,2S,5R)-6,6-Dimethyl-3-oxobicyclo[3.1.1]heptan-2-yl)methanesulfonic acid ((2S)-**18**) (pyridine solvate).* An aqueous solution containing 0.135 g (2 mmol) of ClO_2_ was added to a solution of 0.184 g (1 mmol) of thiol (2*S*)-**8** in 20 mL of pyridine with stirring. After 1 h, the reaction mixture was evaporated and dissolved in Et_2_O. The precipitate containing C_5_H_5_N·HCl was removed. The solution containing the solvate of acid (2*S*)-**18** with pyridine was distilled off. Viscous liquid, 95% yield (0.60 g). ^1^H NMR spectrum (CDCl_3_, δ, ppm, *J*/Hz): 0.80 (s, 3H, H*^8^*), 1.23 (d, 1H, H*^7a^*, *J* = 10.6), 1.29 (s, 3H, H*^9^*), 2.05–2.13 (m, 1H, H*^5^*), 2.55–2.75 (m, 4H, H*^1^*, H*^4^*, H*^7b^*), 2.82 (dd, 1H, H*^10b^*, *J* = 14.1, 8.8), 3.05 (d, 1H, H*^2^*, *J* = 8.8), 3.62 (dd, 1H, H*^10b^*, *J* = 13.3, 3.6). ^13^C NMR spectrum (CDCl_3_, δ, ppm): 21.8 (C*^8^*), 26.5 (C*^9^*), 33.7 (C*^7^*), 38.5 (C*^5^*), 38.7 (C*^6^*), 42.5 (C*^1^*), 44.5 (C*^4^*), 52.9 (C*^10^*), 53.0 (C*^2^*), 212.1 (C*^3^*). IR spectrum (KBr, ν, cm^−1^): 478, 520, 603, 682, 754, 968, 997, 1035 st (SO_2_), 1163 st, 1999 st, 1226 st (SO_2_), 1386, 1485, 1714 st (C=O), 2883 st, 2924 st, 2972 st, 3439 st (OH); MS (ESI, 5 kV): *m/z* (%): 231.14 (100) [M–H]^−^; elemental analysis calcd (%) for C_10_H_16_O_4_S·5C_5_H_5_N: C 66.96, H 6.58, N 11.16, S 5.11; found: C 66.85, H 6.66, N 11.08, S 5.20.

*((1S,2R,5R)-6,6-Dimethyl-3-oxobicyclo[3.1.1]heptan-2-yl)methanesulfonic acid ((2R)-**18**).* An aqueous solution containing 0.135 g (2 mmol) of ClO_2_ was added to a solution of 0.184 g (1 mmol) of thiol (2*S*)-**8** in 20 mL of acetonitrile with stirring. After 1.5 h, it was extracted with chloroform (3 × 20 mL) and the aqueous fraction was evaporated. The dry residue of the aqueous fraction contained a mixture of acids (2*S*)-**18**: (2*R*)-**18** in the ratio of 1:2. The yield of a mixture of acids (2*S*)*-***18**, (2*R*)*-***18** was 0.223 g. NMR spectra were obtained by subtracting signals from the spectrum of a mixture of acids (2*S*)*-***18**, (2*R*)*-***18**. ^1^H NMR spectrum (CDCl_3_, δ, ppm, *J*/Hz): 0.90 (s, 3H, H*^8^*), 1.18 (d, 1H, H*^7a^*, *J* = 11.3), 1.34 (s, 3H, H*^9^*), 2.09–2.19 (m, 1H, H*^5^*), 2.39–2.47 (m, 1H, H*^1^*), 2.47–2.60 (m, 3H, H*^4^*, H*^7b^*), 2.87–3.01 (m, 1H, H*^10a^*), 3.01–3.10 (m, 1H, H*^2^*), 3.28 (d, 1H, H*^10b^*, *J* = 13.9). ^13^C NMR spectrum (D_2_O, δ, ppm): 19.1 (C*^8^*), 25.5 (C*^9^*), 28.4 (C*^7^*), 37.3 (C*^5^*), 38.4 (C*^6^*), 41.2 (C*^1^*), 44.2 (C*^4^*), 49.2 (C*^2^*), 50.7 (C*^10^*), 218.0 (C*^3^*). IR spectrum (KBr, ν, cm^−1^): 478, 590, 607, 756, 918, 1039 st (SO_2_), 1161 st, 1205 st, 1234 st (SO_2_), 1369, 1408, 1467, 1714 st (C=O), 2877 st, 2924 st, 2964 st, 3327 st (OH); MS (ESI, 5 kV): *m/z* (%): 231.17 (100) [M–H]^−^; elemental analysis calcd (%) for C_10_H_16_O_4_S: C 51.71, H 6.94, S 13.80; found: C 51.65, H 7.16, S 13.40.

*((1R,3R,5R)-6,6-Dimethyl-2-oxobicyclo[3.1.1]heptan-3-yl)methanesulfonic acid (**19**) (pyridine solvate)* was obtained similarly to acid (2*S*)-**18** from thiol **6**. Viscous liquid, 96% yield. ^1^H NMR spectrum (CDCl_3_, δ, ppm, *J*/Hz): 0.44 (s, 3H, H*^8^*), 1.06 (s, 3H, H*^9^*), 1.49–1.62 (m, 2H, H*^4a^*, H*^7a^*), 1.95–2.06 (m, 1H, H*^5^*), 2.15–2.27 (m, 1H, H*^7b^*), 2.33 (t, 1H, H*^1^*, *J* = 5.2), 2.43–2.51 (m, 1H, H*^4b^*), 2.55 (dd, 1H, H*^10a^*, *J* = 14.2, 9.6), 2.90–3.04 (m, 1H, H*^3^*), 3.50 (d, 1H, H*^10b^*, *J* = 13.9). ^13^C NMR spectrum (CDCl_3_, δ, ppm): 21.4 (C*^8^*), 24.6 (C*^7^*), 25.6 (C*^9^*), 29.4 (C*^4^*), 39.9 (C*^3^*), 40.2 (C*^5^*), 42.3 (C*^6^*), 51.4 (C*^10^*), 56.8 (C*^1^*), 127.1, 141.0, 146.1 (C*^Py^*), 213.2 (C*^2^*). IR spectrum (KBr, ν, cm^−1^): 528, 609, 682, 756, 999, 1039 (SO_2_), 1178 (SO_2_), 1195 (SO_2_), 1242 (SO_2_), 1388, 1462, 1705 (C=O), 2873, 2949, 3435 (OH); MS (ESI, 5 kV): *m/z* (%): 231.14 (100) [M–1]^−^; elemental analysis calcd (%) for C_10_H_16_O_4_S·4C_5_H_5_N: C 65.66, H 6.61, N 10.21, S 5.84; found: C 65.85, H 6.70, N 10.11, S 5.64.

## Figures and Tables

**Table 1:** Effect of reaction conditions on the stereoselectivity of the formation of (*S*)-acyl derivatives (2*S*)-**11** and (2*S*)-**12** (*de*) (synthesis time 5 h).

Product	Solvent	Temperature, °C	Yield of Diastereomers, %	*de*, %
(2*S*)-**11** [8]	−	−5	95	33
(2*S*)-**11**	−	−50	83	69
(2*S*)-**11**	CH_2_Cl_2_	−65	78	85
(2*S*)-**11**	THF	−65	84	92
(2*S*)-**12**	CH_2_Cl_2_	−65	70	88
(2*S*)-**12**	THF	−65	68	93

## Data Availability

Not applicable.

## Supplementary Materials

The ^1^H NMR, ^13^C NMR, NOESY, IR, and mass spectra of novel compounds.

## Sample Availability

Samples of the compounds are not available from the authors but can be specially prepared upon prior request.
