# Supplementary material for: Synthesis and Oxidative Transformations of New Chiral Pinane-Type γ-Ketothiols: Stereochemical Features of Reactions
Source: Molecules. 2021 Aug 29;26(17):5245. doi: 10.3390/molecules26175245 (PMC8433878; doi:10.3390/molecules26175245)
Supplement: Supplementary file 1 [file molecules-26-05245-s001.zip › molecules-1338561-supplementary.pdf]

Molecules

Supplementary Materials

Synthesis and Oxidative Transformations of New Chiral Pinane-Type γ-Ketothiols: Stereochemical Features of Reactions

Olga M. Lezina, Denis V. Sudarikov*, Svetlana N. Subbotina, Larisa L. Frolova, and Svetlana A. Rubtsova

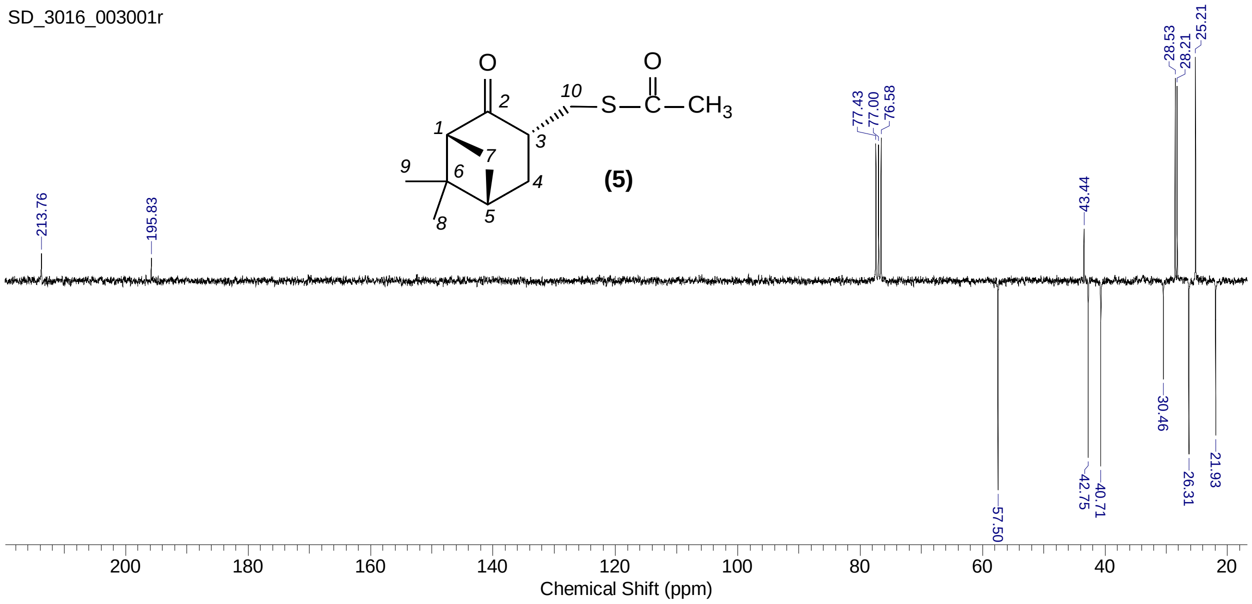
SD_3016_003001r
(5)
213.76
195.83
77.43
77.00
76.58
43.44
28.53
28.21
25.21
57.50
42.75
40.71
30.46
26.31
21.93
200
180
160
140
120
100
80
60
40
20
Chemical Shift (ppm)

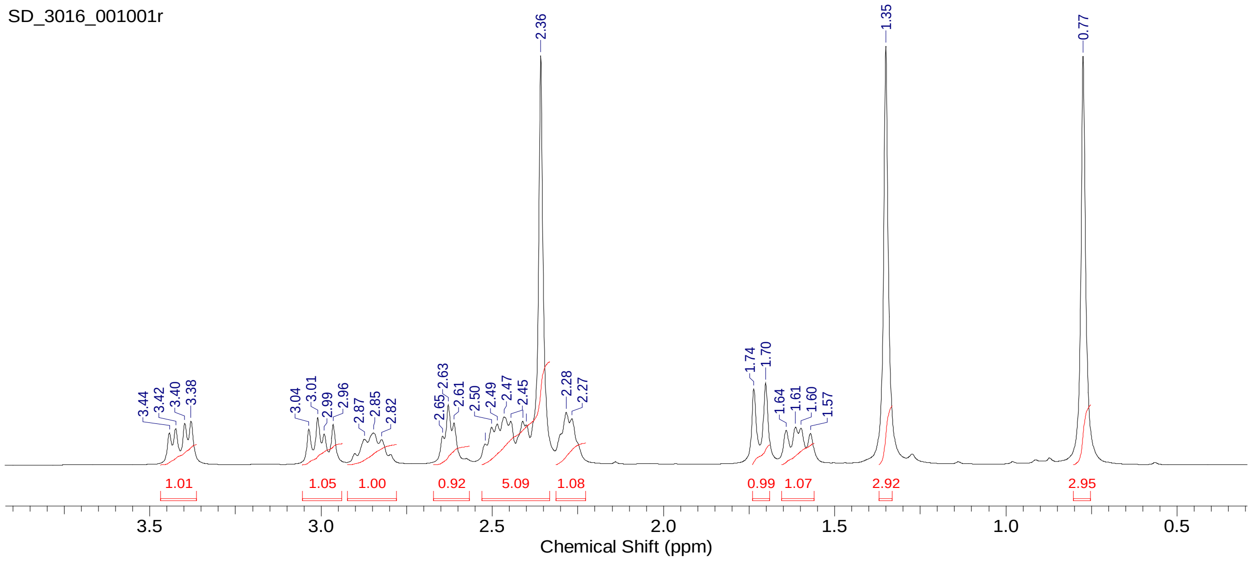
SD_3016_001001r
3.44
3.42
3.40
3.38
3.04
3.01
2.99
2.96
2.87
2.85
2.82
2.65
2.63
2.61
2.50
2.49
2.47
2.45
2.36
2.28
2.27
1.74
1.70
1.64
1.61
1.60
1.57
1.35
0.77
1.01
1.05
1.00
0.92
5.09
1.08
0.99
1.07
2.92
2.95
3.5
3.0
2.5
2.0
1.5
1.0
0.5
Chemical Shift (ppm)

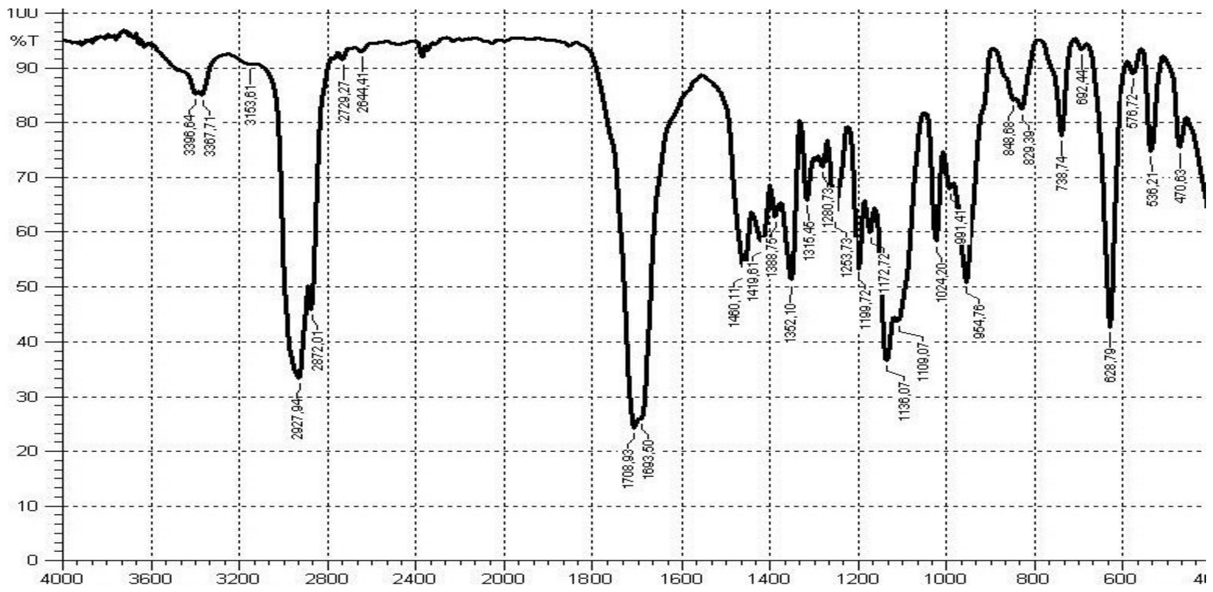
%T
3396.64
3387.71
3153.61
2927.94
2872.01
2729.27
2644.41
1708.93
1693.50
1460.11
1419.61
1388.75
1352.10
1315.45
1280.73
1253.73
1199.72
1172.72
1136.07
1109.07
1024.20
991.41
954.76
848.68
829.39
738.74
692.44
628.79
576.72
538.21
470.63

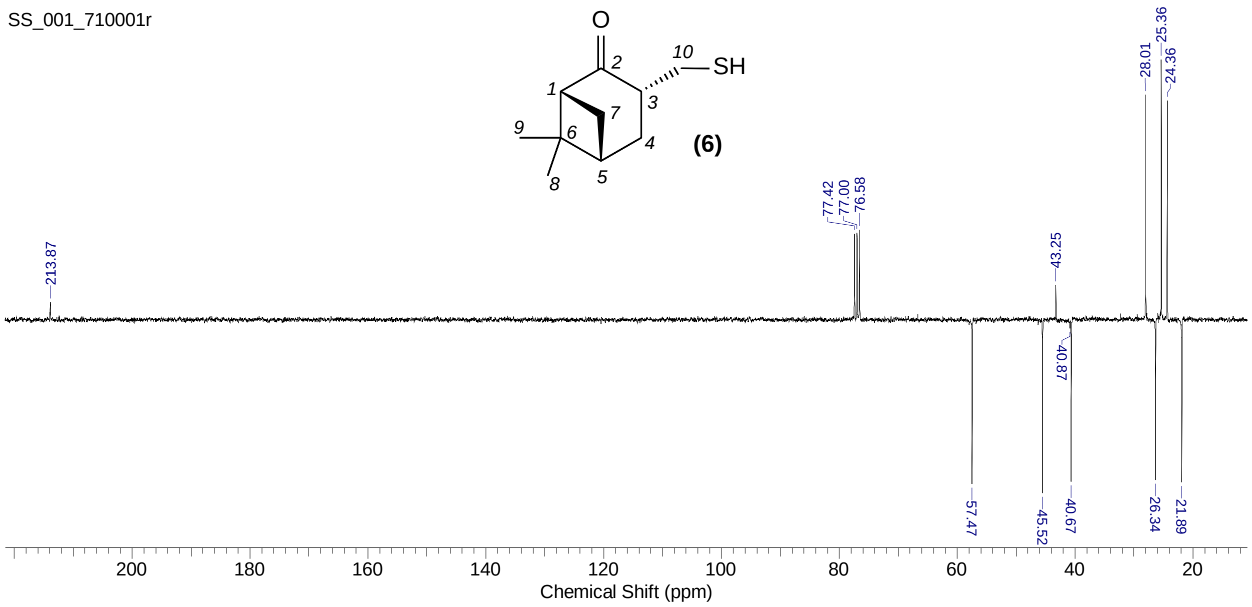
SS_001_710001r
(6)
Chemical Shift (ppm)

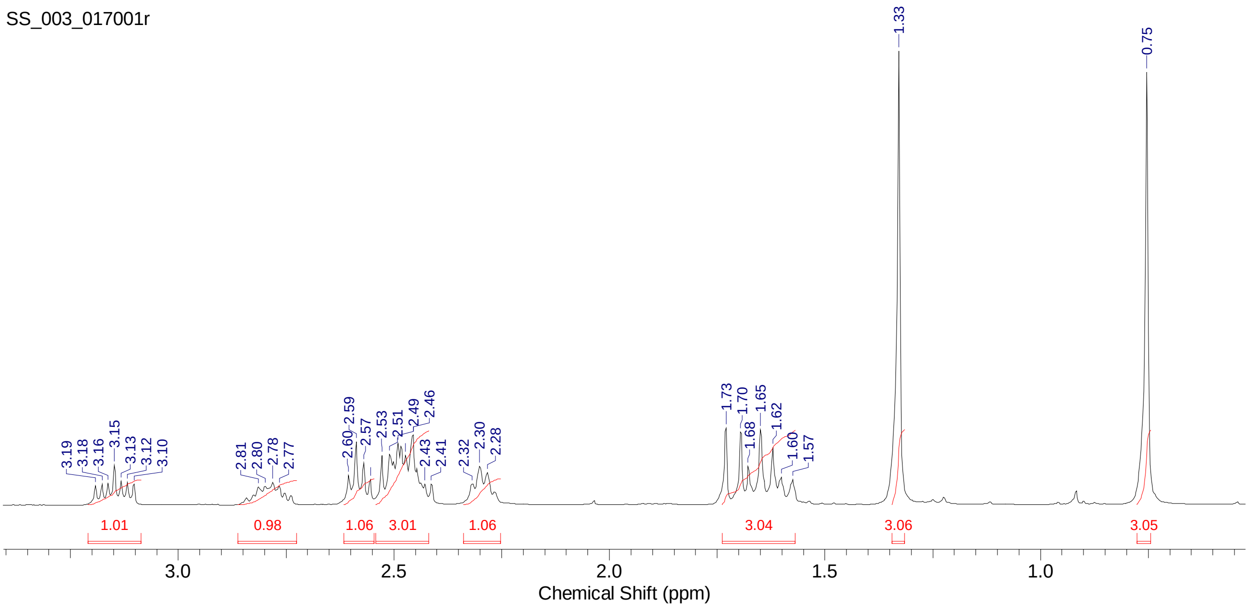
SS_003_017001r
Chemical Shift (ppm)

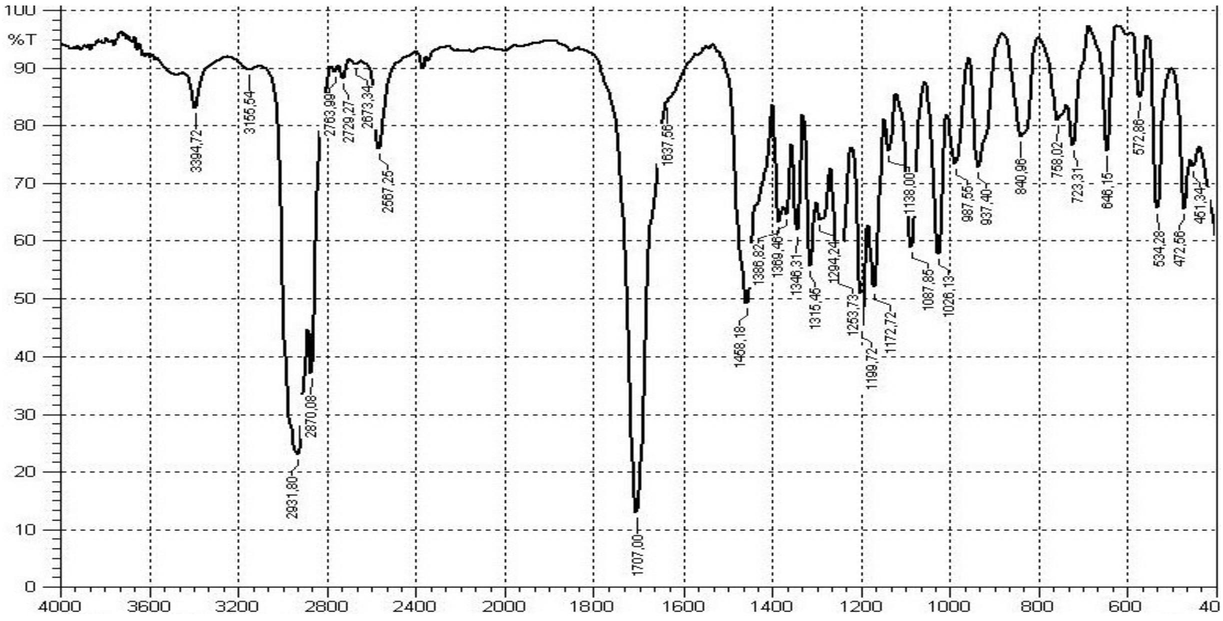
%T

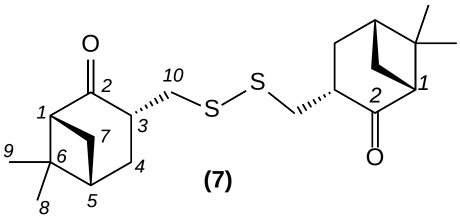
O
2
10
S
S
1
3
7
9
6
4
8
5
2
1
O

(7)

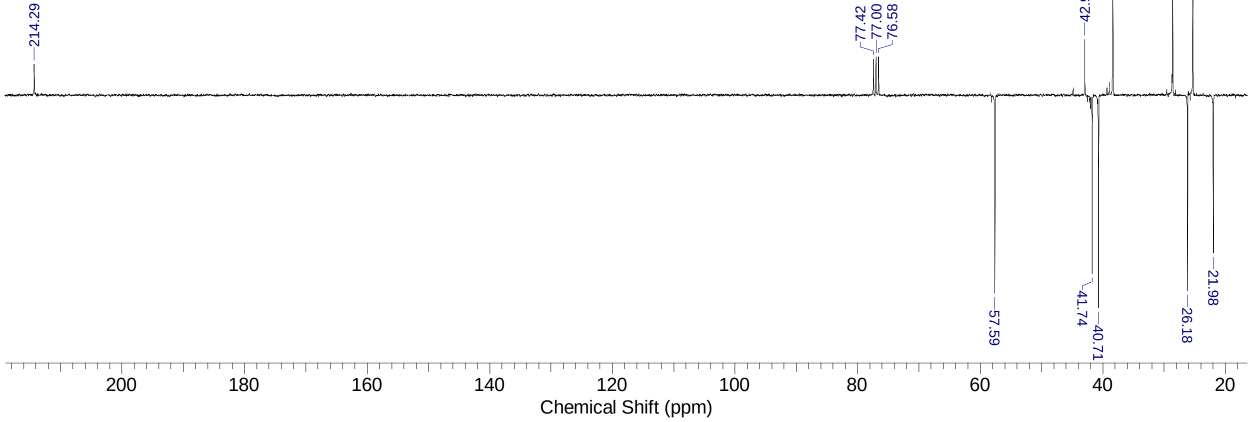
SS_003_140001r
214.29
77.42
77.00
76.58
42.94
38.37
28.61
25.32
57.59
41.74
40.71
26.18
21.98
200
180
160
140
120
100
80
60
40
20
Chemical Shift (ppm)

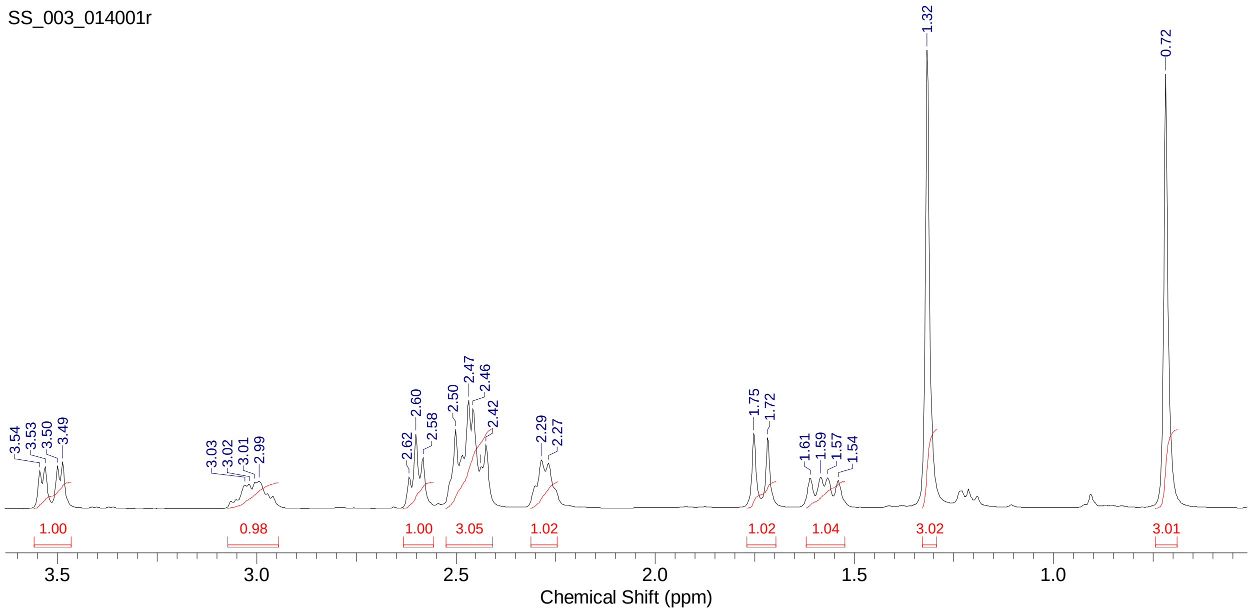
SS_003_014001r
3.54
3.53
3.50
3.49
3.03
3.02
3.01
2.99
2.62
2.60
2.58
2.50
2.47
2.46
2.42
2.29
2.27
1.75
1.72
1.61
1.59
1.57
1.54
1.32
0.72
1.00
0.98
1.00
3.05
1.02
1.02
1.04
3.02
3.01
3.5
3.0
2.5
2.0
1.5
1.0
Chemical Shift (ppm)

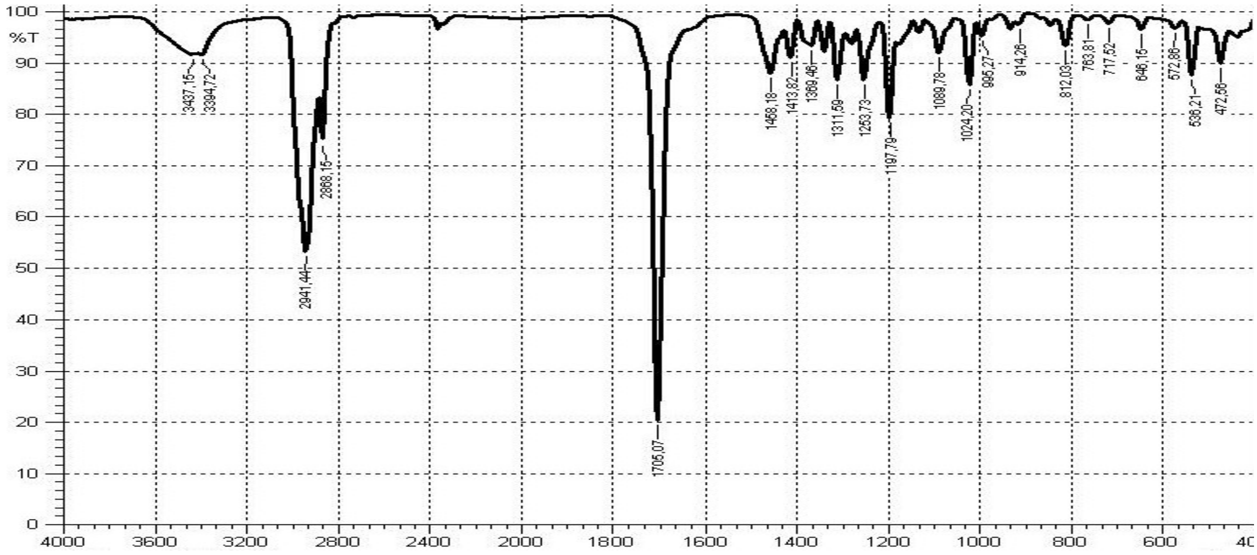
%T
3437.15
3394.72
2941.44
2868.15
1705.07
1468.18
1413.82
1369.46
1311.59
1253.73
1197.79
1089.78
1024.20
995.27
914.26
812.03
763.81
717.52
646.15
572.88
538.21
472.56

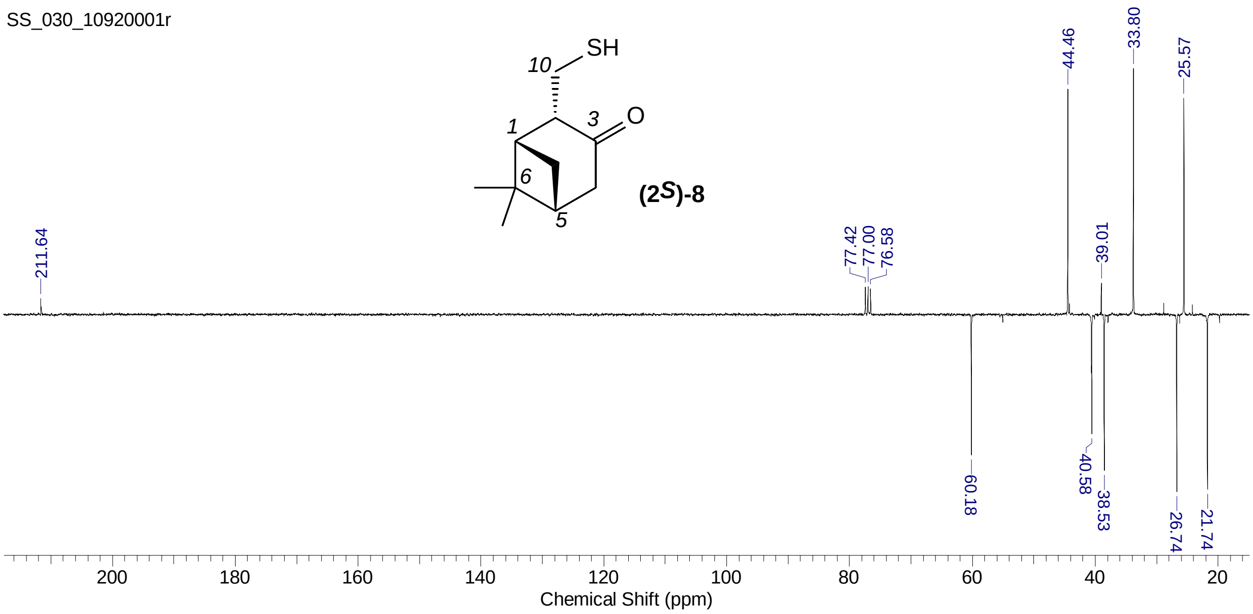
SS_030_10920001r
SH
10
1
3
O
6
5
(2S)-8
211.64
77.42
77.00
76.58
44.46
39.01
33.80
25.57
60.18
40.58
38.53
26.74
21.74
Chemical Shift (ppm)

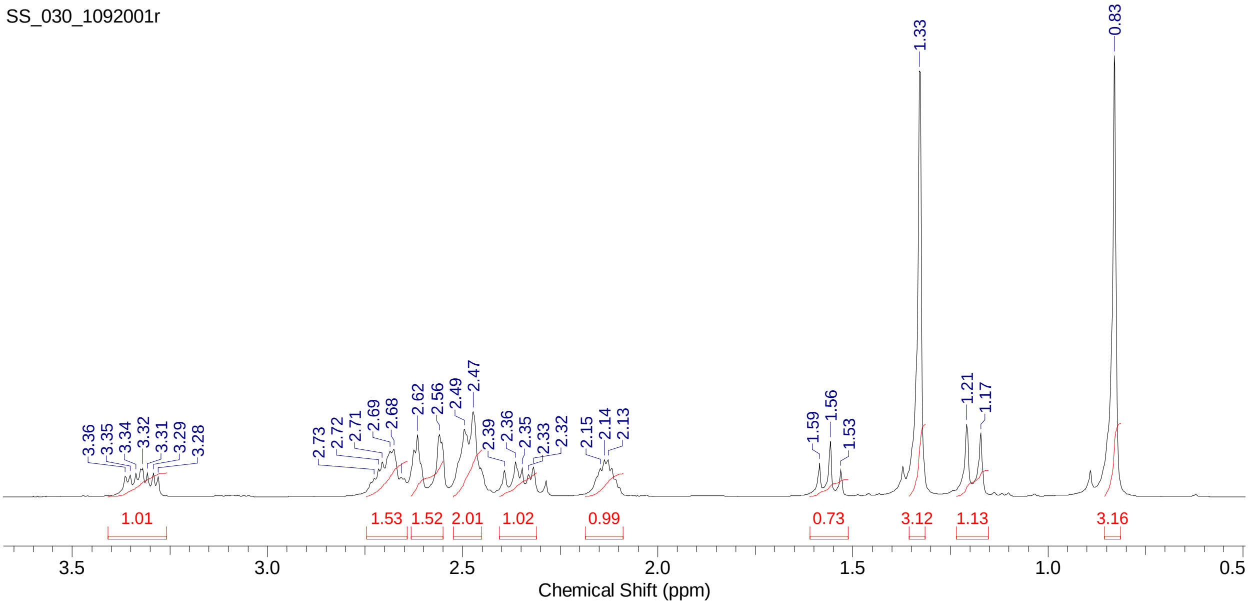
SS_030_1092001r
3.36
3.35
3.34
3.32
3.31
3.29
3.28
2.73
2.72
2.71
2.69
2.68
2.62
2.56
2.49
2.47
2.39
2.36
2.35
2.33
2.32
2.15
2.14
2.13
1.59
1.56
1.53
1.33
1.21
1.17
0.83
1.01
1.53
1.52
2.01
1.02
0.99
0.73
3.12
1.13
3.16
Chemical Shift (ppm)

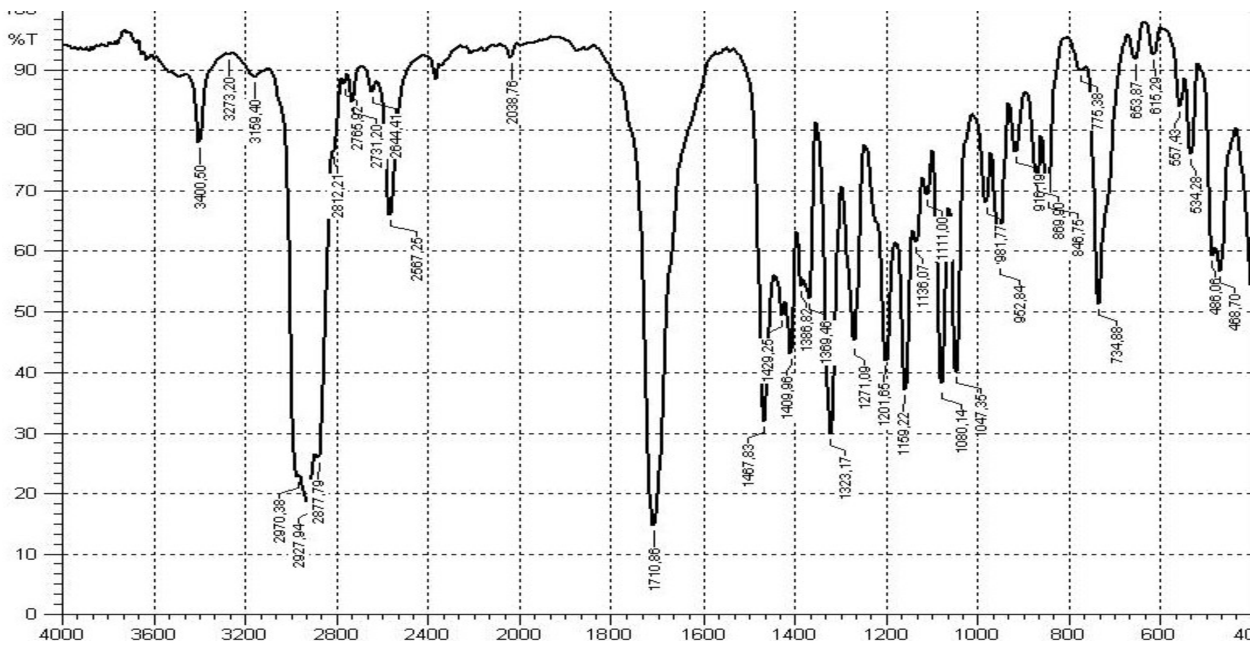
%T
3400,50
3273,20
3159,40
2970,38
2927,94
2877,79
2812,21
2765,92
2731,20
2644,41
2587,25
2038,76
1710,86
1467,83
1429,25
1409,96
1386,82
1369,46
1323,17
1271,09
1201,65
1159,22
1136,07
1111,00
1080,14
1047,35
981,77
952,84
916,19
869,90
846,75
775,38
734,88
653,87
615,29
557,43
534,28
486,06
468,70

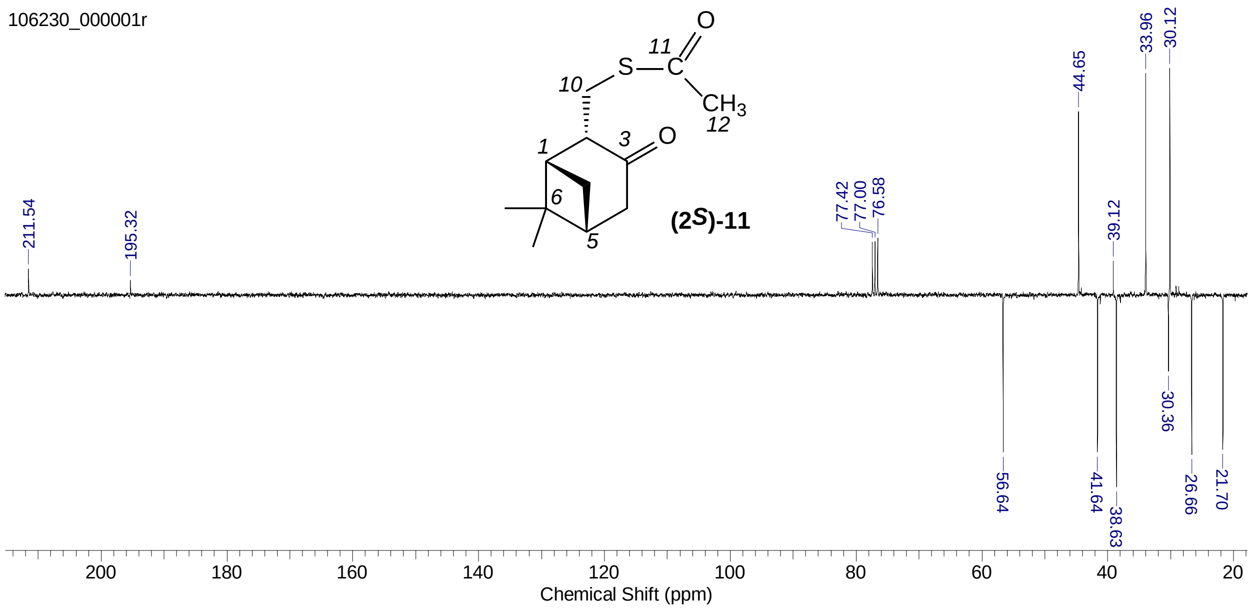
106230_000001r
(2S)-11
211.54
195.32
77.42
77.00
76.58
56.64
44.65
41.64
39.12
38.63
33.96
30.36
30.12
26.66
21.70
Chemical Shift (ppm)

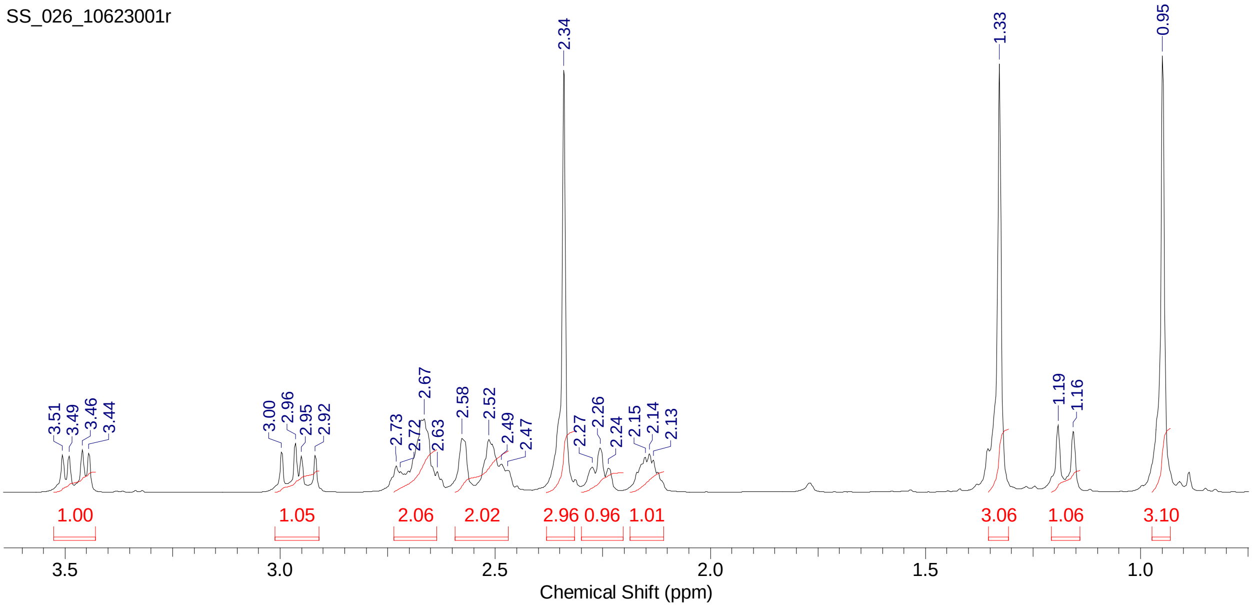
SS_026_10623001r
3.51
3.49
3.46
3.44
3.00
2.96
2.95
2.92
2.73
2.72
2.67
2.63
2.58
2.52
2.49
2.47
2.34
2.27
2.26
2.24
2.15
2.14
2.13
1.33
1.19
1.16
0.95
1.00
1.05
2.06
2.02
2.96
0.96
1.01
3.06
1.06
3.10
Chemical Shift (ppm)

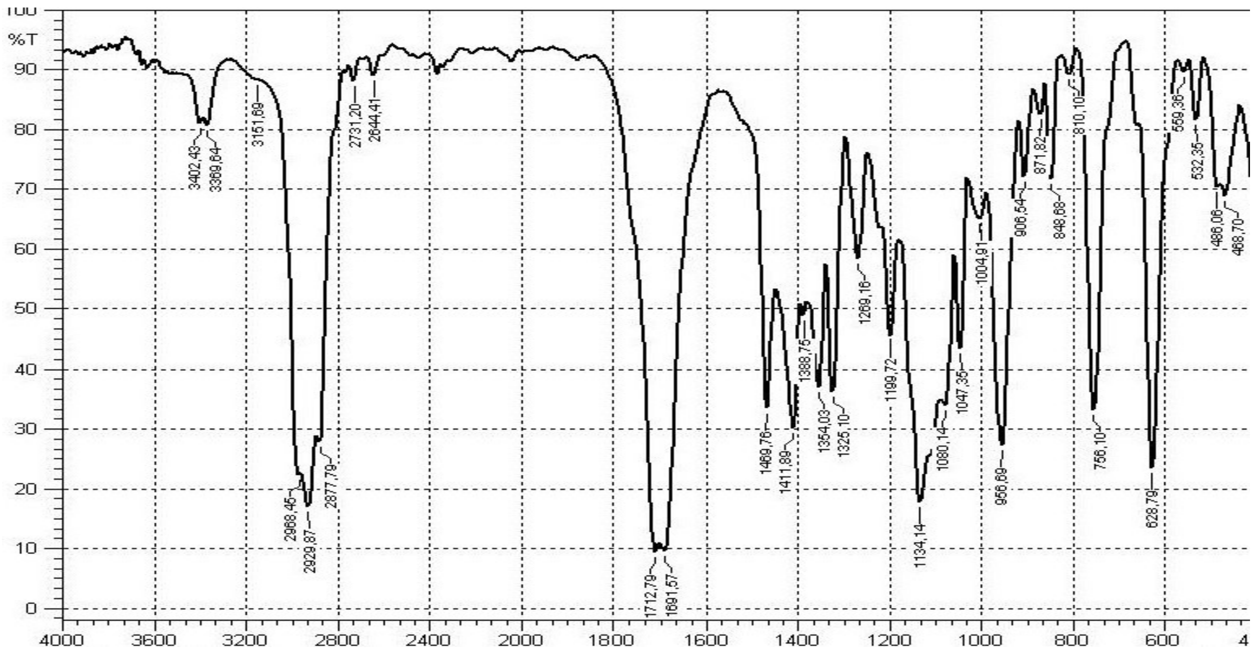
%T
3402,43
3369,64
3151,69
2968,45
2929,87
2877,79
2731,20
2644,41
1712,79
1691,57
1469,76
1411,89
1388,75
1354,03
1325,10
1269,16
1199,72
1134,14
1080,14
1047,35
1004,91
958,69
906,54
871,82
848,68
810,10
756,10
628,78
569,36
532,35
486,06
468,70

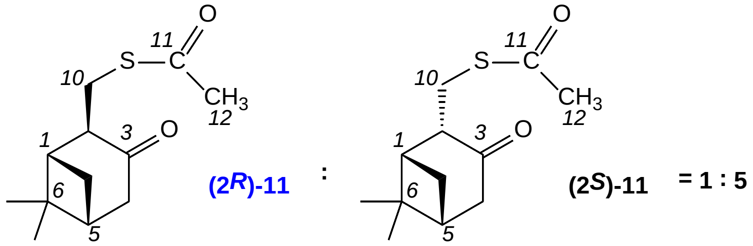
O
11
S—C
10
CH3
12
1
3
O
6
5
(2R)-11
:
(2S)-11
= 1 : 5

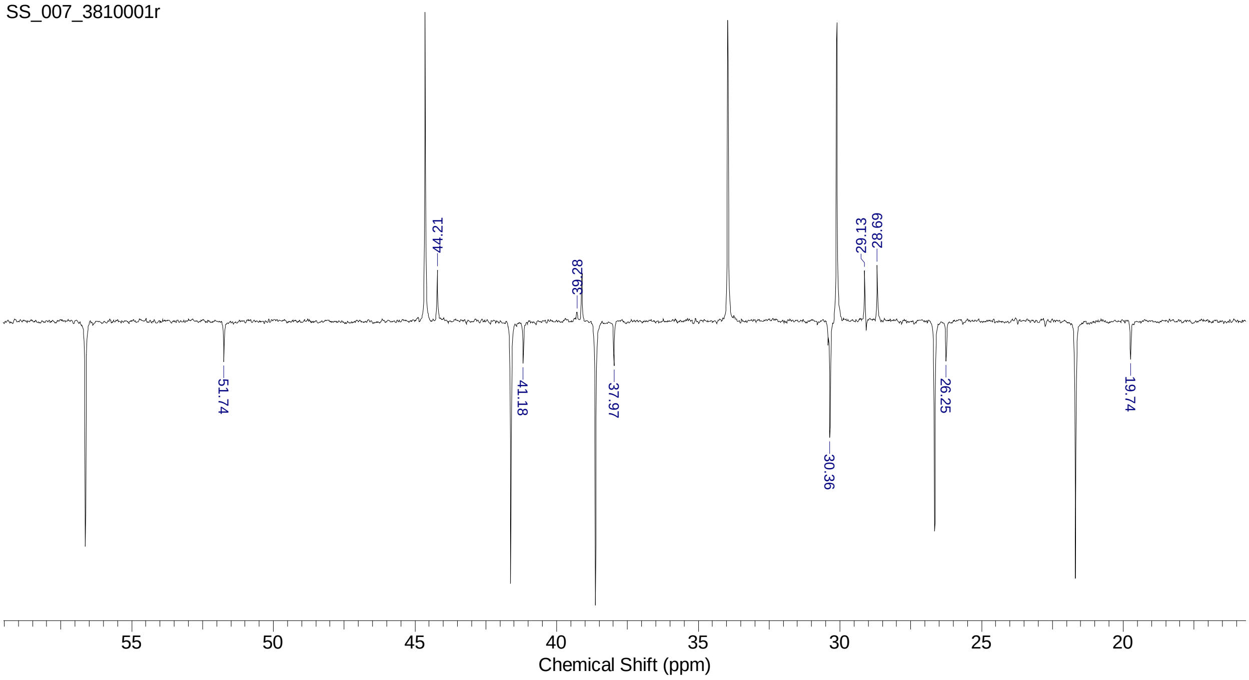
SS_007_3810001r
44.21
39.28
29.13
28.69
51.74
41.18
37.97
30.36
26.25
19.74
55
50
45
40
35
30
25
20
Chemical Shift (ppm)

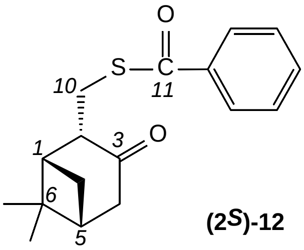
O
S
C
10
11
1
3
O
6
5

(2*S*)-12

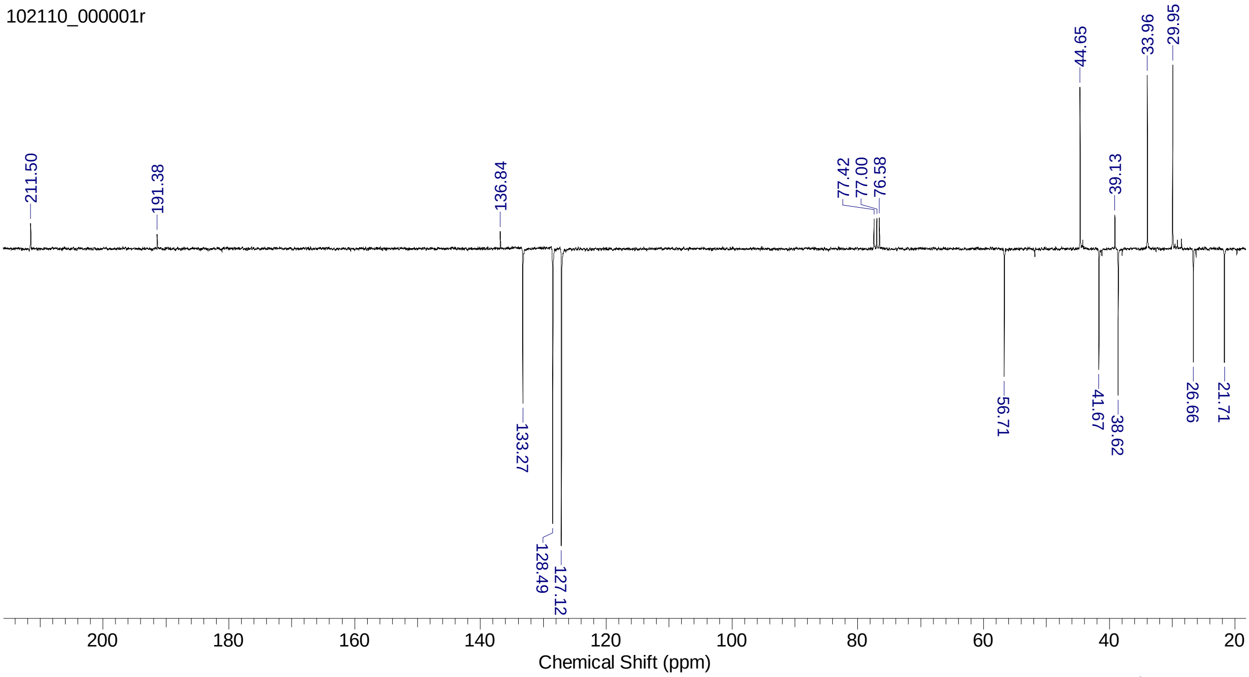
102110_000001r
211.50
191.38
136.84
133.27
128.49
127.12
77.42
77.00
76.58
56.71
44.65
41.67
39.13
38.62
33.96
29.95
26.66
21.71
200
180
160
140
120
100
80
60
40
20
Chemical Shift (ppm)

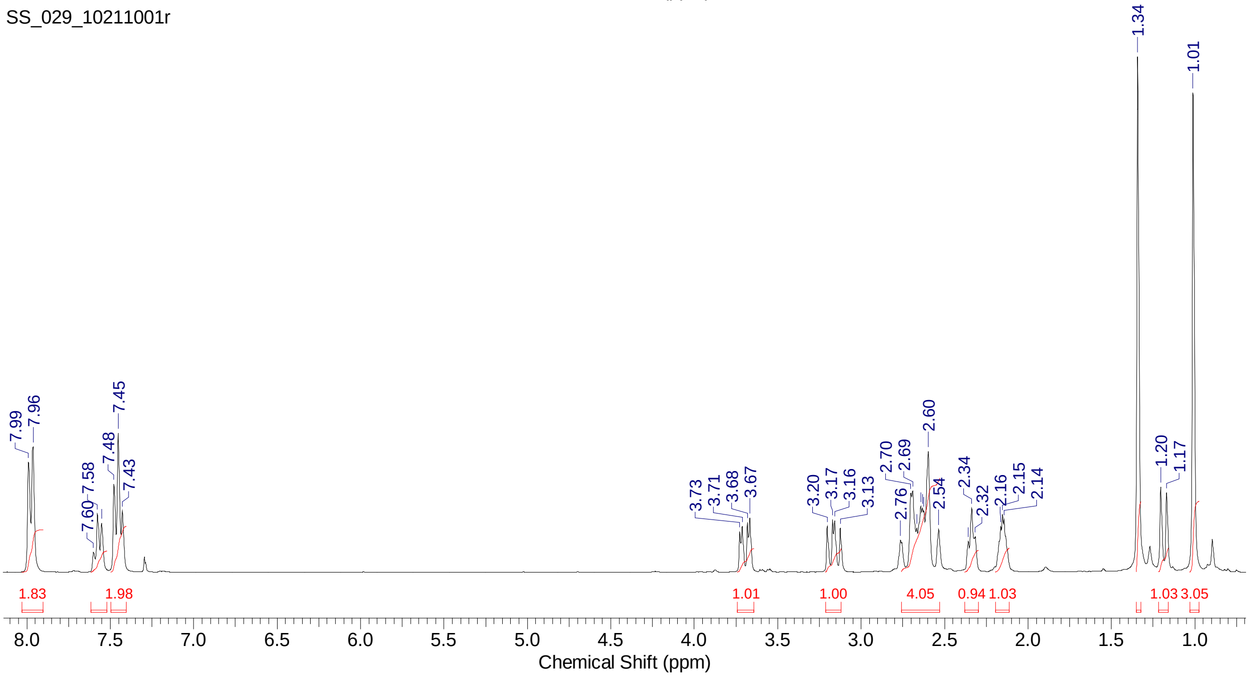
SS_029_10211001r
7.99
7.96
7.60
7.58
7.48
7.45
7.43
3.73
3.71
3.68
3.67
3.20
3.17
3.16
3.13
2.76
2.70
2.69
2.60
2.54
2.34
2.32
2.16
2.15
2.14
1.34
1.20
1.17
1.01
1.83
1.98
1.01
1.00
4.05
0.94
1.03
1.03
3.05
8.0
7.5
7.0
6.5
6.0
5.5
5.0
4.5
4.0
3.5
3.0
2.5
2.0
1.5
1.0
Chemical Shift (ppm)

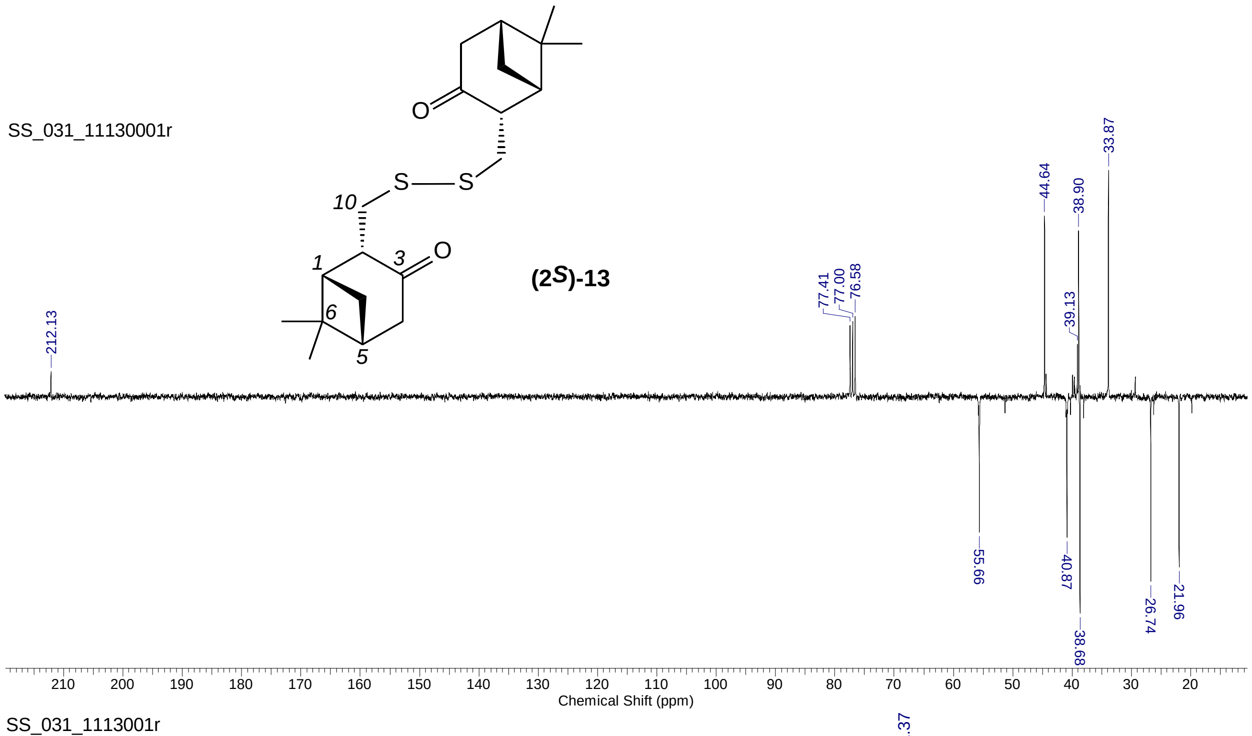
SS_031_11130001r
10
S
S
O
1
3
O
6
5
(2S)-13
212.13
77.41
77.00
76.58
44.64
38.90
33.87
39.13
55.66
40.87
38.68
26.74
21.96
210 200 190 180 170 160 150 140 130 120 110 100 90 80 70 60 50 40 30 20
Chemical Shift (ppm)

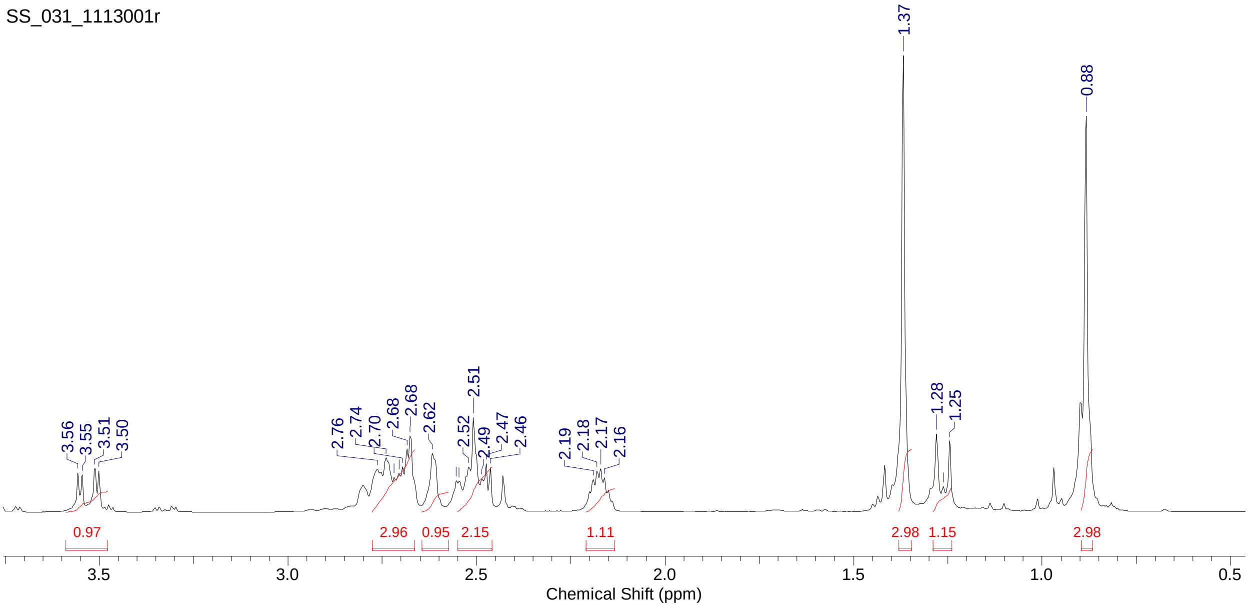
SS_031_1113001r
1.37
0.88
3.56
3.55
3.51
3.50
2.76
2.74
2.70
2.68
2.68
2.62
2.52
2.51
2.49
2.47
2.46
2.19
2.18
2.17
2.16
1.28
1.25
0.97
2.96
0.95
2.15
1.11
2.98
1.15
2.98
3.5 3.0 2.5 2.0 1.5 1.0 0.5
Chemical Shift (ppm)

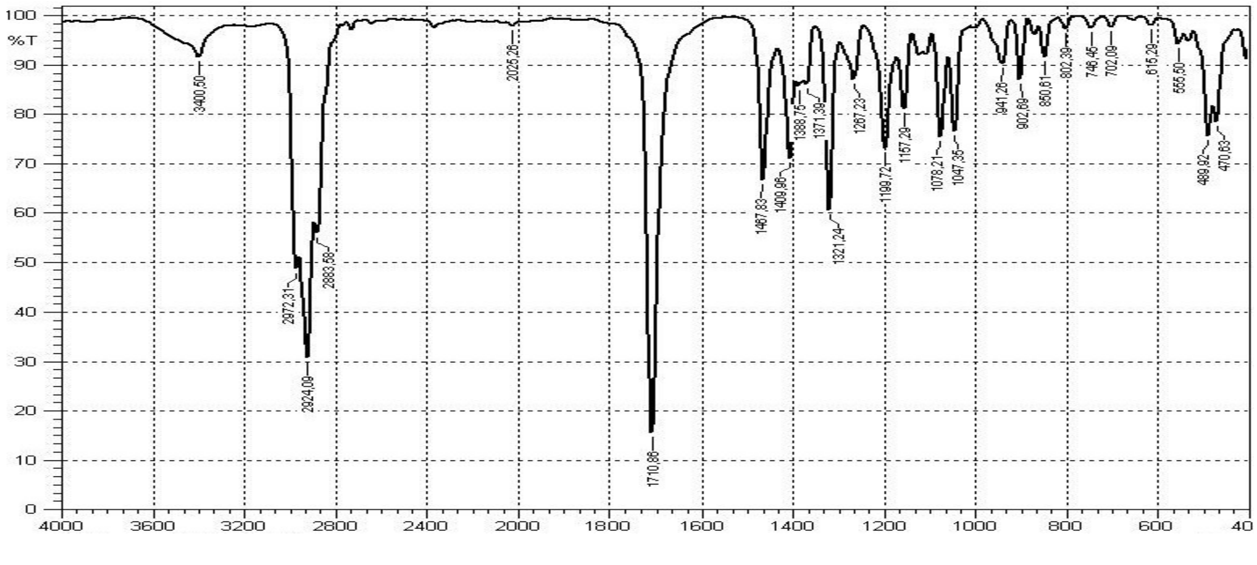
100
%T
90
80
70
60
50
40
30
20
10
0
3400,50
2972,31
2924,09
2883,58
2025,26
1710,88
1467,83
1409,96
1388,75
1371,39
1321,24
1267,23
1199,72
1157,29
1078,21
1047,35
941,26
902,69
850,61
802,39
746,45
702,09
615,29
555,50
489,92
470,63
4000 3600 3200 2800 2400 2000 1800 1600 1400 1200 1000 800 600 40

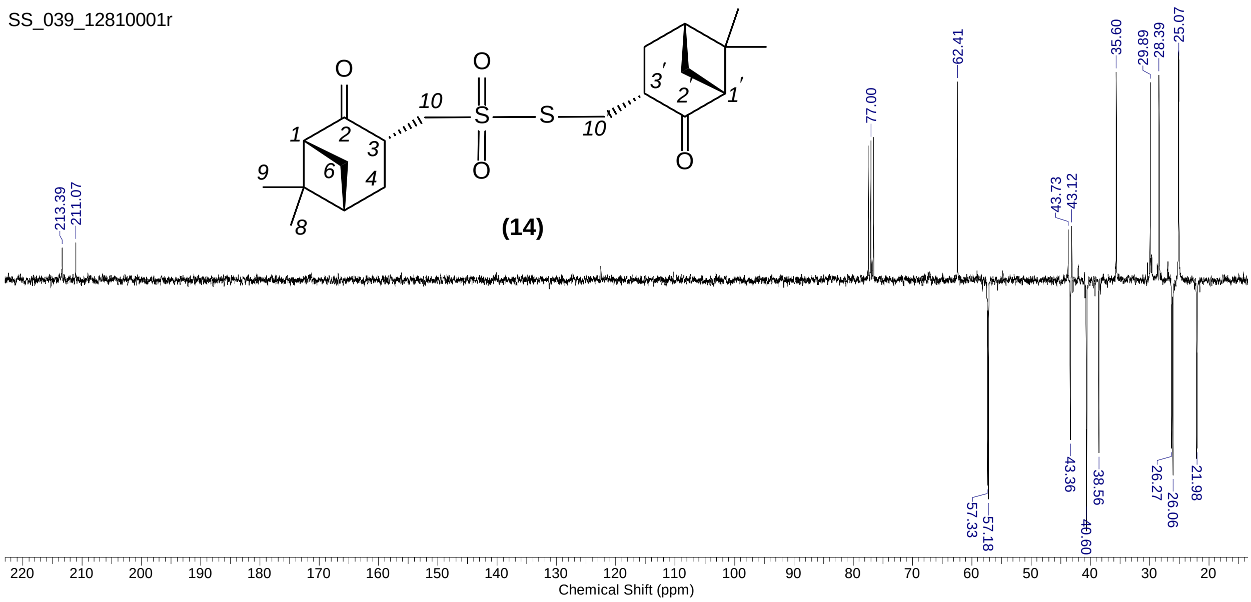
SS_039_12810001r
(14)
213.39
211.07
77.00
62.41
43.73
43.12
35.60
29.89
28.39
25.07
57.33
57.18
43.36
40.60
38.56
26.27
26.06
21.98
Chemical Shift (ppm)

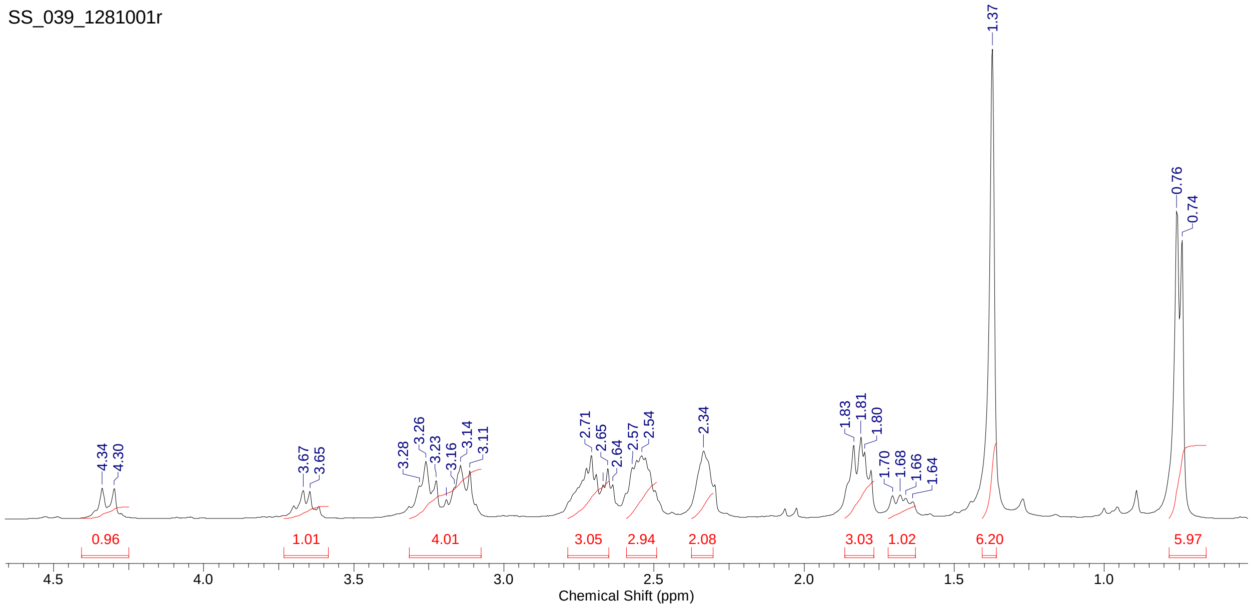
SS_039_1281001r
1.37
0.76
0.74
4.34
4.30
3.67
3.65
3.28
3.26
3.23
3.16
3.14
3.11
2.71
2.65
2.64
2.57
2.54
2.34
1.83
1.81
1.80
1.70
1.68
1.66
1.64
0.96
1.01
4.01
3.05
2.94
2.08
3.03
1.02
6.20
5.97
Chemical Shift (ppm)

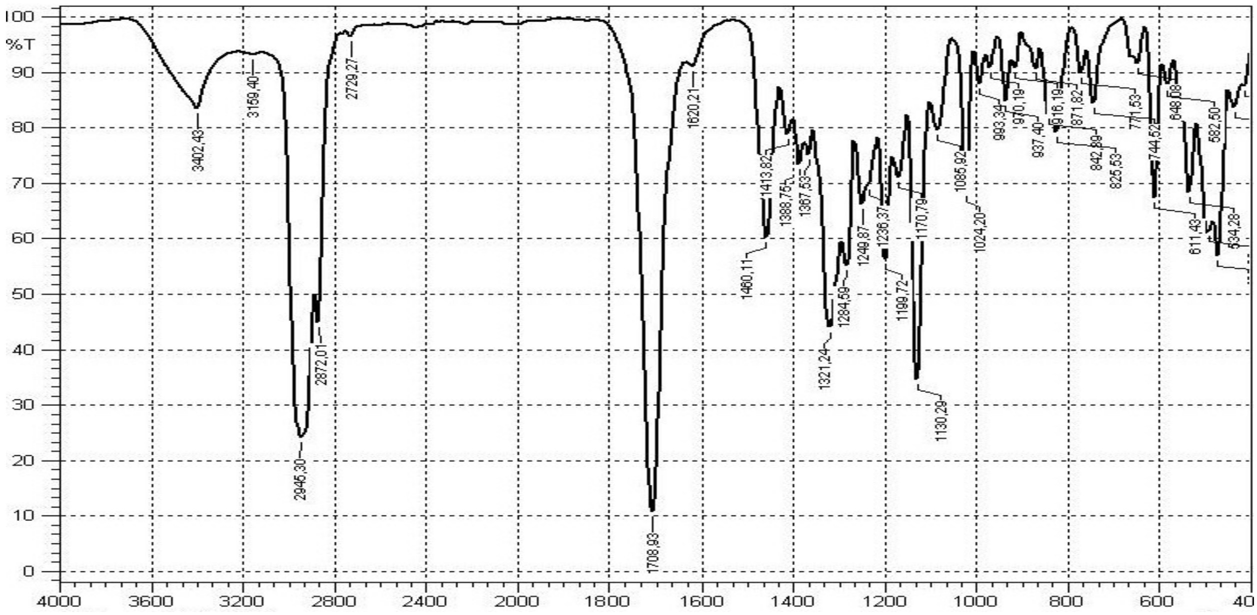
%T
3402,43
3159,40
2945,30
2872,01
2729,27
1708,93
1620,21
1460,11
1413,82
1388,75
1367,53
1321,24
1284,59
1249,87
1236,37
1199,72
1170,79
1130,29
1085,92
1024,20
983,34
970,19
937,40
916,19
871,82
842,89
825,53
771,53
744,52
648,08
611,43
582,50
534,28

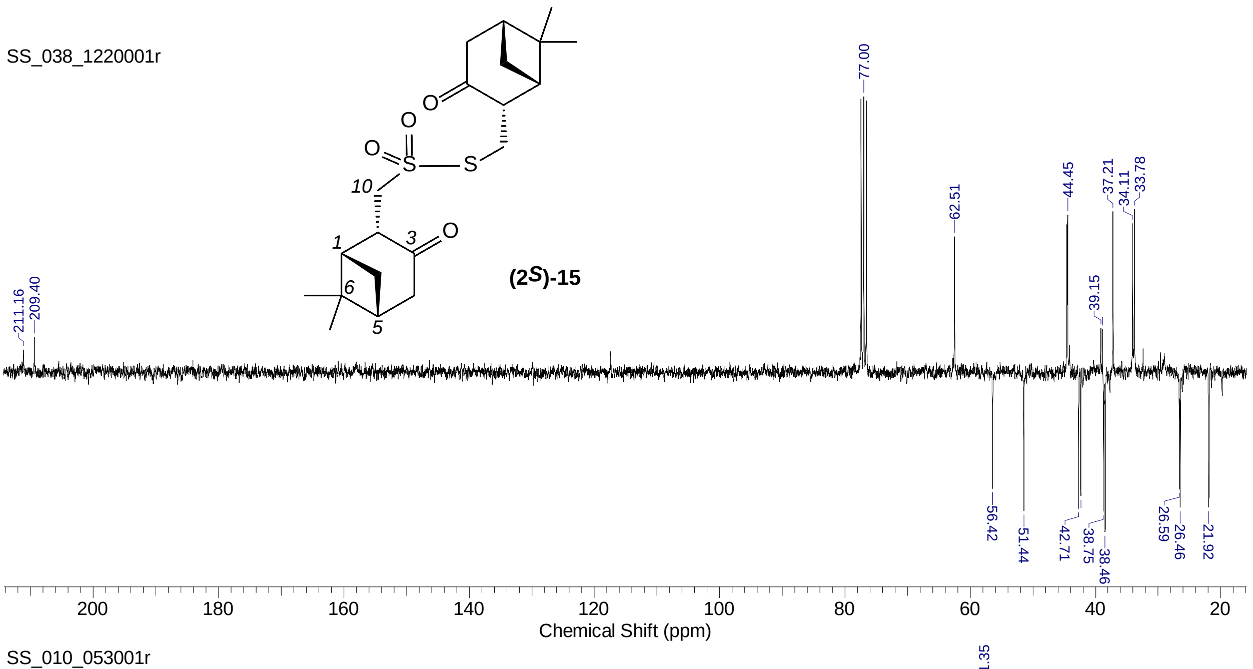
SS_038_1220001r
(2S)-15
211.16
209.40
77.00
62.51
44.45
39.15
37.21
34.11
33.78
56.42
51.44
42.71
38.75
38.46
26.59
26.46
21.92
Chemical Shift (ppm)

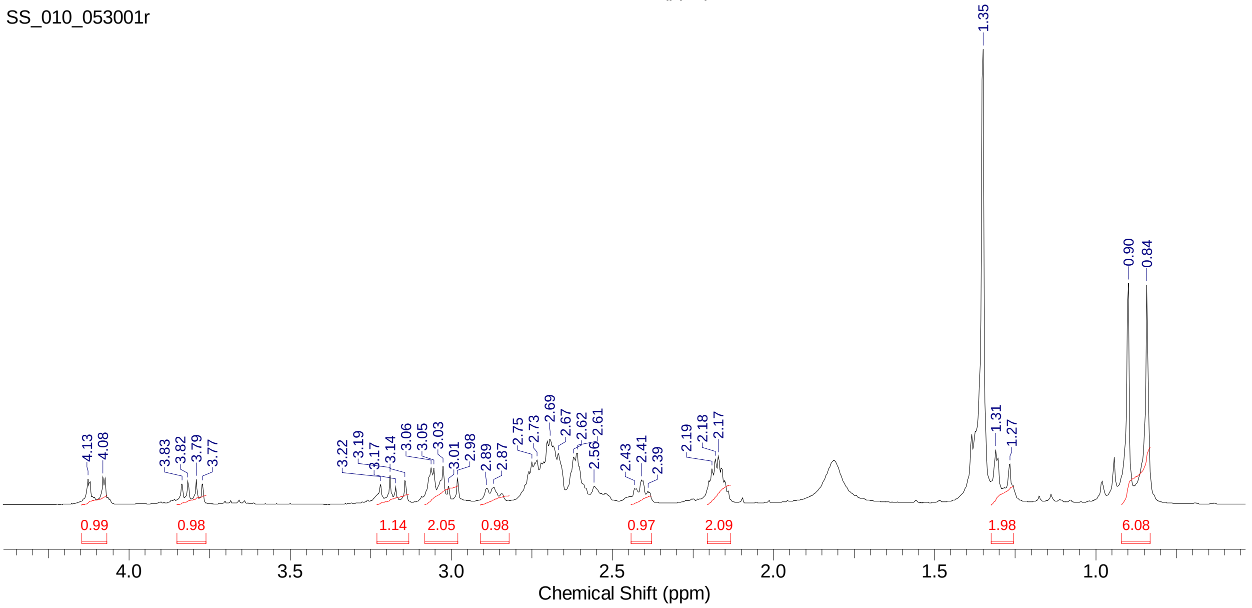
SS_010_053001r
1.35
0.90
0.84
4.13
4.08
3.83
3.82
3.79
3.77
3.22
3.19
3.17
3.14
3.06
3.05
3.03
3.01
2.98
2.89
2.87
2.75
2.73
2.69
2.67
2.62
2.61
2.56
2.43
2.41
2.39
2.19
2.18
2.17
1.31
1.27
0.99
0.98
1.14
2.05
0.98
0.97
2.09
1.98
6.08
Chemical Shift (ppm)

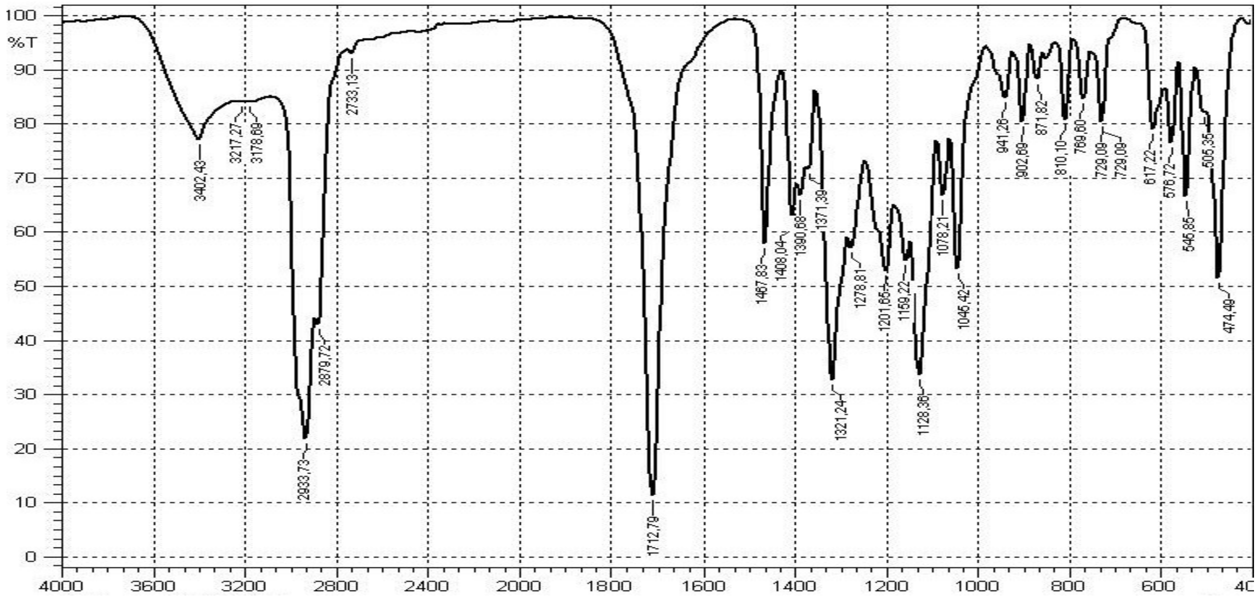
%T
3402.43
3217.27
3178.69
2933.73
2879.72
2733.13
1712.79
1467.83
1408.04
1390.68
1371.39
1321.24
1278.81
1201.65
1159.22
1128.36
1078.21
1045.42
941.26
902.69
871.82
810.10
769.60
729.09
617.22
576.72
545.85
505.35
474.49

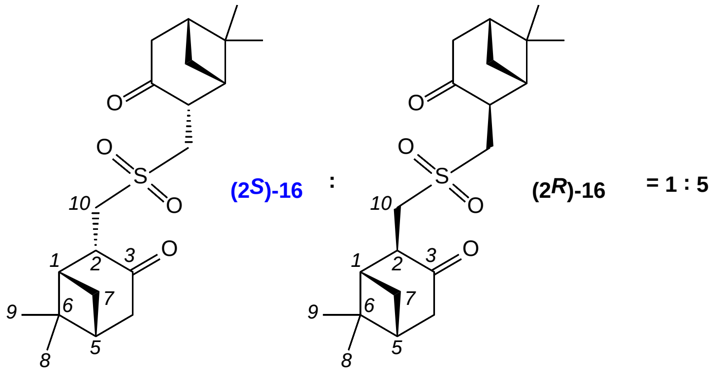
(2S)-16
:
(2R)-16
= 1 : 5
10
1
2
3
6
7
9
8
5

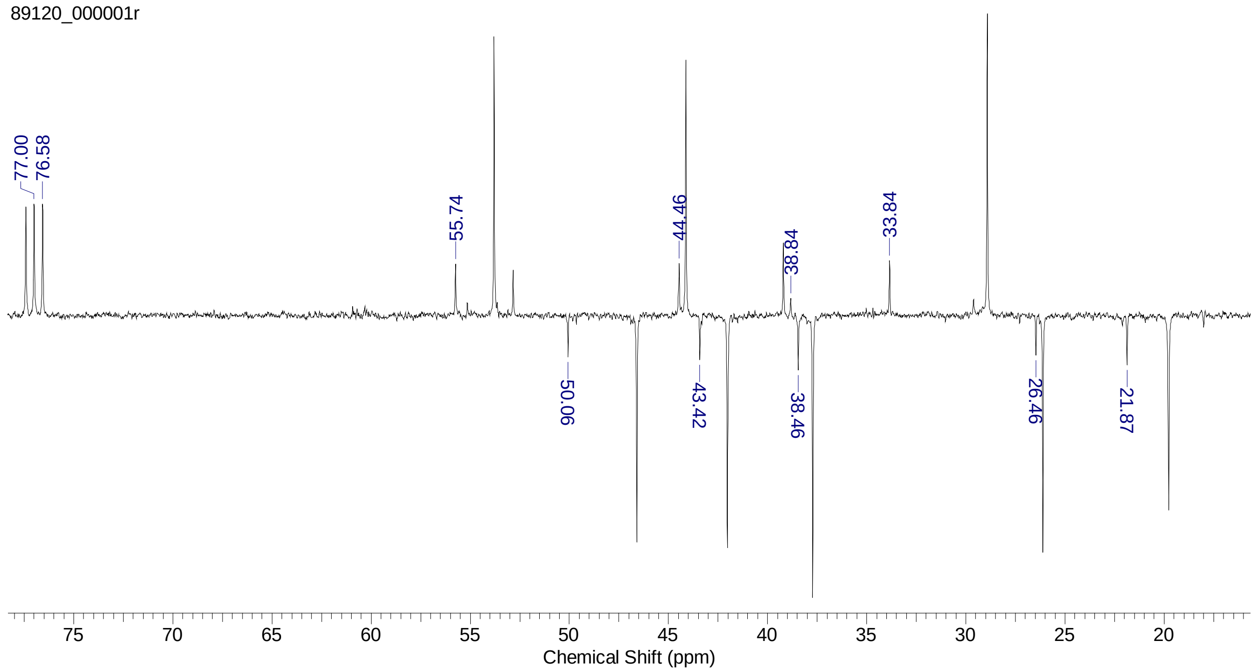
89120_000001r
77.00
76.58
55.74
44.46
38.84
33.84
50.06
43.42
38.46
26.46
21.87
75
70
65
60
55
50
45
40
35
30
25
20
Chemical Shift (ppm)

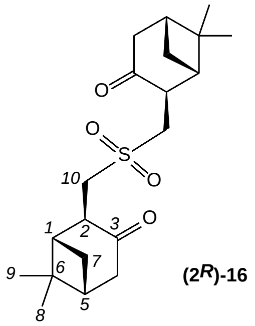

(2*R*)-16

89140_000001r

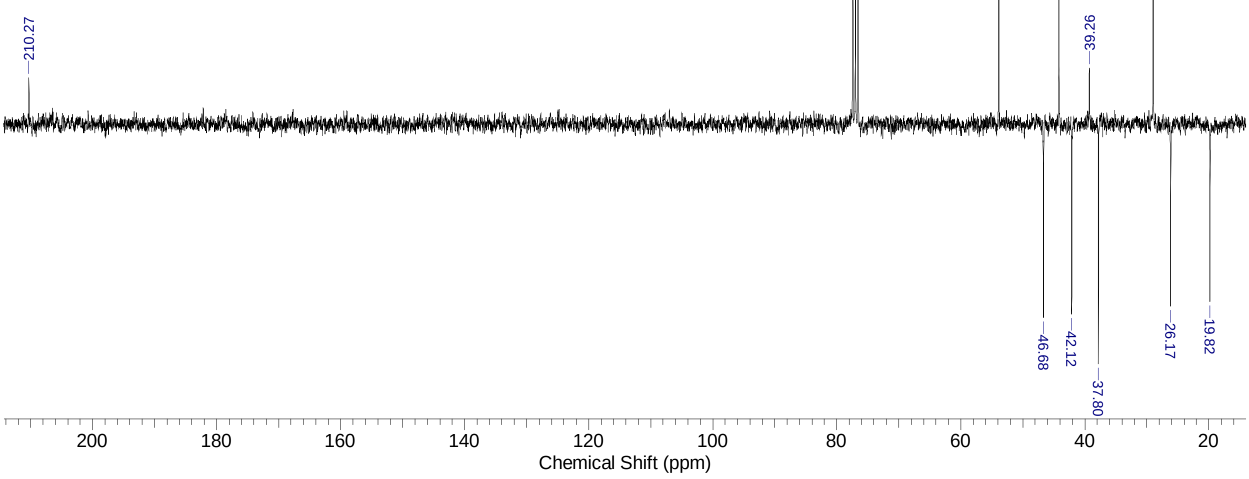

SS_043_8914001r

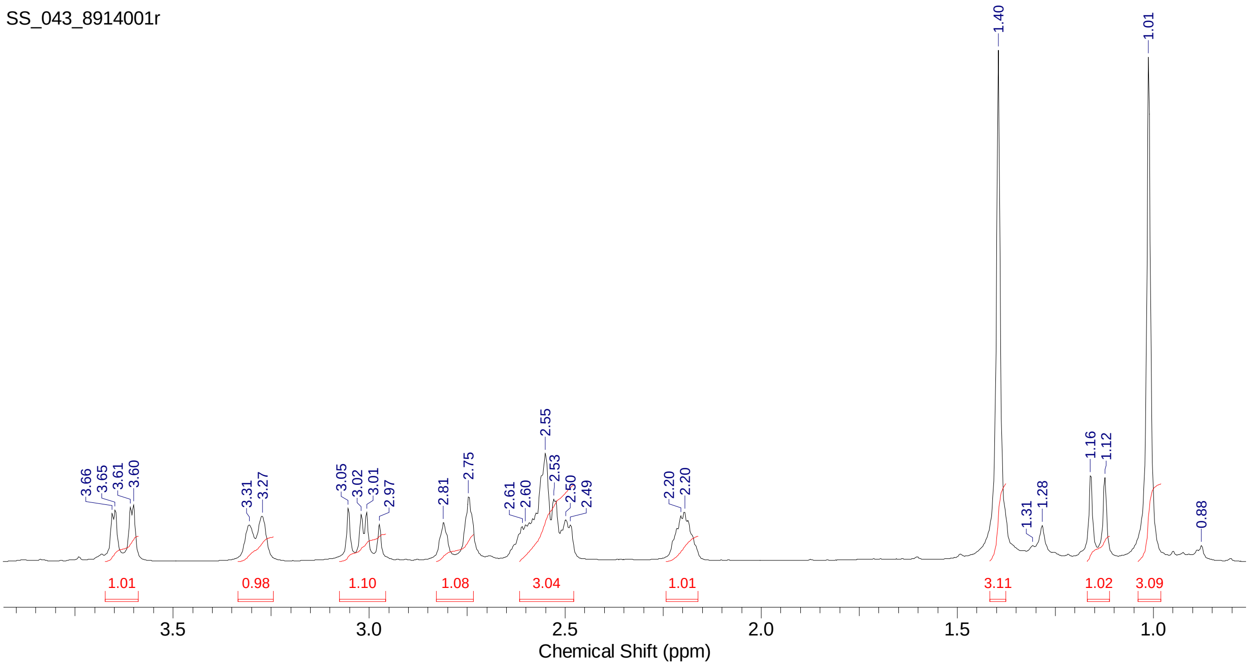

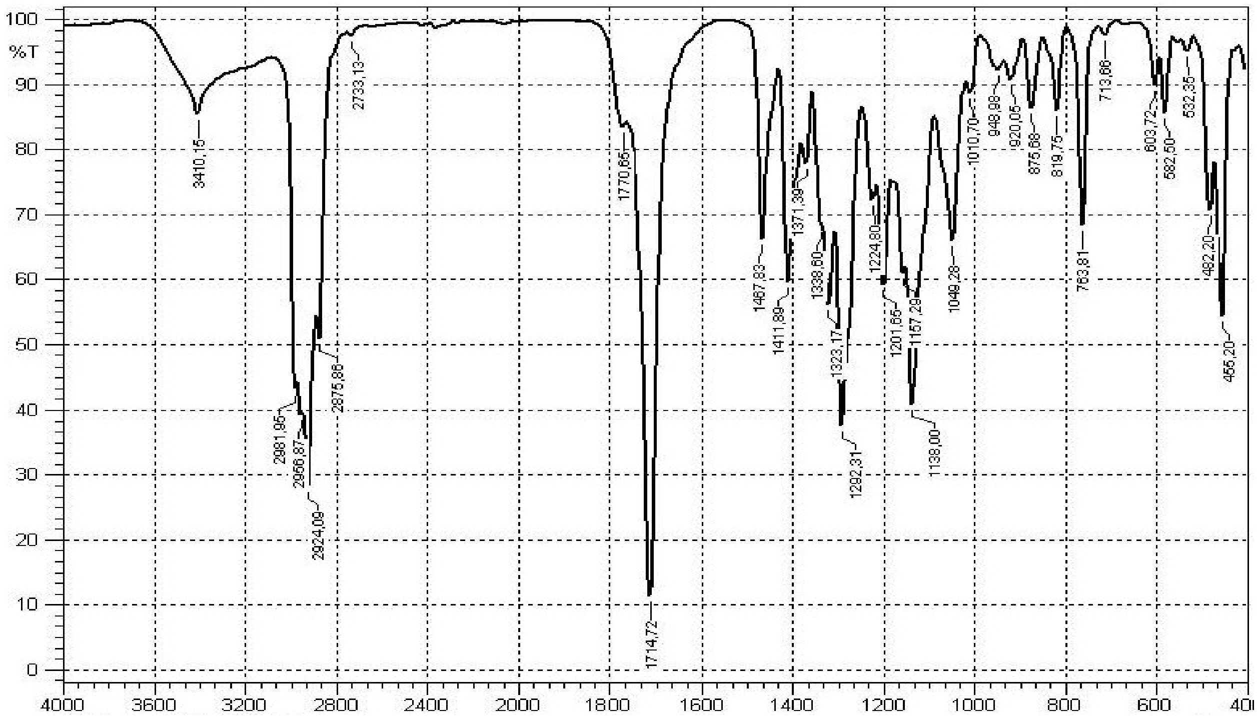

%T
3410.15
2981.95
2956.87
2924.09
2875.86
2733.13
1770.65
1714.72
1467.83
1411.89
1371.39
1339.60
1323.17
1292.31
1224.80
1201.65
1157.29
1138.00
1049.28
1010.70
948.98
920.05
875.68
819.75
763.81
713.66
603.72
582.50
532.35
482.20
455.20

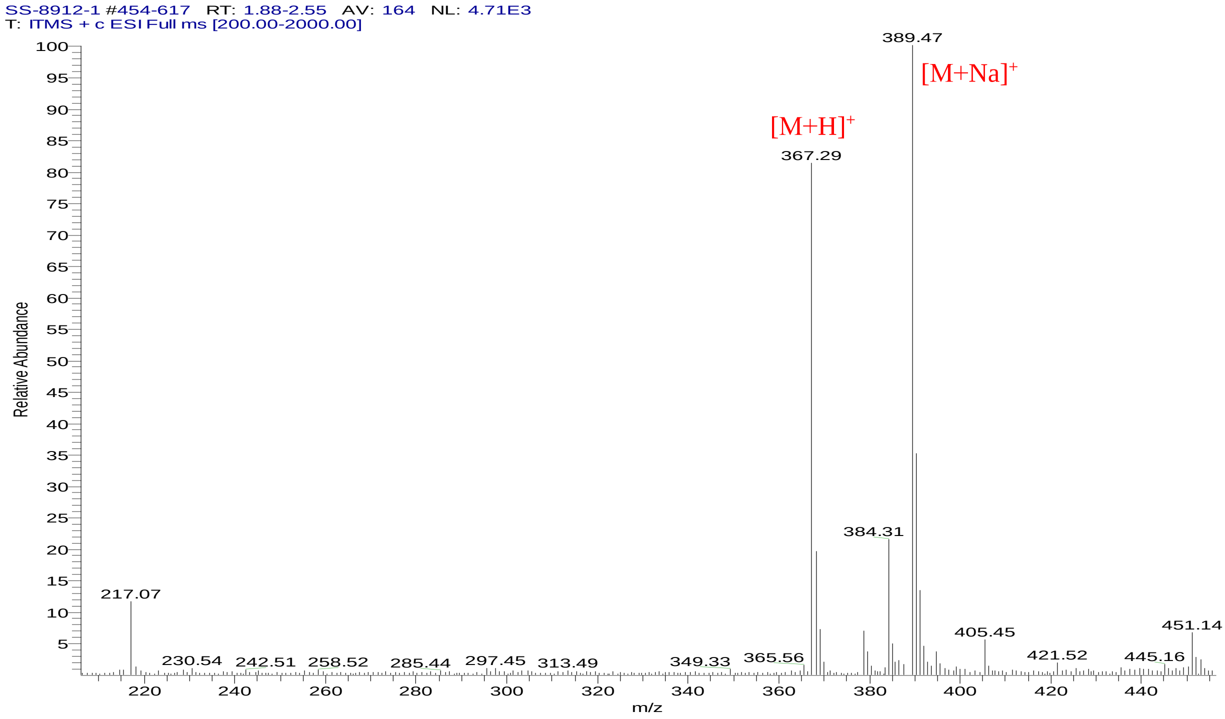

SS-8912-1 #454-617 RT: 1.88-2.55 AV: 164 NL: 4.71E3
T: ITMS + c ESI Full ms [200.00-2000.00]
Relative Abundance
m/z
[M+Na]⁺
[M+H]⁺
389.47
367.29
384.31
217.07
230.54
242.51
258.52
285.44
297.45
313.49
349.33
365.56
405.45
421.52
445.16
451.14

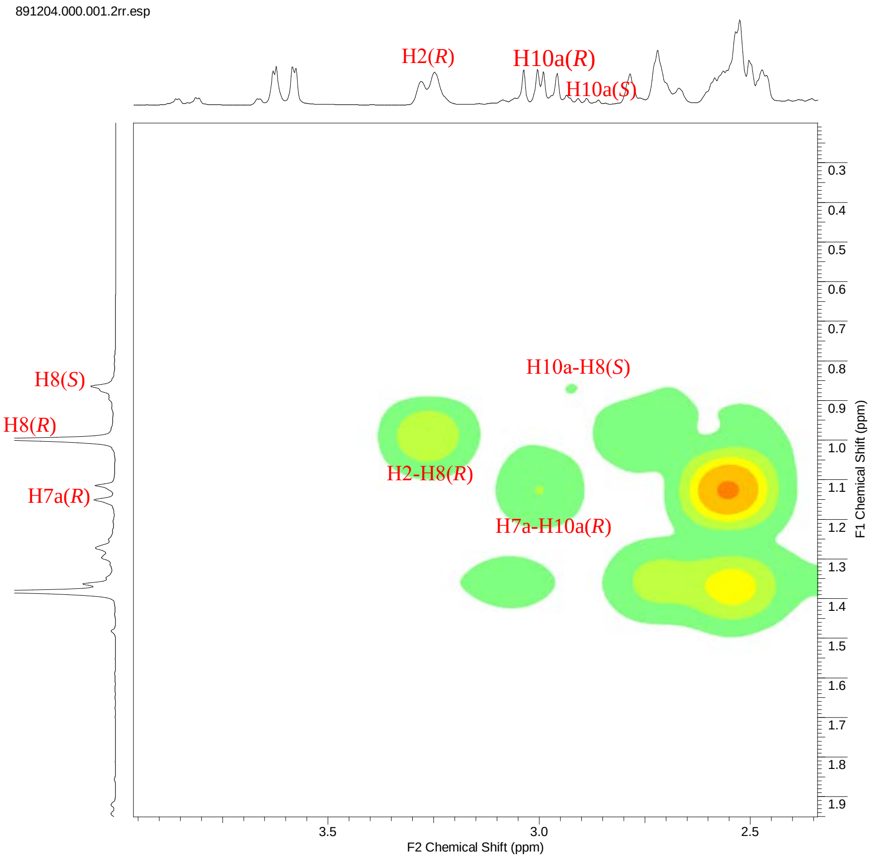

Fig. 1. *NOE*-interactions of H2–H8, H7a–H10a for (2*R*)-**16** and H10a–H8 for (2*S*)-**16** in the spectrum of mixture of sulfones.

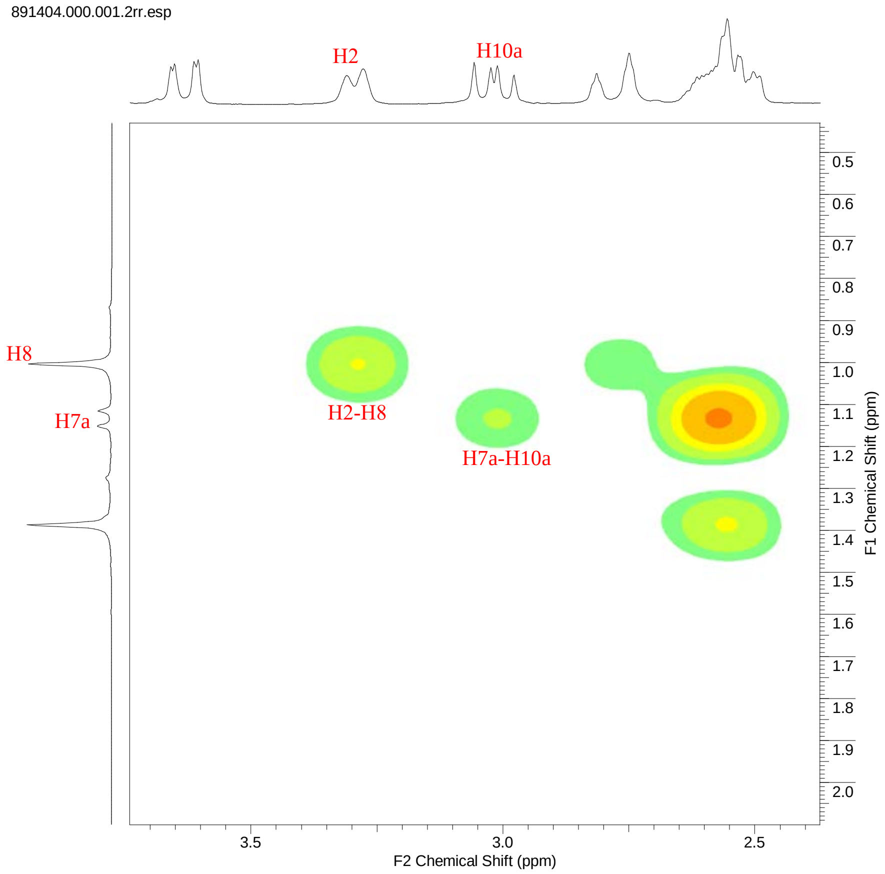

Fig. 2. *NOE*-interactions of H2–H8, H7a–H10a in the spectrum of (2*R*)-sulfone **16**.

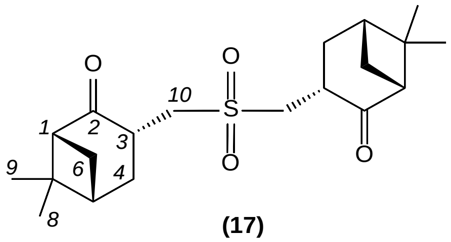
O
O
10
S
O
O
1
2
3
9
6
4
8

(17)

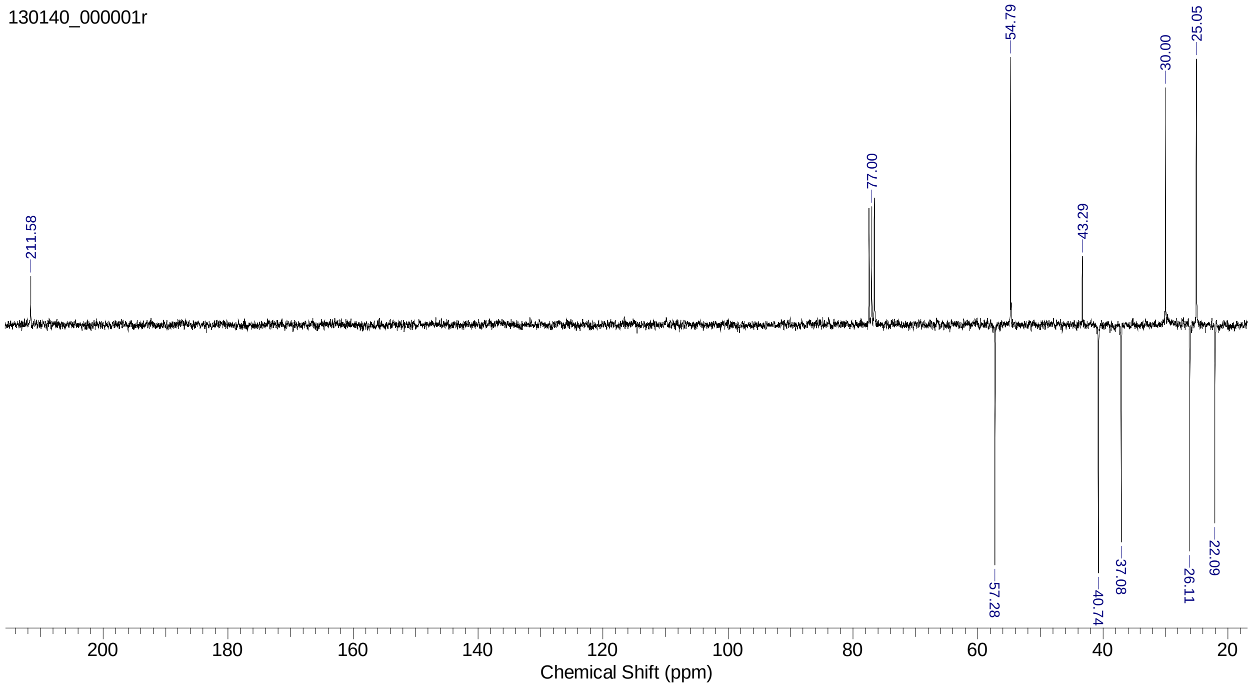
130140_000001r
211.58
77.00
54.79
43.29
30.00
25.05
57.28
40.74
37.08
26.11
22.09
200
180
160
140
120
100
80
60
40
20
Chemical Shift (ppm)

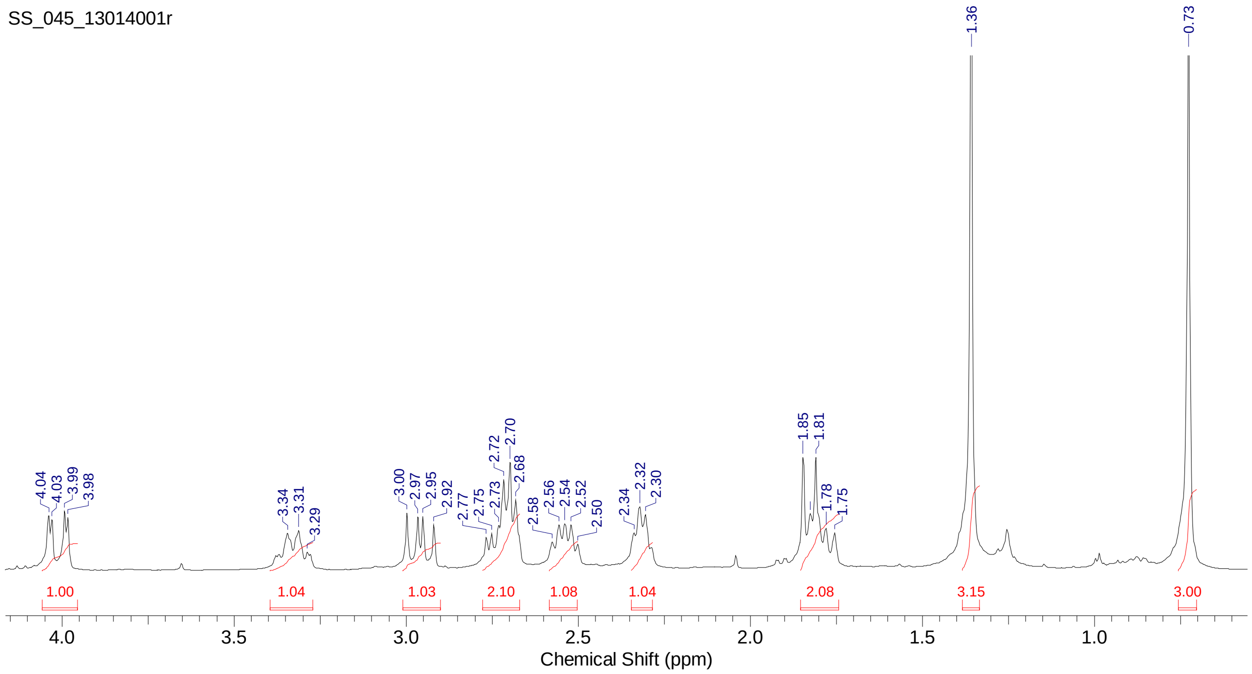
SS_045_13014001r
1.36
0.73
4.04
4.03
3.99
3.98
3.34
3.31
3.29
3.00
2.97
2.95
2.92
2.77
2.75
2.73
2.72
2.70
2.68
2.58
2.56
2.54
2.52
2.50
2.34
2.32
2.30
1.85
1.81
1.78
1.75
1.00
1.04
1.03
2.10
1.08
1.04
2.08
3.15
3.00
4.0
3.5
3.0
2.5
2.0
1.5
1.0
Chemical Shift (ppm)

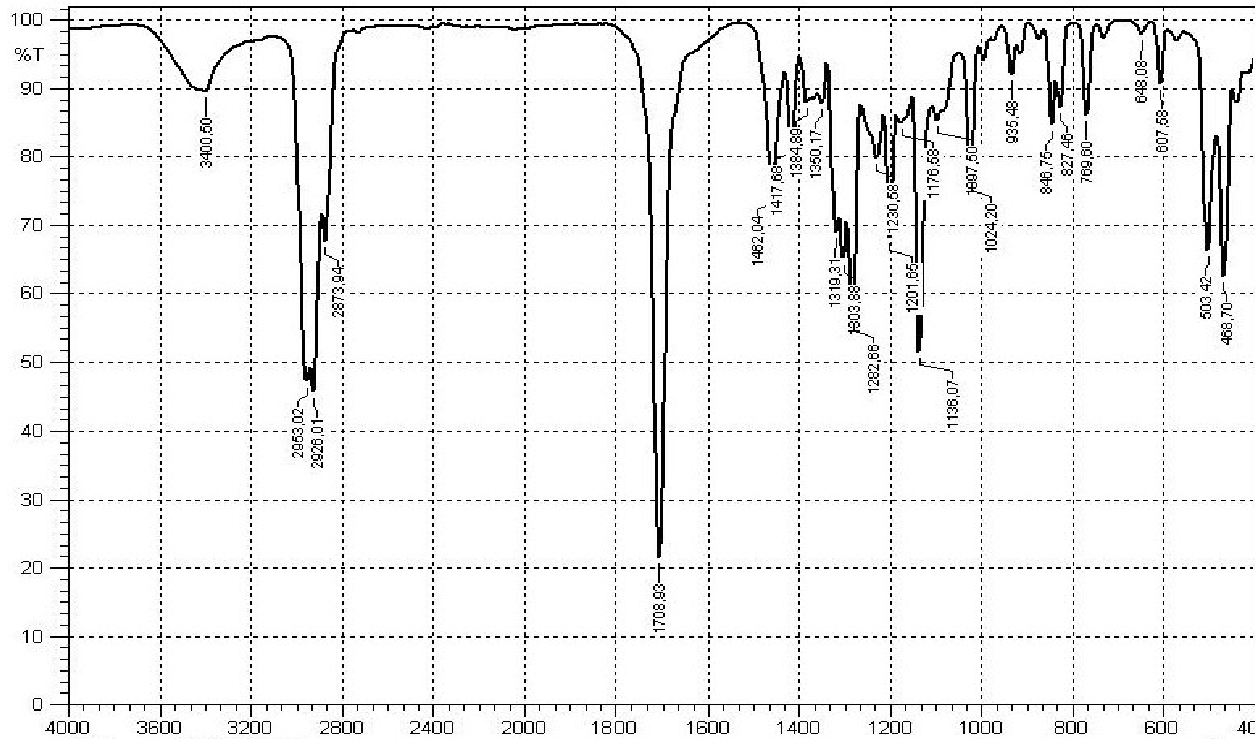

%T
3400,50
2953,02
2926,01
2873,94
1708,93
1462,04
1417,68
1384,89
1350,17
1319,31
1303,88
1282,66
1230,58
1201,65
1176,58
1138,07
1097,50
1024,20
935,48
846,75
827,46
769,60
648,08
607,58
503,42
468,70

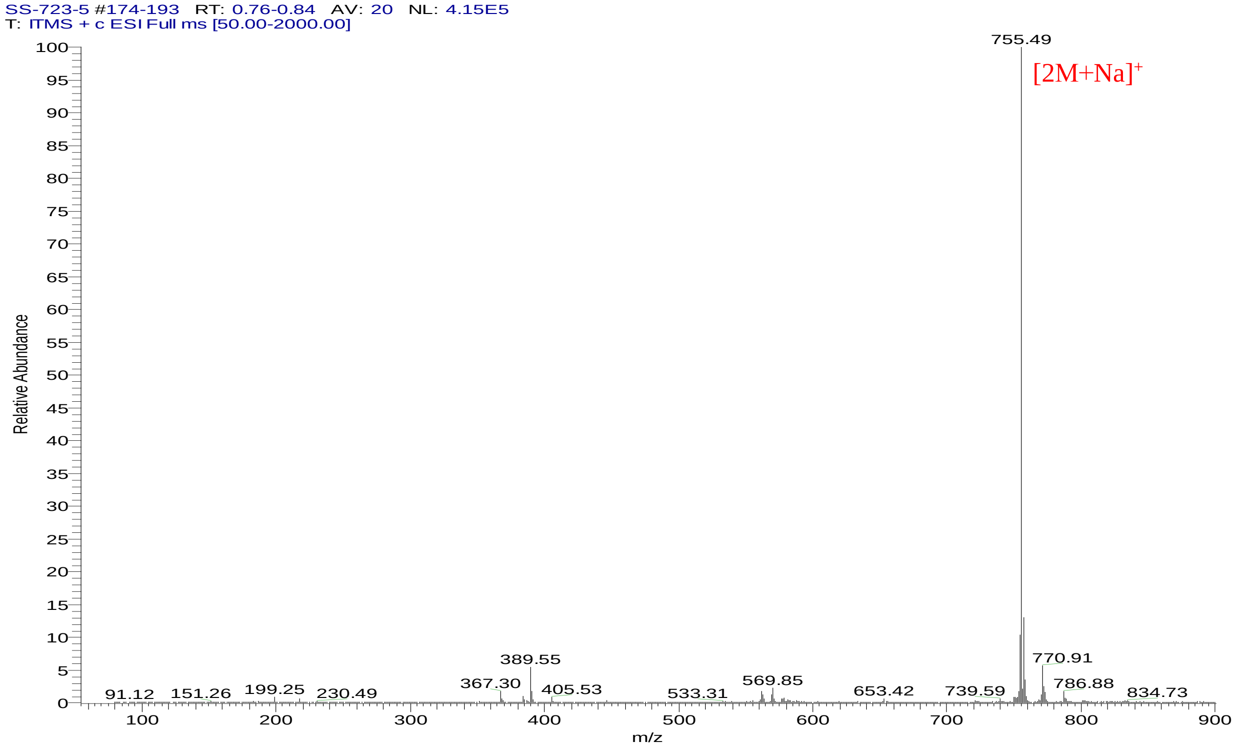

SS-723-5 #174-193 RT: 0.76-0.84 AV: 20 NL: 4.15E5
T: ITMS + c ESI Full ms [50.00-2000.00]
Relative Abundance
m/z
755.49
[2M+Na]+
389.55
367.30
405.53
569.85
533.31
653.42
739.59
770.91
786.88
834.73
91.12
151.26
199.25
230.49

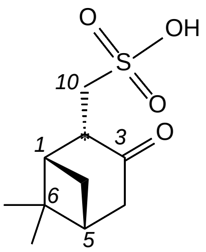
O
OH
S
O
10
3
O
1
6
5

• 5 Py

(2*S*)-18

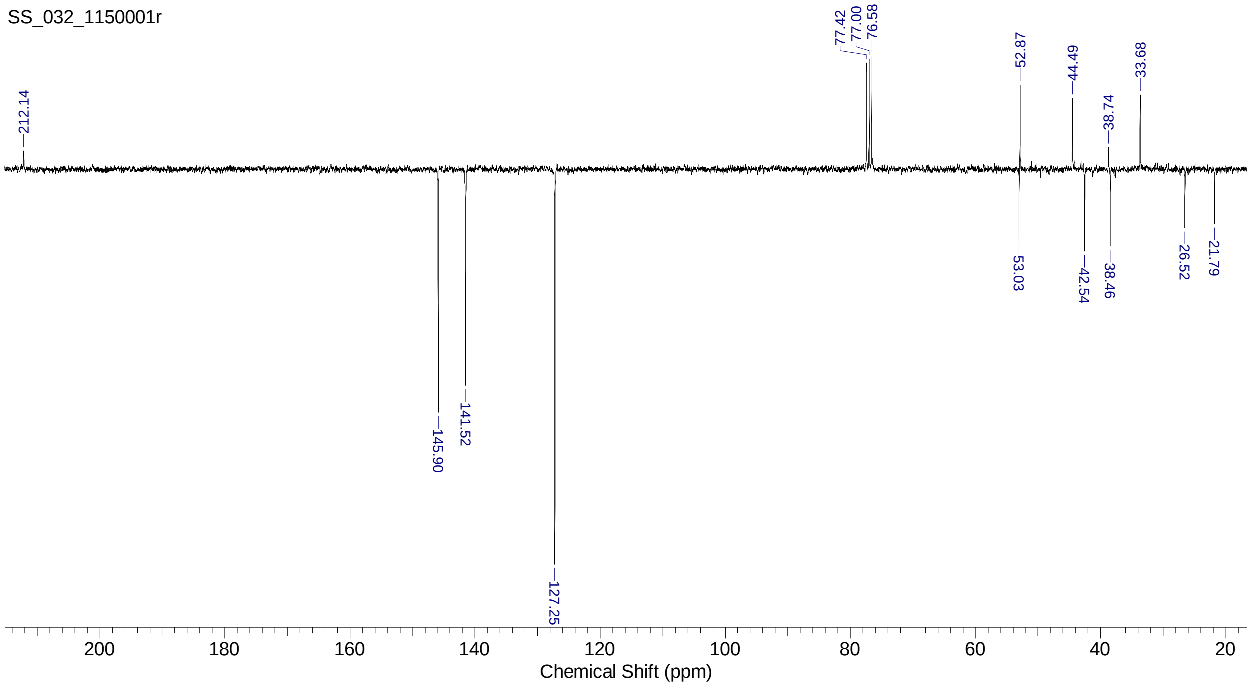
SS_032_1150001r
212.14
77.42
77.00
76.58
52.87
44.49
38.74
33.68
145.90
141.52
127.25
53.03
42.54
38.46
26.52
21.79
200
180
160
140
120
100
80
60
40
20
Chemical Shift (ppm)

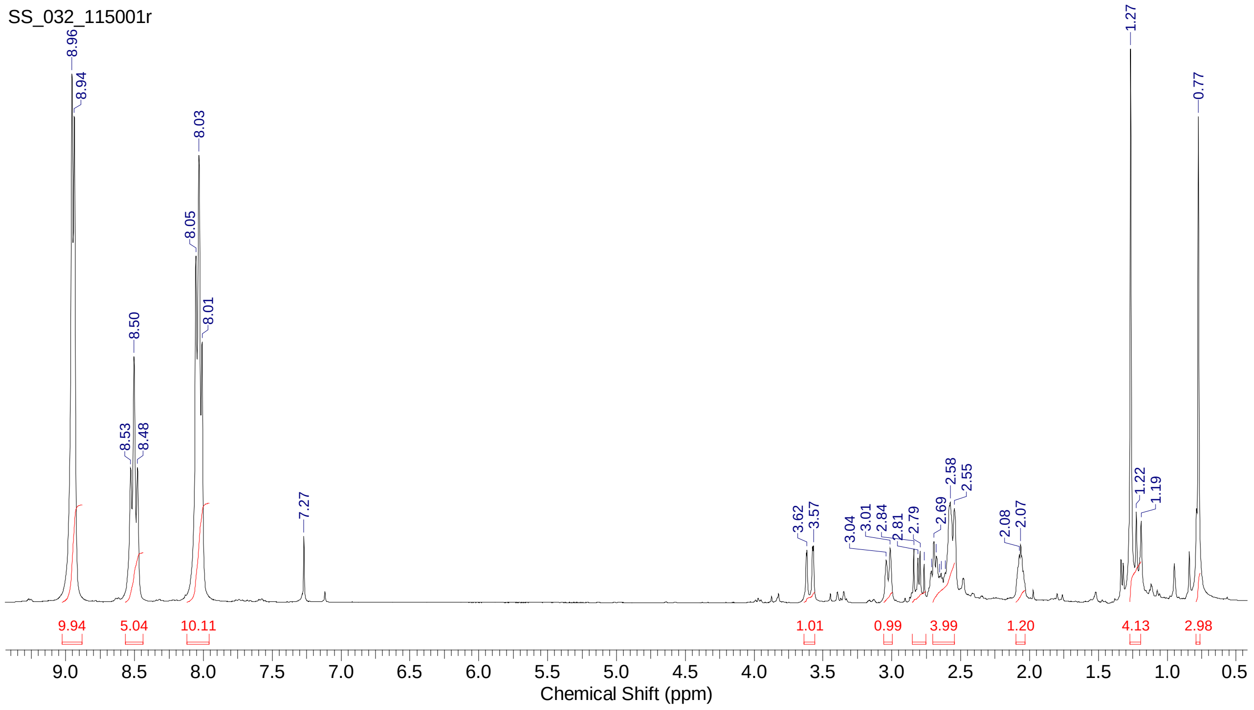
SS_032_115001r
8.96
8.94
8.53
8.50
8.48
8.05
8.03
8.01
7.27
3.62
3.57
3.04
3.01
2.84
2.81
2.79
2.69
2.58
2.55
2.08
2.07
1.27
1.22
1.19
0.77
9.94
5.04
10.11
1.01
0.99
3.99
1.20
4.13
2.98
9.0
8.5
8.0
7.5
7.0
6.5
6.0
5.5
5.0
4.5
4.0
3.5
3.0
2.5
2.0
1.5
1.0
0.5
Chemical Shift (ppm)

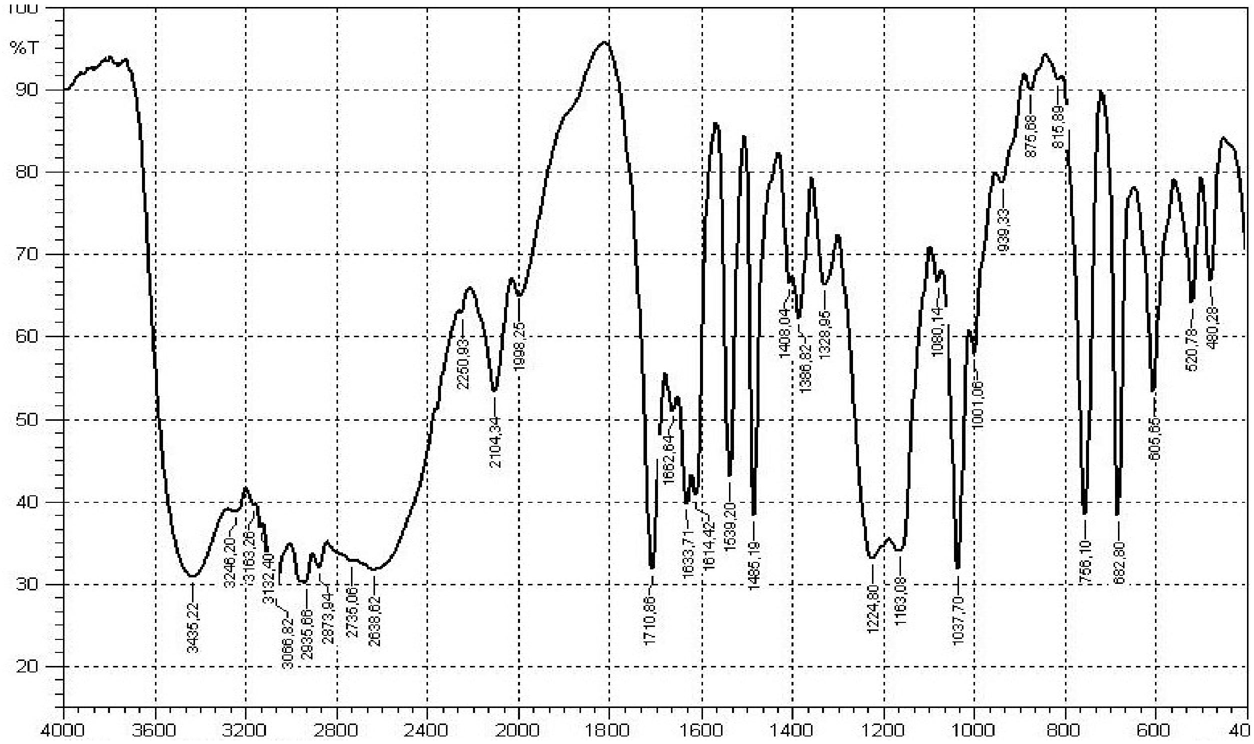
%T
3435,22
3246,20
3163,26
3132,40
3066,82
2935,66
2873,94
2735,06
2638,62
2250,93
2104,34
1998,25
1710,86
1662,64
1633,71
1614,42
1539,20
1485,19
1408,04
1386,82
1328,95
1224,80
1163,08
1080,14
1037,70
1001,06
939,33
875,68
815,89
756,10
682,80
605,65
520,78
480,28

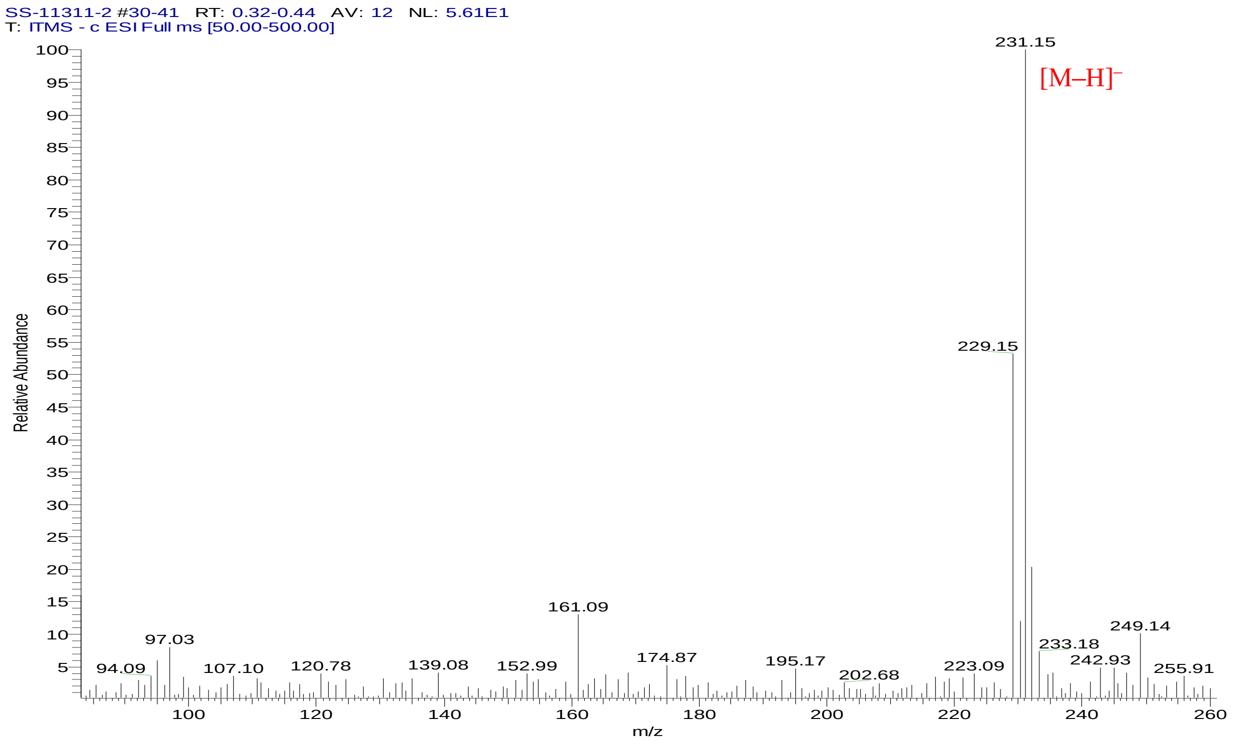
SS-11311-2 #30-41 RT: 0.32-0.44 AV: 12 NL: 5.61E1
T: ITMS - c ESI Full ms [50.00-500.00]
231.15
[M–H]⁻
229.15
161.09
249.14
233.18
242.93
255.91
97.03
94.09
107.10
120.78
139.08
152.99
174.87
195.17
202.68
223.09
Relative Abundance
m/z

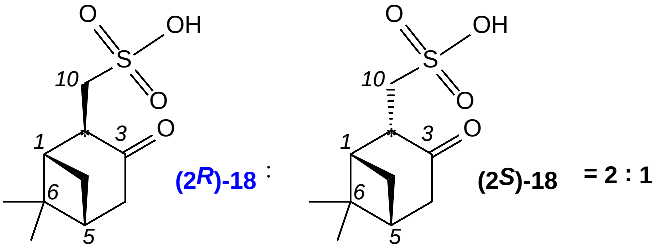

(2*R*)-**18** : (2*S*)-**18** = 2 : 1

SS_032_11310001r

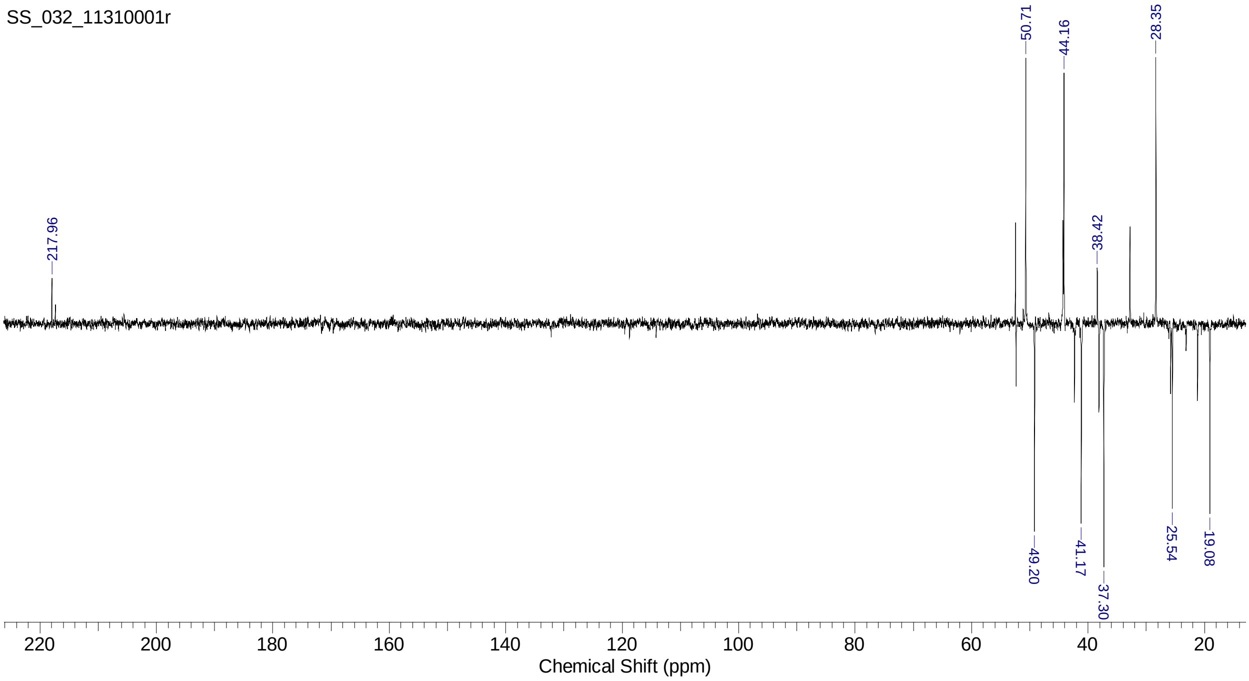

SS_031_1121001r

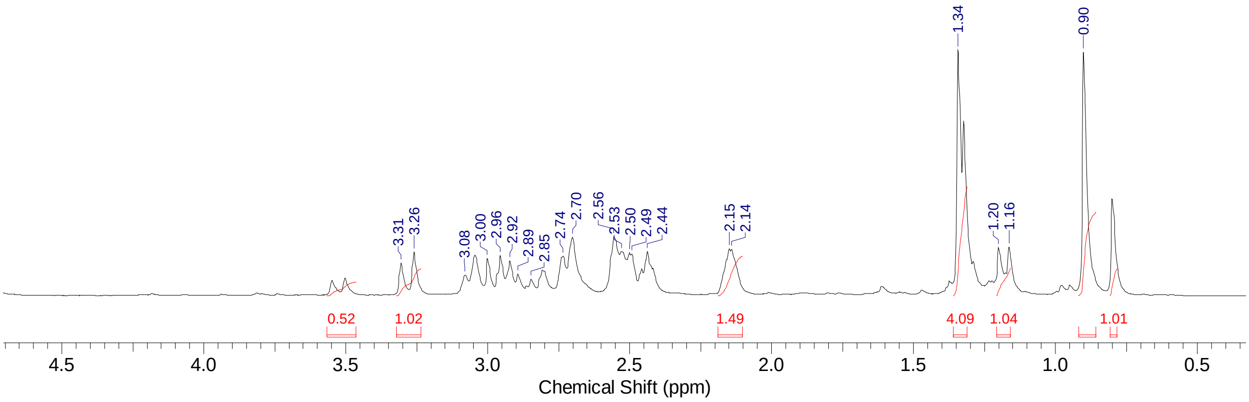

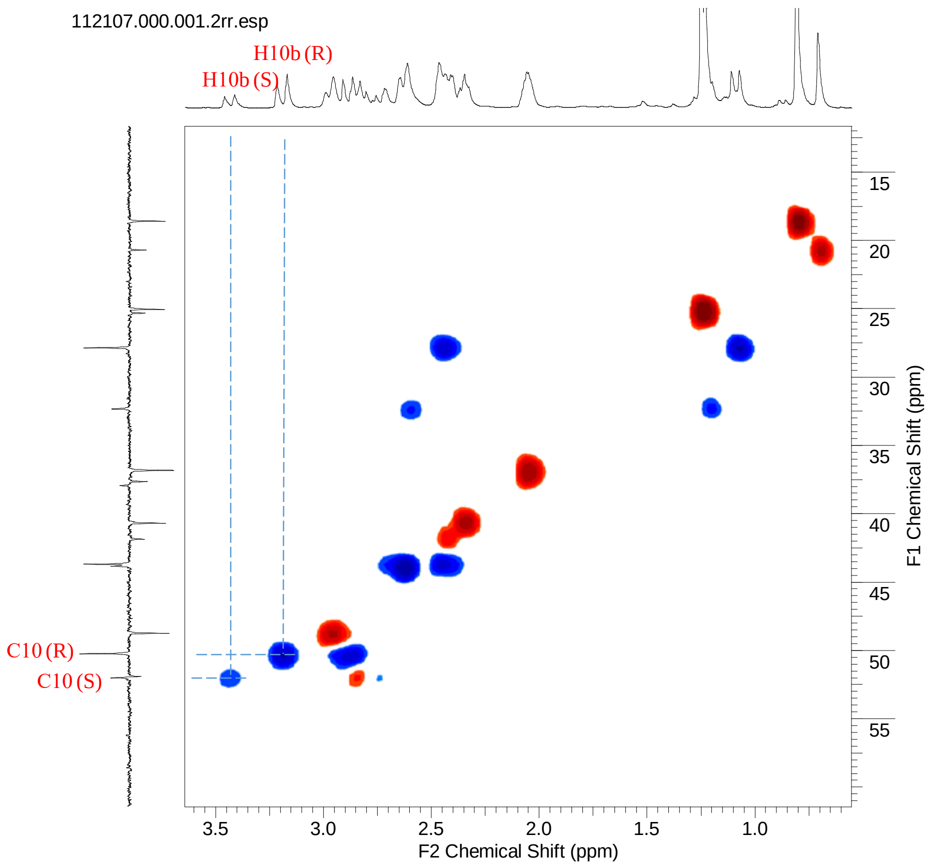

Fig. 3. HSQC-interactions of C10–H10b atoms in the spectra of (2*S*)-**18** and (2*R*)-**18** sulfonic acids.

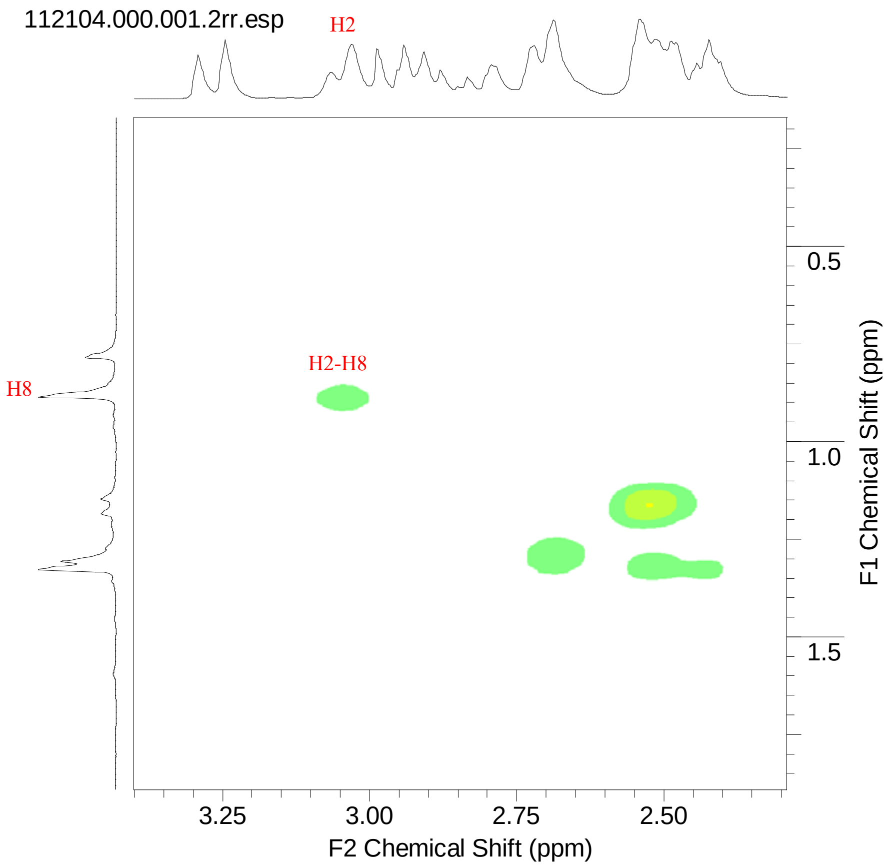

Fig. 4. *NOE*-interactions of H2-H8 in the spectrum of acid (2*R*)-**18**.

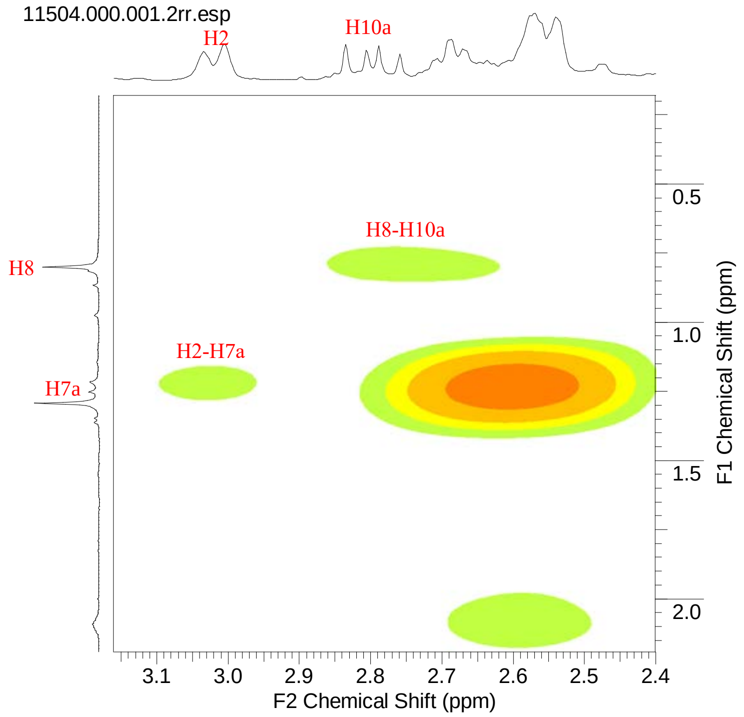

Fig. 5. *NOE*-interactions of H2-H7β and H8-H10a in the spectrum of acid (2S)-**18**.

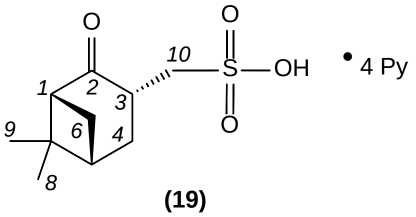
O
O
O
10
S
OH
• 4 Py
1
2
3
9
6
4
8

(19)

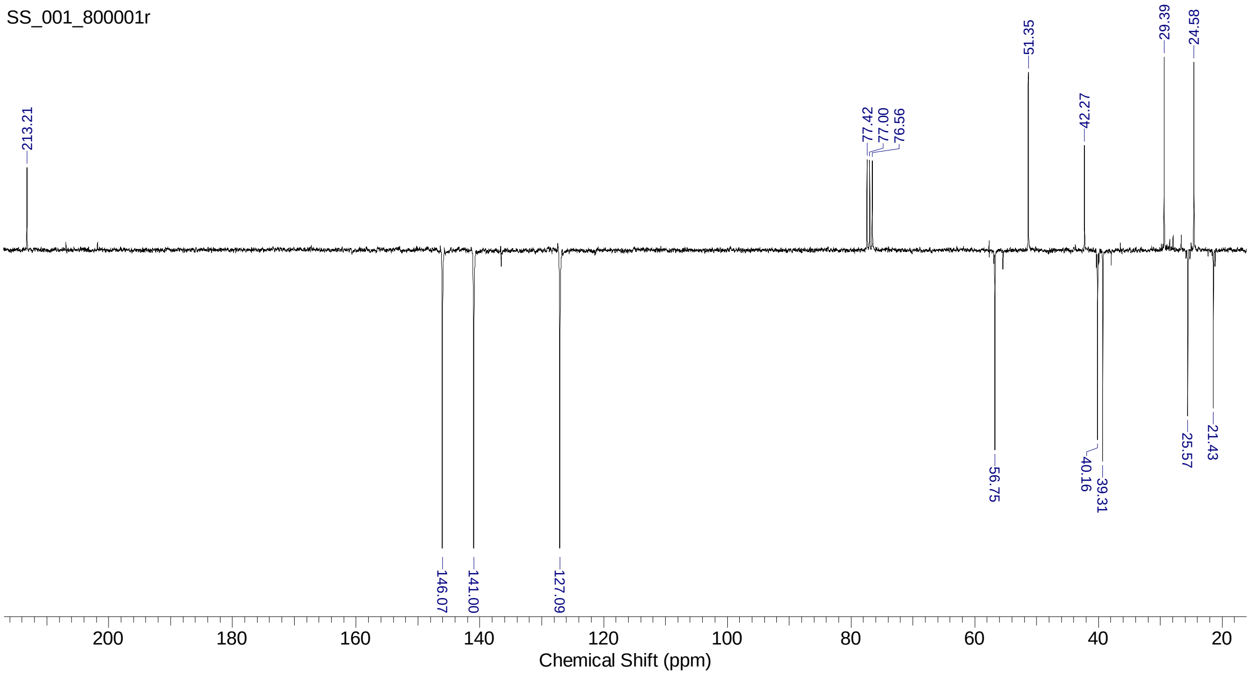
SS_001_800001r
213.21
77.42
77.00
76.56
51.35
42.27
29.39
24.58
146.07
141.00
127.09
56.75
40.16
39.31
25.57
21.43
Chemical Shift (ppm)

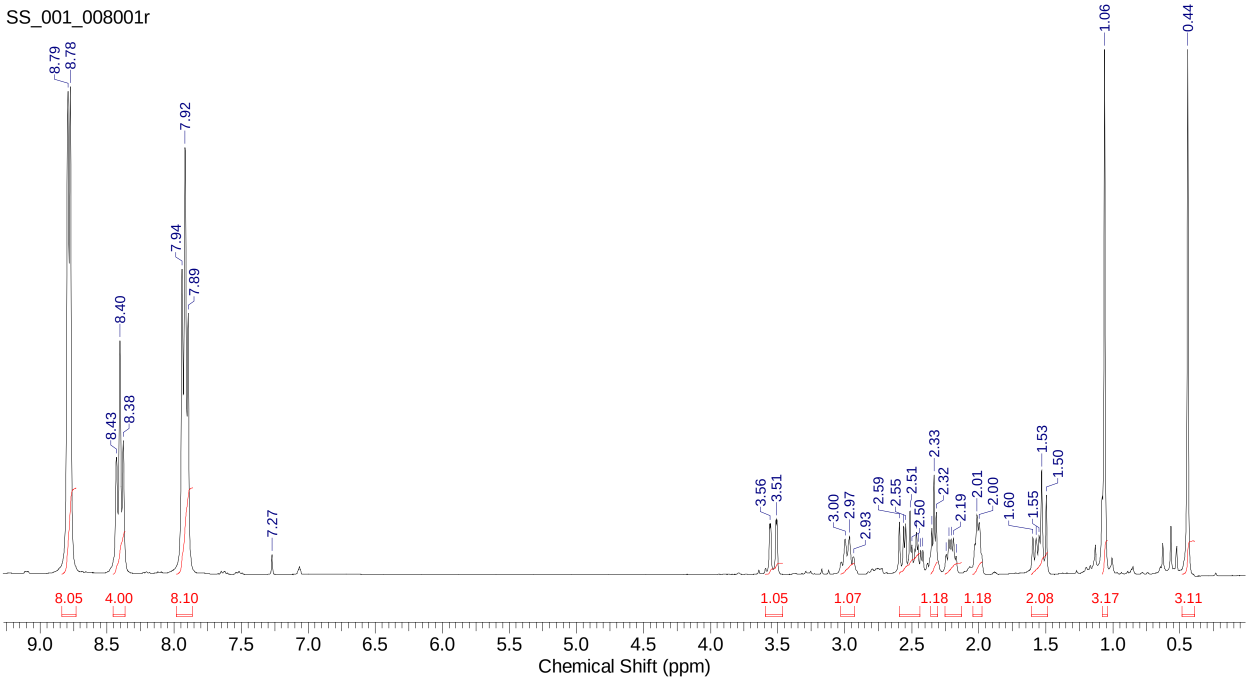
SS_001_008001r
8.79
8.78
8.43
8.40
8.38
7.94
7.92
7.89
7.27
3.56
3.51
3.00
2.97
2.93
2.59
2.55
2.51
2.50
2.33
2.32
2.19
2.01
2.00
1.60
1.55
1.53
1.50
1.06
0.44
8.05
4.00
8.10
1.05
1.07
1.18
1.18
2.08
3.17
3.11
Chemical Shift (ppm)

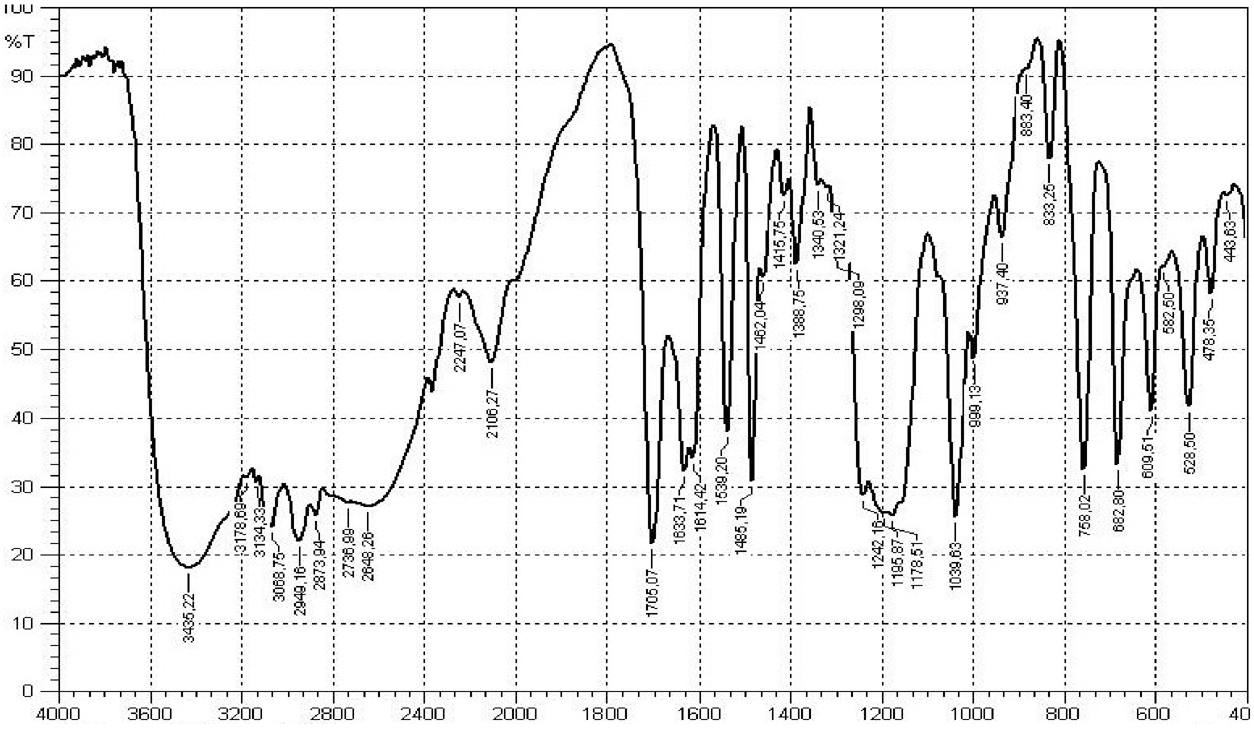
%T
3435,22
3178,69
3134,33
3068,75
2949,16
2873,94
2736,89
2648,26
2247,07
2106,27
1705,07
1633,71
1614,42
1539,20
1485,19
1452,04
1415,75
1388,75
1340,53
1321,24
1298,09
1242,16
1195,87
1178,51
1039,63
999,13
937,40
883,40
833,25
758,02
682,80
609,51
582,50
528,50
478,35
443,63

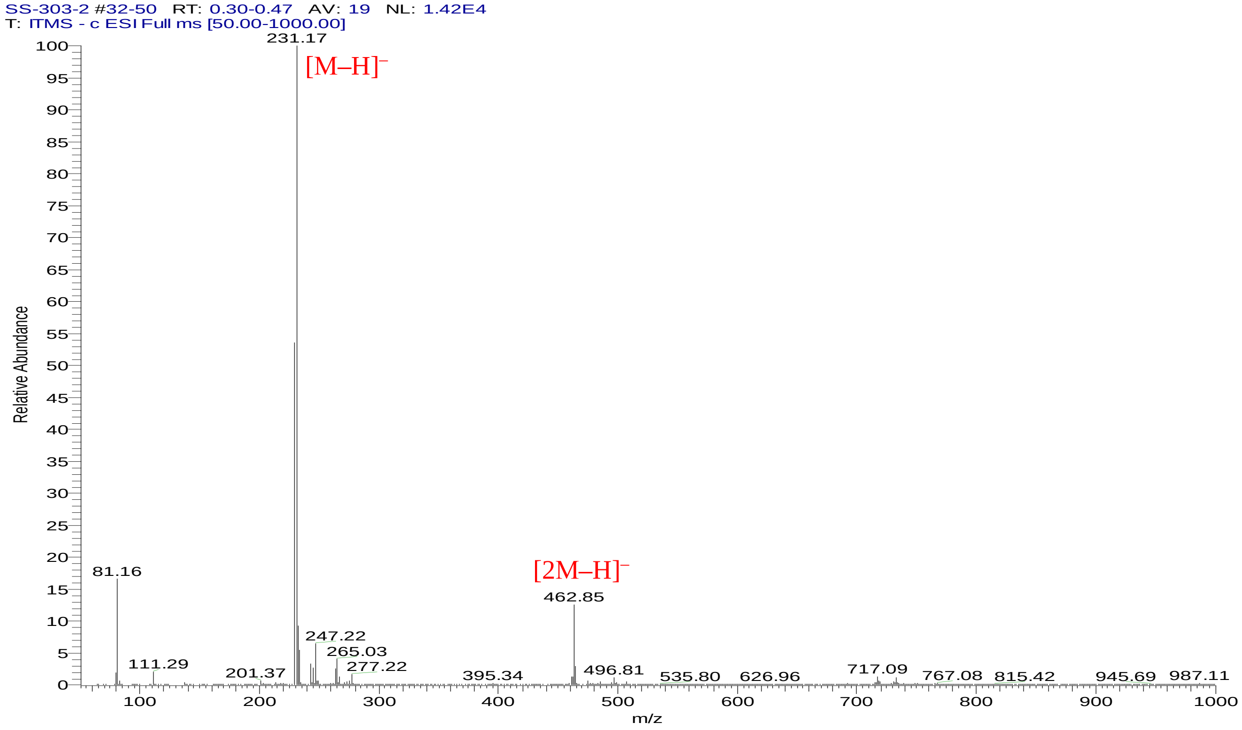
SS-303-2 #32-50 RT: 0.30-0.47 AV: 19 NL: 1.42E4
T: ITMS - c ESI Full ms [50.00-1000.00]
Relative Abundance
m/z
231.17
[M–H]⁻
[2M–H]⁻
462.85
81.16
111.29
201.37
247.22
265.03
277.22
395.34
496.81
535.80
626.96
717.09
767.08
815.42
945.69
987.11
